# Multilayered Tuning of Dosage Compensation and Z-Chromosome Masculinization in the Wood White (*Leptidea sinapis*) Butterfly

**DOI:** 10.1093/gbe/evz176

**Published:** 2019-08-10

**Authors:** Lars Höök, Luis Leal, Venkat Talla, Niclas Backström

**Affiliations:** 1 Evolutionary Biology Program, Department of Ecology and Genetics, Evolutionary Biology Centre (EBC), Uppsala University, Sweden; 2 Plant Ecology and Evolution, Department of Ecology and Genetics, Evolutionary Biology Centre (EBC), Uppsala University, Sweden

**Keywords:** Lepidoptera, dosage compensation, sex-chromosomes, sex-biased gene expression, female heterogamety

## Abstract

In species with genetic sex determination, dosage compensation can evolve to equal expression levels of sex-linked and autosomal genes. Current knowledge about dosage compensation has mainly been derived from male-heterogametic (XX/XY) model organisms, whereas less is understood about the process in female-heterogametic systems (ZZ/ZW). In moths and butterflies, downregulation of Z-linked expression in males (ZZ) to match the expression level in females (ZW) is often observed. However, little is known about the underlying regulatory mechanisms, or if dosage compensation patterns vary across ontogenetic stages. In this study, we assessed dynamics of Z-linked and autosomal expression levels across developmental stages in the wood white (*Leptidea sinapis*). We found that although expression of Z-linked genes in general was reduced compared with autosomal genes, dosage compensation was actually complete for some categories of genes, in particular sex-biased genes, but equalization in females was constrained to a narrower gene set. We also observed a noticeable convergence in Z-linked expression between males and females after correcting for sex-biased genes. Sex-biased expression increased successively across developmental stages, and male-biased genes were enriched on the Z-chromosome. Finally, all five core genes associated with the ribonucleoprotein dosage compensation complex male-specific lethal were detected in adult females, in correspondence with a reduction in the expression difference between autosomes and the single Z-chromosome. We show that tuning of gene dosage is multilayered in Lepidoptera and argue that expression balance across chromosomal classes may predominantly be driven by enrichment of male-biased genes on the Z-chromosome and cooption of available dosage regulators.

## Introduction

Genetic sex determination is common in the animal kingdom and often regulated by dimorphic sex-chromosomes for which one sex is homogametic and the other heterogametic (Charlesworth 1991; Bachtrog et al. 2011). Various sex-chromosome systems have evolved independently across different lineages, the most well known being the male-heterogametic XX/XY system in mammals and *Drosophila* (Abbott et al. 2017), and the female-heterogametic ZZ/ZW system in birds (Smith and Sinclair 2004), snakes (Rovatsos et al. 2015), and Lepidoptera (moths and butterflies) (Traut et al. 2007). The development of sex-chromosomes from autosomes is hypothesized to be driven by selection for reduced recombination as a way to resolve sexual conflict (Charlesworth 1991). The rationale is that selection should act to enhance genetic linkage between a sex-determining locus and genes with sexually antagonistic effects, a process that promotes chromosomal inversions that reduce recombination between the sex-determination locus and loci with conflicting effects in each respective sex (Bergero and Charlesworth 2009; Wright et al. 2017). The reduction in recombination will ultimately lead to successively increased differentiation of the sex-chromosome pair (X and Y or Z and W), resulting in a gradual degeneration of the sex-limited chromosome (Y or W) due to less efficient selection against deleterious mutations (Bergero and Charlesworth 2009). When genes on the sex-limited chromosome have started to degenerate, gene dosage of sex-linked genes can be altered as compared with ancestral levels in the heterogametic sex. Gene expression levels generally correspond to gene dose (Pollack et al. 2002) and changing the copy number of genes in an already established genetic context can result in negative epistatic stoichiometry (Veitia and Potier 2015). However, the functional effects of dose change may vary depending on the role of the gene in regulatory networks (Malone et al. 2012) and/or its expression level and breadth (Gout et al. 2010). Regardless, the accumulated effect of losing the function of many genes once present on the ancestral Y- or W-chromosome should impose deleterious effects on the heterogametic sex (Ellegren 2011; Bachtrog 2013). As a counteracting measure, dosage compensation can evolve to retain ancestral (prior to sex-chromosome differentiation) transcription levels of sex-linked genes and to balance the overall expression of sex-linked genes between males and females (Ohno 1967; Charlesworth 1978; Gu and Walters 2017). Previous research in various species has revealed different ways in which sex-chromosome expression can be regulated (Mank 2013). In *Drosophila melanogaster*, balance is achieved by doubling the X-linked expression in males (Straub et al. 2005). In the nematode *Caenorhabditis elegans*, upregulation of X-linked genes in both sexes is combined with reduced expression of both X-chromosomes in hermaphrodites (Gupta et al. 2006). Similarly, there is a general upregulation of X-chromosomes in humans but the dosage is adjusted by inactivation of one of the X-chromosomes in females (Brockdorff and Turner 2015). Birds (ZW) seem to lack a global dosage compensation mechanism altogether (Ellegren et al. 2007) and may instead regulate dose sensitive genes individually (Mank and Ellegren 2009; Zimmer et al. 2016).

The rapid advance of high-throughput sequencing methods has spurred research beyond traditional model organisms and expanded the phylogenetic coverage, allowing for a better understanding of how dosage compensation has evolved in different lineages (Wolf and Bryk 2011). Over the last decade, there has been an increased interest in studying dosage compensation in lepidopteran species using RNA-seq data. These genomic approaches have shifted the view that dosage compensation is absent in Lepidoptera to being a more open question as dosage regulation has been observed in some taxa but not in others. The Indian meal moth, *Plodia interpunctella*, initially appeared to lack dosage compensation altogether (Harrison et al. 2012). However, when reanalyzing *P. interpunctella* data excluding sex-biased genes, the Z-linked expression was found to be equalized between males and females and lower compared with autosomes (Huylmans et al. 2017). Similar patterns have been observed in *Bombyx mori* (Gopinath et al. 2017; Huylmans et al. 2017), *Cydia pomonella* (Gu et al. 2017), and *Papilio xuthus* (Huylmans et al. 2017). Likewise, several *Heliconius* species show a similar Z-chromosome to autosomal imbalance, but in addition a weak male-bias in Z-chromosome expression (Walters et al. 2015; Catalan et al. 2018). Thus, the most common pattern in Lepidoptera, referred to as incomplete dosage compensation (Gu and Walters 2017), suggests a mechanism where the male Z-chromosome expression is reduced to match female Z-chromosome expression, but with no equalization between sex-chromosomes and autosomes. So far, two exceptions invoking complete dosage compensation have been observed in the lepidopterans *Manduca sexta* (Smith et al. 2014) and *Papilio machaon* (Huylmans et al. 2017), indicating that incomplete dosage compensation is not ubiquitous across Lepidoptera and that extensive additional taxonomic sampling is needed to get a more complete picture.

Substantial changes in overall gene expression between developmental stage transitions has previously been observed (e.g., Leal et al. 2018), but the impact of metamorphosis on temporal patterns of dosage compensation has not been thoroughly analyzed in any lepidopteran species. In this study, we used RNA-seq data to analyze dosage compensation in the wood white butterfly, *Leptidea sinapis* (Pieridae), across three ontogenetic stages: instar-V larva (from here on larva), pupa, and imago (adult). This is the first attempt to assess dosage compensation in the family Pieridae and to detail potential variation in sex-biased expression and patterns of dosage compensation across ontogenetic stages. The analyses not only reveal a general reduction in expression of a majority of Z-linked genes in both sexes but also complete dosage compensation for particular gene categories: in male wood whites, there is complete Z:autosome dosage compensation between sex-biased genes, as well as between Z-linked genes and unbiased autosomal genes; in females, there is complete dosage compensation between Z-linked female-biased genes and autosomal sex-biased genes. Sex-biased expression increased progressively from larvae to adults, and the Z-chromosome was significantly enriched for male-biased genes. Importantly, we found evidence of a ribonucleoprotein male-specific lethal (MSL) complex active exclusively in adult females, in line with an observed reduction in autosome to Z-chromosome expression ratio. We conclude that tuning of gene dosage in Lepidoptera is multilayered and more complex than previously recognized and argue that this could be a result of Z-chromosome masculinization followed by cooption of available ribonucleoprotein dosage regulators in females.

## Materials and Methods

### Quality Filtering

Gene expression levels were assessed using recently published RNA-seq data obtained for distinct developmental stages of *L. sinapis* (Leal et al. 2018). In short, total RNA was prepared from whole body samples of Swedish *L. sinapis* reared in captivity in 2016 and sequenced by NGI Stockholm using Illumina HiSeq2500. The raw data contained paired-end short reads (2× 126 bp) with an estimated average coverage of 334× per gene per individual. Three developmental stages (larva, pupa, and adult) were analyzed using three males and three females from each respective developmental stage as biological replicates. All specimens were the offspring of four different *L. sinapis* females (henceforth referred to as families). A previously established filtering pipeline (Leal et al. 2018) was implemented to remove low-quality reads and nucleotides. In brief, quality assessment of raw reads was done with FASTQC, version 0.11.5 (https://www.bioinformatics.babraham.ac.uk/projects/fastqc/, accessed: April 1, 2018), and terminal end nucleotides were trimmed (12 bases from the 5′-end and 3 bases from the 3′-end) using TRIM GALORE, version 0.4.1 (https://www.bioinformatics.babraham.ac.uk/projects/trim_galore/, accessed: April 1, 2018). This package was also used to remove known adapter sequences and low quality (<30 Phred) bases remaining at read ends. Internal low-quality nucleotides (Phred score < 20) were masked (N) with Fastq Masker, as implemented in the FASTX-Toolkit, version 0.0.14 (http://hannonlab.cshl.edu/fastx_toolkit/index.html, accessed: January 29, 2018). Poly-A tails and homopolymers were filtered out using PRINSEQ, version 0.20.4 (Schmieder and Edwards 2011), and reads with >10% masked nucleotides were subsequently removed from the data set. Finally, short reads (<30 nucleotides) and reads with an overall low quality (Phred score < 30 in more than 20% of nucleotide positions) were removed using CONDETRI, version 2.3 (Smeds and Künstner 2011). In total, quality filtering reduced the average read length from 126 to 107 bp. After filtering, different libraries originating from the same biological replicate were concatenated. Screening for contaminants was performed with FASTQ SCREEN, version 0.9.2 (https://www.bioinformatics.babraham.ac.uk/projects/fastq_screen/, accessed: April 1, 2018) and BOWTIE2, version 2.2.9 (Langmead and Salzberg 2012). Libraries used for contaminant screening included rRNA sequences obtained from the SILVA and Rfam databases (Quast et al. 2012; Nawrocki et al. 2015), *Wolbachia* sp. (Kersey et al. 2016), the GRCh38 human assembly (Schneider et al. 2017), and previously characterized *L. sinapis* repeat sequences (Talla et al. 2017). The amount of removed potential contaminant reads, mostly rRNA, was at most 2.6% of the reads in a particular library.

### Read Mapping, Assembly, and Calculation of Expression Levels

Reads were mapped to the *L. sinapis* draft genome assembly (7,090 scaffolds with N50 = 0.857 Mb; Talla et al. 2017) following a standard protocol for STAR, version 2.5.3a (Dobin and Gingeras 2015) with default settings. For all samples, more than 80% (mean = 89.3%) of reads mapped to the *L. sinapis* genome (supplementary table S1, Supplementary Material online). Transcriptome assembly and gene expression quantification were performed with STRINGTIE, version 1.3.3 (Pertea et al. 2015), following a standard protocol and the recommended settings for a reference assisted transcriptome assembly (Pertea et al. 2016).

Library scaling factors were computed using a quantile method (Bullard et al. 2010) after including the modifications suggested by Hardcastle et al. (2012). This protocol, previously used in the study of dosage compensation in *Heliconius* butterflies (Walters et al. 2015), is required to accurately estimate normalized expression levels for each gene. Common normalization procedures that rely solely on the number of fragments per kilobase of transcript per million mapped reads (FPKM; Mortazavi et al. 2008) are sensitive to highly expressed genes, making comparisons across libraries unreliable (Dillies et al. 2013). In the original quantile method (Bullard et al. 2010), normalization is carried out by dividing gene counts by the upper quartile of counts for the whole library. When using the modified quantile method (Hardcastle et al. 2012), the normalization factor for each library is taken as the sum of gene expression counts after removing the top 25% most highly expressed genes (after exclusion of zero-count genes). Furthermore, and because expression of Z-linked genes is expected to be asymmetric across sexes and ontogenic stages, normalization is carried out using solely genes assigned to autosomes. The scaling factors thus computed replaced the “per million mapped reads” used in the standard definition of FPKM. Although we will continue to use the term FPKM in the remaining of this article, it should be understood that the values shown have been renormalized using the procedure described above.

After library normalization, expression homogeneity across biological replicates was assessed with pairwise Spearman’s correlation test, as implemented in R, version 3.5.1 (https://cran.r-project.org/; last accessed April 1, 2018). As expression levels were highly correlated across replicates (supplementary fig. S1, Supplementary Material online), groups of samples associated with the same sex and developmental stage were concatenated by calculating a mean FPKM for each gene across biological replicates.

### Chromosomal Assignment and Assessment of Dosage Compensation

RNA-seq reads mapped to the *L. sinapis* draft genome (Talla et al. 2017) were assigned to chromosomes by reciprocal BLAST, version 2.7.1 (Altschul et al. 1990), of *L. sinapis* scaffolds to the *Heliconius melpomene* genome assembly (Heliconius Genome Consortium 2012). This approach rests on a few assumptions that appear to be acceptable: chromosome synteny within Lepidoptera is high (e.g., Ahola et al. 2014), the Z-chromosome is highly conserved across taxa (e.g., Fraïsse et al. 2017) and that *L. sinapis* and *H. melpomene* gene sequences are similar enough for correct orthology identification (Kawahara and Breinholt 2014; Talla et al. 2017). The practice of using homology with reference genomes, with the assumption of synteny, to predict chromosome locations of genes is common in dosage compensation studies where no linkage information from the focal species is available (Harrison et al. 2012; Smith et al. 2014; Gu et al. 2017). It should be noted that *H. melpomene* has a smaller number of chromosomes (*n* = 21) than *L. sinapis* (*n* = 28), the latter being closer to the ancestral Lepidoptera karyotype (*n* = 31; Ahola et al. 2014). This is partly a consequence of autosome fusions in the *H. melpomene* lineage sometime after the split from other Nymphalids (Davey et al. 2016).

*Leptidea* *sinapis* scaffolds with <200 bp mapped to any *H. melpomene* chromosome were presumed to be uninformative and filtered out from further analysis. Scaffolds were assigned as Z-linked or autosomal if more than 95% of the scaffold mapped to either chromosome group (A or Z). This cutoff avoids removing scaffolds where the majority of genes are mapped to one chromosome, whereas only short sequences, not representing complete genes, are mapped to another chromosome. In addition, scaffolds were assigned to individual chromosomes (autosome 1–20 or Z) using the same method but with a less stringent cutoff (>90% scaffold length mapping to a single chromosome). This cutoff was deliberately less restrictive to minimize the loss of data for each respective autosome. In total, 1, 212 *L. sinapis* scaffolds mapped to the *H. melpomene* reference genome. Although this is only 17% of all *L. sinapis* scaffolds (1,212 out of 7,090), this set contains the longest scaffolds with highest contiguity and covers >95% of the *L. sinapis* assembly (Talla et al. 2017). Of the 1,212 mapped scaffolds, 248 were removed based on too short mapping length and the final data used for analysis consisted of 946 scaffolds containing 13,442 genes that were assigned as autosomal and 18 scaffolds containing 405 genes that were assigned as Z-linked. For comparing Z-linked expression to individual autosomes, 677 scaffolds containing 7,617 genes were assigned to specific chromosomes with a >90% mapping cutoff.

Only genes with FPKM > 0 (filtered separately for each sex and developmental stage) were included for dosage compensation analysis. In general, a slightly higher number of Z-linked genes were expressed in males than in females (supplementary table S2, Supplementary Material online). Mean and median autosomal and Z-linked FPKM were calculated separately for each sex in each developmental stage. Median expression levels were then compared between Z-linked and all autosomal genes collectively within each sex (Z/A) and a Mann–Whitney *U* test, as implemented in R, was used to evaluate whether any observed differences were statistically significant. Confidence intervals (95%) for median ratios were obtained by bootstrapping with 10,000 iterations using the R boot package (http://astrostatistics.psu.edu/su07/R/html/boot/html/boot.html, accessed: April 1, 2018). Expression levels of each individual chromosome were compared against all other chromosomes in the genome using the Kruskal–Wallis test followed by Dunn’s test for multiple comparisons as implemented in R. Expression ratios between males and females (**♂/♀**), performed separately for autosomes and Z-chromosomes, were estimated using a similar procedure. In this part of the analysis, only genes with FPKM > 0 in *both* sexes, filtered separately for each developmental stage, were included.

### Analysis of Sex-Biased Expression

A differential gene expression analysis was performed in order to distinguish genes with sex-biased expression. There were two main reasons for this analysis. First, sex-biased genes can be nonrandomly distributed (e.g., Allen et al. 2013) and this may obscure the presence of a global gene regulating mechanism and should therefore be taken into consideration when assessing dosage compensation (Huylmans et al. 2017). Second, sex-specific expression can be informative for characterization of genes and pathways involved in sexual differentiation, sexual conflict, and potentially also for identification of genes involved in initiation and maintenance of dosage compensation. To investigate the presence of sex-biased genes, a differential expression analysis contrasting male and female expression was performed using DESEQ2, version 1.20.0 (Love et al. 2014). The input matrix for DESEQ2 was prepared from the read coverage output from STRINGTIE using the python script “prepDE.py” (https://ccb.jhu.edu/software/stringtie/dl/prepDE.py, accessed: December 1, 2018). Two steps for filtering out lowly expressed genes were taken: First, we filtered out genes expressed in only 1 sample (out of 18), and second, genes with a mean count across biological replicates <1 were also omitted. This filtering strategy removes cases where a single sample has enough counts for the gene in question to pass the filtering for low mean expression, whereas no expression is observed for any other sample (i.e., where true sex-biased expression can be questionable). Detection and removal of batch effects was performed with the R/BIOCONDUCTOR package SVA, version 3.24.4 (Leek et al. 2012). Expected batch effects included family effects, as biological replicates originated from different families were previously shown to have distinct expression profiles (Leal et al. 2018). Furthermore, technical batch effects were also expected to be present as one of the samples was sequenced using a different Illumina machine and flowcell. Finally, differential gene expression analysis was carried out using DESEQ2 with a significance cutoff value (*α*) of 0.05 for the false discovery rate (FDR) correction (Benjamini and Hochberg 1995). Genes were classified as “sex-biased” if their FDR adjusted *P* value was <0.05, the absolute fold change, |log 2 fold change|, was >1, and had a base mean expression across samples >10. A complementary dosage compensation analysis comparing autosomal to Z-linked genes was performed after removing all sex-biased genes from the initial data set. The chromosomal distribution of sex-biased genes was analyzed using a Fisher’s exact test as implemented in R.

### Quartile Analysis of Z-Linked Gene Expression

An additional way to investigate the presence of a global gene regulating mechanism is to analyze Z-linked genes across different magnitudes of expression levels (Harrison et al. 2012). The rationale behind this is that dosage effects should be most prominent for highly expressed genes because the single Z-chromosome in females should reach transcriptional saturation before the two Z-chromosomes in males. For lowly expressed genes, there should be enough headroom in the transcriptional machinery to allow for an equal expression between the sexes without any additional regulation (gene dose should be less important). Therefore, if a global regulating mechanism is present, the difference between male and female Z-linked expression levels should be equal across all levels of comparison (Harrison et al. 2012; Walters et al. 2015). For each sex and developmental stage, Z-linked genes were divided into four quartiles based on their FPKM expression value (Walters et al. 2015). The results were visualized with boxplots and the differences between male and female expression distributions within each quartile were tested using Mann–Whitney *U* tests as implemented in R.

### Analysis Based on De Novo Transcriptome Assembly

As a complement to the analysis described above, a de novo transcriptome was assembled based on the same set of Illumina libraries. This step is recommended as dosage compensation mechanisms may vary from species to species within Lepidoptera. By relying solely on the mapping of *L. sinapis* contigs to the *H. melpomene* genome, there was a risk of failing to detect genes active in *L. sinapis* that are not present—or have not yet been identified—in *Heliconius*. The de novo transcriptome was assembled using TRINITY, version 2.3.2 (Grabherr et al. 2011). KALLISTO, version 0.43.0 (Bray et al. 2016) was used for mapping and quantification of transcript abundance. In silico functional annotation of the transcriptome was based on the TRINOTATE suite, version 3.0.2 (https://trinotate.github.io/, accessed: December 1, 2018) with *Drosophilamelanogaster* and three lepidopteran species (*B. mori*, *Danaus plexippus*, and *P. machaon*) as references. A detailed description of the pipelines and scripts used can be found in Leal et al. (2018). Removal of batch effects and identification of sex-biased genes was carried out using SVA and DESEQ2, respectively, as described above.

### Gene Ontology Overrepresentation Test

To find biological processes associated with sex-biased genes identified above, a gene ontology (GO) term enrichment analysis was performed (Ashburner et al. 2000). The primary objective was to investigate if there were specific processes that could be linked to initiation of dosage compensation, but the results from the GO analysis should also corroborate the differential gene expression analysis by showing enrichment of sex-specific genes. In addition, the analysis might also reveal functions related to sexual differentiation and dimorphism that could be subject for further studies. Sex-biased genes of each sex and developmental stage, identified on the basis of the de novo transcriptome, were tested separately using TOPGO, version 2.28.0 (https://www.bioconductor.org/packages/release/bioc/html/, accessed: December 1, 2018).

## Results

### Reduced Median Expression of Z-Linked Genes

**Fig. 1.**
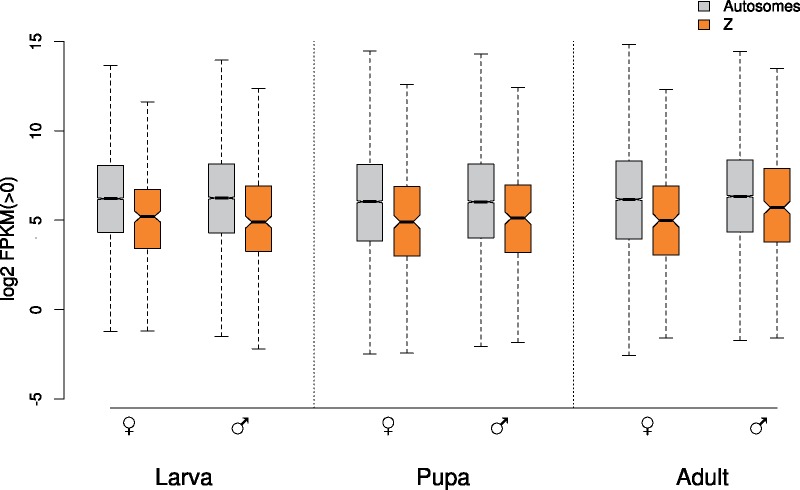
—Log 2 transformed FPKM distributions, representing autosomal (gray) and Z-linked (orange) gene expression observed in females and males during larva, pupa, and adult developmental stages. Zero-expression genes were removed separately for males and females. Boxes represent the interquartile range and whiskers extend to 1.5× the interquartile range. Notches represent the 95% confidence interval of median (black bar) expression and outliers have been removed. MWU test for differences in expression levels between autosomal and Z-chromosome linked genes was significant (*P* value < 0.05) for all pairwise comparisons (supplementary table S3*A*, Supplementary Material online).

The median expression level of Z-linked genes was significantly reduced as compared with autosomal genes across all three developmental stages in both males and females (fig. 1 and table 1). The relative expression of Z-linked genes as compared with autosomal genes, was <1 in all stages, and ranged from 0.39 (largest difference) in male larvae, to 0.65 (smallest difference) in male adults (table 1 and supplementary table S3*A*, Supplementary Material online). The median expression level of autosomal genes was reduced in pupae, coinciding with a decrease in the number of active genes during this stage (table 1 and supplementary table S2, Supplementary Material online). Median Z-linked expression increased progressively with each developmental stage in males, whereas in females it was highest in instar-V larvae. This correlates with the gradual increase in the number of transcribed Z-linked genes in males as ontogeny proceeds, whereas in females the opposite trend was observed (supplementary table S2, Supplementary Material online).

**Table 1:** (*A*) Median and Mean Expression Levels for Autosomal (A) and Z-Linked (Z) Genes Observed in Females and Males, for Different Developmental Stages

(*A*)	Median FPKM				Mean FPKM			
	Female		Male		Female		Male	
Stage	A	Z	A	Z	A	Z	A	Z
Larva	74	37	76	30	4,854	934	3,785	759
Pupa	66	30	65	35	2,757	795	5,436	595
Adult	72	32	81	52	2,006	590	2,134	1,045


(*B*)	Z:A Median Ratio		M:F Median Ratio	
Stage	Female	Male	Autosomal	Z-Linked
Larva	0.50 (0.42–0.60)***	0.39 (0.25–0.48)***	1.08 (1.05–1.12)	1.03 (0.74–1.24)
Pupa	0.46 (0.32–0.58)***	0.53 (0.30–0.71)***	0.92 (0.89–0.96)	1.09 (0.79–1.37)
Adult	0.44 (0.26–0.57)***	0.65 (0.47–0.78)**	1.11 (1.06–1.15)***	1.67 (1.12–2.03)***


Note.—Zero-expression genes removed separately for males and females. (*B*) Summary of median gene expression ratios for Z-chromosome to autosome (Z:A) and male to female (M:F) comparisons, observed for different developmental stages. Only genes with FPKM > 0 in both sexes were included when computing M:F ratios. Ninety-five percent bootstrap confidence intervals shown in parentheses. Significance levels for differences in expression levels of female versus male and autosomal versus Z-chromosome linked genes are indicated (*P≤0.05, **P≤0.01, and ***P≤0.001). Exact *P* values for each comparison are given in supplementary table S3*A*, Supplementary Material online.

Comparison of median expression levels between males and females, which included only genes expressed in both cohorts, revealed no significant difference between the two sexes during the larval and pupal stages, either in autosomal or in Z-linked expression (table 1). In adults, the Z-linked median expression in males was 67% higher than in females, although still lower than the level observed for autosomes. A detailed analysis of male versus female gene expression across different expression ranges revealed a high degree of correlation between the two sexes, in particular during the larval stage (supplementary fig. S2, Supplementary Material online). The number of asymmetrically expressed genes increased during the pupal and adult stages, whereas the distribution of sex-biased genes was not uniform. More specifically, the regression analyses showed that weakly expressed genes were generally predominant in females—in particular autosomal genes—whereas highly expressed genes were slightly shifted toward higher expression in males. Autosomal and the Z-linked regression lines were generally overlapping, with an exception in adults, where Z-linked expression was male-biased in comparison to autosomal expression (supplementary fig. S2, Supplementary Material online).

**Fig. 2.**
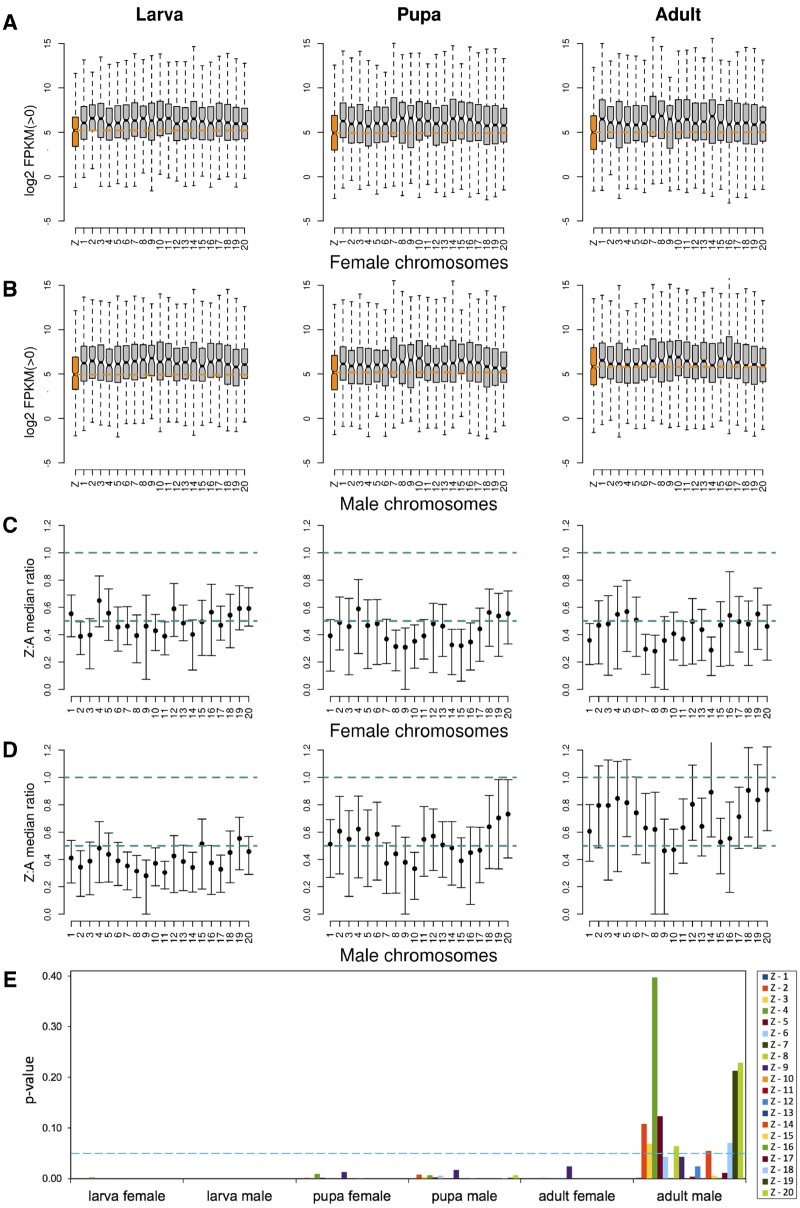
—Log 2 FPKM distributions showing gene expression levels for individual chromosomes in (*A*) females and (*B*) males. Zero-expression genes were removed separately for males and females. Dashed orange lines mark the Z-linked median expression level. Boxes represent the interquartile range and whiskers extend to 1.5× the interquartile range. Notches represent the 95% confidence interval of median (black bar) expression. Outliers were removed. Total number of genes in each stage and for each sex is shown in supplementary table S2, Supplementary Material online. (*C*, *D*) Median gene expression ratios for Z-chromosome to individual autosomes (Z:A), observed in females and males, respectively, for different developmental stages. Error bars correspond to 95% bootstrap confidence intervals. Dashed lines indicate Z:A ratios equal to 0.5 and 1. (*E*) Results from Dunn’s test (*P* values), following significant Kruskal–Wallis *H* test (*P* value < 0.001), comparing gene expression levels between the Z-chromosome and each individual autosome. *P* values above the 0.05 threshold (dashed line) indicate chromosome pairs with expression distributions that were not significantly different. Analyses were carried out separately for each sex and developmental stage.

To get a more detailed picture of expression differences between autosomal and Z-linked genes, we also split the autosomal gene expression data into individual autosomes (inferred from *H. melpomene*) (fig. 2). For most sex/stage combinations, this analysis confirmed the global patterns observed when all autosomes were evaluated jointly. The median expression of Z-linked genes in females was lower than in individual autosomes in all developmental stages (fig. 2*A*). A similar pattern was observed in males during the two initial developmental stages (fig. 2*B*). By contrast, in adults, Z-expression in males increased noticeably, being on par with that of some of the autosomes. In fact, although the expression level of autosomal genes was generally uniform, expression level distributions often differed significantly between individual chromosomes (Kruskal–Wallis *H* test, *P* value < 0.001; fig. 2). We therefore assessed variation across chromosomes by comparing the Z-chromosome to all individual autosomes one by one. This analysis showed that the distributions of expression levels between the Z-chromosome and individual autosomes were significantly different in all stages in females (fig. 2*C* and *E* and supplementary fig. S3 and supplementary table S4, Supplementary Material online). In adult males, Z-linked genes had expression levels similar to those observed in 9 out of 20 autosomes (fig. 2*D* and *E*, and supplementary fig. S3 and supplementary table S4, Supplementary Material online). Taken together, these results suggest that the low Z:autosome ratio in median expression levels observed in adult males masks a more nuanced picture where the Z-chromosome is in expression equilibrium with a large number of autosomes.

### Increased Sex-Biased Expression with Each Developmental Stage

An initial assessment of sex-biased gene content was done by contrasting and visualizing expression ratio distributions between males and females, for each individual stage and separately for autosomal and Z-linked genes. The expression density distributions were narrowest in larvae, indicating a comparatively homogenous expression pattern between males and females for both autosomal and Z-linked genes (supplementary fig. S4, Supplementary Material online). In pupae and adults, the variance increased substantially, suggesting a successive increase in sex-biased expression. In adults, the Z-chromosome expression ratios were significantly skewed toward higher expression in males compared with autosomal ratios (Mann–Whitney *U* test, *P* value = 1.0 × 10^−8^), indicating a larger fraction of male-biased genes.

**Fig. 3.**
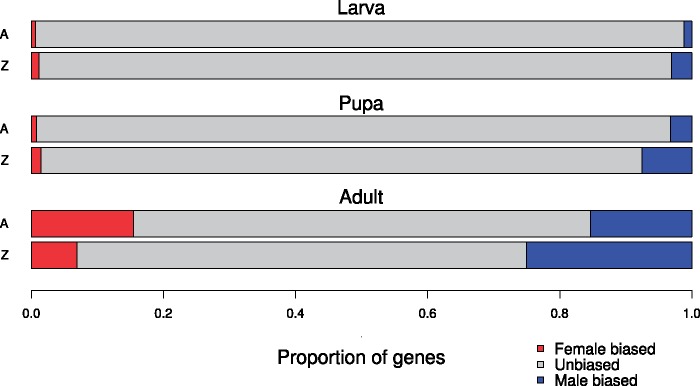
—Proportions of autosomal and Z-linked genes with sex-biased expression computed separately for each developmental stage. The gene counts for each respective class are presented in supplementary table S6, Supplementary Material online.

To further quantify sex-biased genes, a differential expression analysis was performed. Before running the analysis, batch effects were detected and removed. The male to female expression differentiation analysis confirmed that the number of genes with sex-biased expression increased across developmental stages (fig. 3 and supplementary fig. S5 and supplementary table S5, Supplementary Material online). After filtering, there were in total 207 sex-biased genes in larvae, 479 in pupae, and 3,778 in adults. In larvae and pupae, male-biased genes were significantly overrepresented, whereas sex-biased genes were divided more equally between the sexes in adults (supplementary table S5, Supplementary Material online).

### Enrichment of Male-Biased Z-Linked Genes

The presence of sex-biased genes can affect Z-chromosome to autosome expression ratios, thus masking the potential presence of dosage compensation. To assess whether observed genomic distributions of sex-biased genes—autosomal or Z-linked—departed from a random chromosome distribution, we compared the sex-biased genes to the global gene distribution (not sex biased) on autosomes and the Z-chromosome. In female larvae and pupae, the distribution of sex-biased genes did not deviate significantly from expected under a random distribution model (Fisher’s exact test, *P* value > 0.05; supplementary table S6, Supplementary Material online). Female adults, on the other hand, showed a significant enrichment of autosomal female-biased genes (*P* value = 6.5 × 10^−5^, supplementary table S6, Supplementary Material online, and fig. 3), suggesting that genes overexpressed in females tend to be located in autosomes. This contrasted with males, where male-biased genes were considerably overrepresented on the Z-chromosome in all three developmental stages (*P* value < 0.05, supplementary table S6, Supplementary Material online, and fig. 3).

### Z-Linked Expression between Males and Females Converges after Controlling for Sex-Biased Genes

**Fig. 4.**
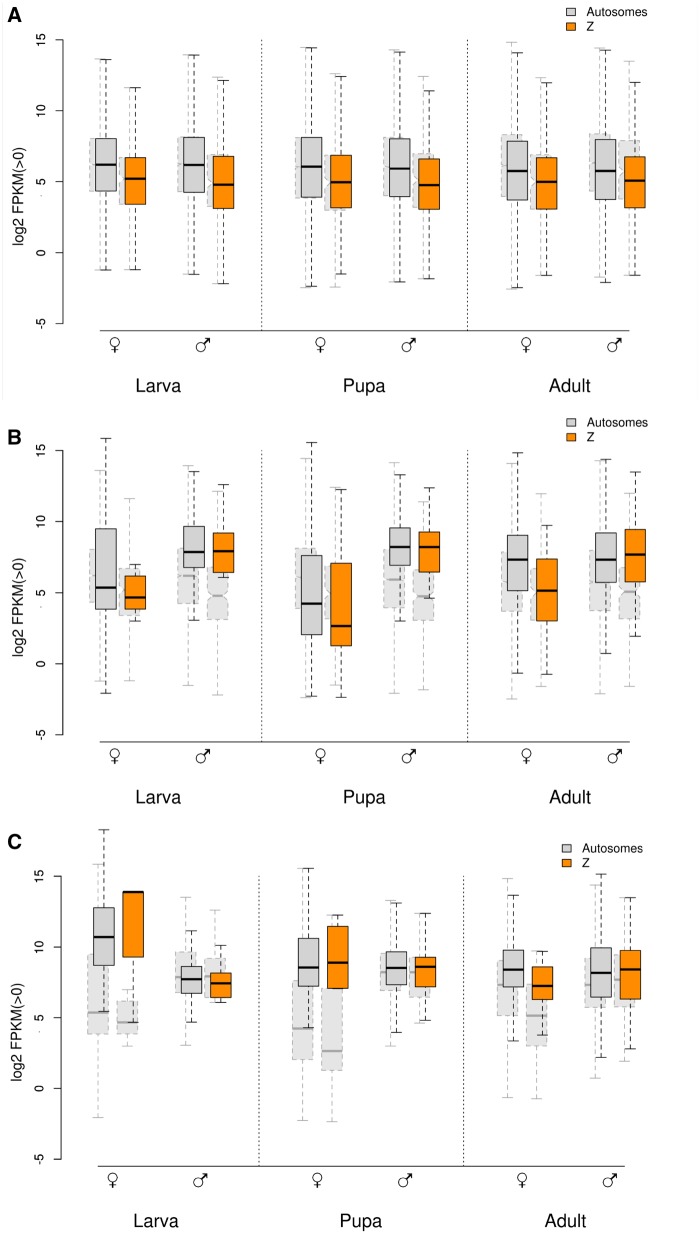
—(*A*) Log 2 FPKM distributions, comparing before (light-gray, shadowed boxes with dashed borders) and after (solid border boxes) removing sex-biased genes in larvae, pupae, and adults. The MWU test for differences in expression levels between autosomal and Z-linked genes was significant (*P* value < 0.05) for all pairwise comparisons (supplementary table S7*A*, Supplementary Material online). (*B*) Log 2 FPKM distribution of sex-biased genes in larvae, pupae, and adults (solid border boxes). Distribution of non-sex-biased genes shown in background (light-gray, shadowed boxes with dashed borders). MWU test for sex-biased genes between Z and autosomal chromosomes yielded no significant difference for all comparisons (*P* value > 0.38), with the exception of adult females (*P* value = 3 × 10^−7^). (*C*) Log 2 FPKM distribution of genes upregulated in each sex (vis-a-vis the other sex) in larvae, pupae, and adults (solid border boxes). Distribution of sex-biased genes (both up- and down-regulated) shown in background (light-gray, shadowed boxes with dashed borders). Zero-expression genes were removed separately for males and females. Boxes represent the interquartile ranges and whiskers extend to 1.5× the interquartile range. Median values indicated by black bar. Outliers were removed from the plot.

After removing sex-biased genes from the data set and reanalyzing patterns of dosage compensation, we found that the difference between the overall expression of male and female Z-linked genes was further reduced (table 2, fig. 4*A*, and supplementary table S7*A*, Supplementary Material online). Convergence between the two sexes was most pronounced in the adult stage, where Z-linked sex-biased genes were significantly more numerous than in larvae and pupae, predominantly an effect of overexpression in males (supplementary table S6, Supplementary Material online). A small disparity between adult males and females in both autosomal and Z-linked expression remained after controlling for sex-biased genes, although the difference was not statistically significant for the latter (table 2).

**Table 2:** (*A*) Median and Mean Expression Levels for Autosomal (A) and Z-Linked (Z) Genes after Excluding Sex-Biased Genes, Observed in Females and Males, for Different Developmental Stages

(*A*)	Median FPKM				Mean FPKM			
	Female		Male		Female		Male	
Stage	A	Z	A	Z	A	Z	A	Z
Larva	74	37	73	28	4,289	858	3,724	744
Pupa	67	31	61	27	2,729	805	5,581	583
Adult	54	32	54	34	1,171	745	1,564	1,137


(*B*)	Z:A Median Ratio		M:F Median Ratio	
Stage	Female	Male	Autosomal	Z-Linked
Larva	0.50 (0.41–0.59)***	0.38 (0.26–0.46)***	1.04 (1.01–1.07)	0.93 (0.70–1.10)
Pupa	0.46 (0.32–0.58)***	0.44 (0.23–0.59)***	0.87 (0.84–0.91)***	1.01 (0.80–1.25)
Adult	0.59 (0.35–0.77)***	0.62 (0.45–0.79)***	1.20 (1.15–1.25)***	1.29 (0.90–1.51)

Note.—Zero-expression genes removed separately for males and females. (*B*) Median expression level ratios for Z-chromosome to autosome (Z:A) and male to female (M:F) comparisons, observed after removing sex-biased genes. Only genes with FPKM > 0 in both sexes were included when computing M:F ratios. Ninety-five percent bootstrap confidence intervals shown in parentheses. Significance levels for differences in expression levels of female versus male and autosomal versus Z-chromosome linked genes are indicated (*P≤0.05, **P≤0.01, and ***P≤0.001). Exact *P* values for each comparison are given in supplementary table S7, Supplementary Material online.

**Fig. 5.**
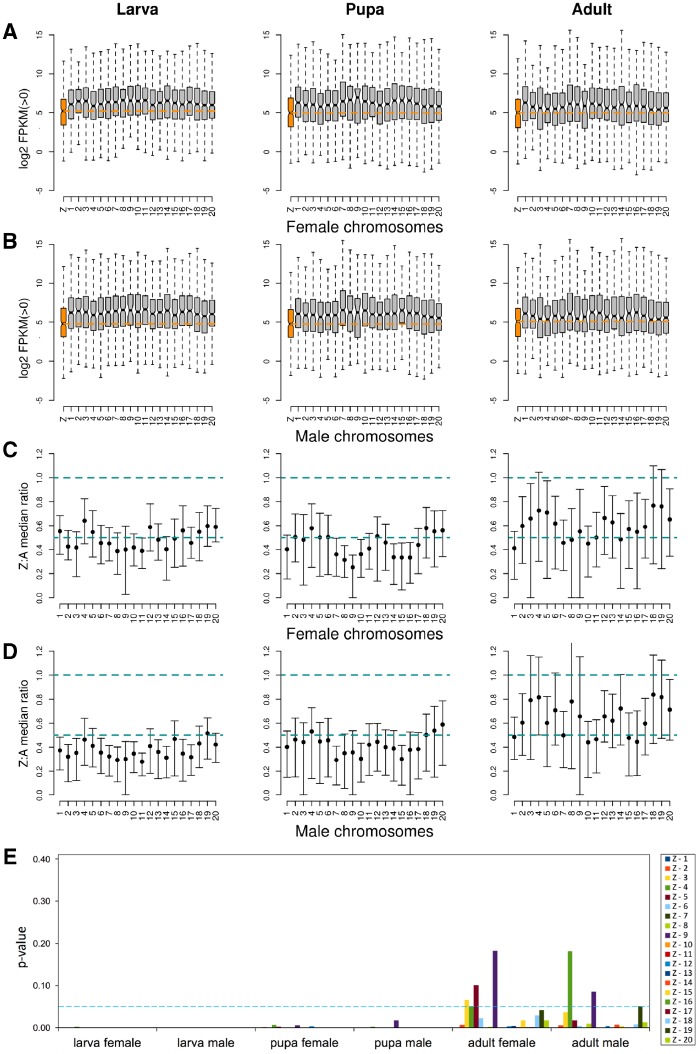
—Log 2 FPKM distributions showing gene expression levels for individual chromosomes in (*A*) females and (*B*) males, after removing sex-biased genes. Zero-expression genes were removed separately for males and females. Dashed orange lines mark the Z-linked median expression level. Boxes represent the interquartile range and whiskers extend to 1.5× the interquartile range. Notches represent the 95% confidence interval of median (black bar) expression. Outliers were removed. (*C*, *D*) Median gene expression ratios for Z-chromosome to individual autosomes (Z:A), observed in females and males, respectively, for different developmental stages and after removing sex-biased genes. Error bars correspond to 95% bootstrap confidence intervals. Dashed lines indicate Z:A ratios equal to 0.5 and 1. (*E*) Results from Dunn’s test (*P* values), following significant Kruskal–Wallis *H* test (*P* value < 0.012), comparing gene expression levels between the Z-chromosome and each individual autosome. *P* values above the 0.05 threshold (dashed blue line) indicate chromosome pairs with expression distributions that were not significantly different. Analyses were carried out separately for each sex and developmental stage.

Importantly, the ratio of Z-linked to autosomal gene expression in adult females increased after correcting for sex-biased genes, now matching the value observed in males (table 2). The distributions of pairwise comparisons between the Z-chromosome and individual autosomes, highly discordant between adult males and females prior to removing sex-biased genes (fig. 2), also became more alike once the latter were removed from the analysis (fig. 5). There was however a subtle difference between the two sexes on how the gap in expression levels between the Z-chromosome and autosomes was reduced during the adult stage. In males, this was attained by upregulated expression of genes located on the Z-chromosome: Median gene expression of all Z-linked genes, both biased and unbiased (FPKM = 52, table 1), matched those of unbiased autosomal genes (FPKM = 54, table 2 and fig. 4*A*). Additionally, when comparing sex-biased genes associated with either autosomes or the Z-chromosome, we observed a complete A:Z dosage compensation in adult males, a pattern that was consistent across all developmental stages (fig. 4*B*).

In females, median Z-linked expression remained relatively constant across all three developmental stages (pairwise MWU test, *P* value > 0.4; table 1 and supplementary table S3*B*, Supplementary Material online), not changing noticeably even after removal of sex-biased genes (table 2 and supplementary table S7*B* and *C*, Supplementary Material online). In contrast, female expression of non-sex-biased autosomal genes dropped considerably in adults, edging closer to the levels of Z-linked genes (table 2 and supplementary table S7*A* and *C*, Supplementary Material online). This is expected as more sex-biased genes were removed during the adult stage, a trend observed also in males (table 2). In this analysis, zero-expression genes were removed separately for each sex, and the result was complete male to female dosage compensation in adults, both among autosomes and Z-chromosomes, although slightly different gene sets were active in each sex. When expression values for exactly the same gene set (genes with nonzero FPKM in both sexes) were analyzed, autosomal expression in females was equal to or higher than in males in larvae and pupae but decreased considerably in adults (supplementary table S8, Supplementary Material online, and table 2*B*). In adults, there was 15–20% reduced autosomal expression in females compared with males (for genes with FPKM > 0 in both sexes, *P* < 0.05, table 2*B* and supplementary table S7*A*, Supplementary Material online). In contrast to males, there was no parity in expression between autosomal and Z-linked genes in adult females when the analysis was restricted to sex-biased genes (fig. 4*B*). Instead, this set of autosomal genes were matched in expression only by the subset of Z-linked genes upregulated in adult females (MWU test, *P* value > 0.9; fig. 4*C*). Expression for Z-linked sex-biased genes (including both up- and down-regulated genes) was not significantly different from expression values observed in unbiased autosomal genes (MWU test, *P* value > 0.05; fig. 4*B*).

### Quartile Analysis of Z-Linked Expression

To further investigate if Z-linked genes were subject to a global gene regulating mechanism, Z-linked expression was compared between males and females at different levels of magnitude by separating genes into four quartiles based on the maximum expression level of a specific gene in either male or female. With one exception (larva, second quartile), no significant differences in Z-linked expression were observed between males and females during the larval and pupal stages (supplementary fig. 6*A* and supplementary table S9, Supplementary Material online). Conversely, there was a significantly higher expression in adult males across all four quartiles. Given that ∼20% of all Z-linked genes had male-biased expression in the adult stage, there is a substantial risk that this could affect the quartile analysis. After excluding sex-biased genes, the differences previously observed in adults became less pronounced, in particular for genes with either extremely high or extremely low expression (supplementary fig. 6*B* and supplementary table S10, Supplementary Material online). However, expression levels were male-biased for intermediate values, suggesting that the gap between sexes observed for all genes aggregated (table 2*B*) is genuine. Although these results cannot rule out a chromosome-wide regulation of Z-linked genes per se, they do suggest some nuances in regulation at different levels of expression.

### Functional Differences between Male and Female Sex-Biased Genes

Functional analysis of sex-biased genes was based on the de novo transcriptome assembly, as this approach allows for complete analysis of all genes expressed in *L. sinapis*, as opposed to only those that map to the *H. melpomene* genome. Differential gene expression analysis of the de novo transcriptome confirmed the surge in the number of sex-biased genes with development, increasing from 203 in larvae to 4,965 in adults (supplementary table S11, Supplementary Material online). Of the 164 male-biased genes identified in larvae, 130 (79%) remained upregulated in males also in pupae and adults (supplementary table S12, Supplementary Material online), suggesting that some core biological functions required for male viability and/or fertility are conserved throughout ontogenetic shifts. In females, the functions of sex-biased genes varied considerably across ontogenetic stages. One of the four genes found to remain upregulated in females during all three stages is a homolog of the *B. mori VG* (vitellogenin) gene, which codes for a key egg-yolk protein precursor synthesized extraovarially by female insects (Engelmann 1979). Of the remaining three genes, two have also been found in *P. machaon*, *RR48_05572* and *RR48_00002*, but the functions remain to be determined. The fourth gene listed was not annotated by TRINOTATE and blast searches yielded no significant hits.

In order to gain a better understanding of the biological functions of the sex-biased genes, GO term analyses were performed separately for each sex and developmental stage. The top GO categories (lowest *P* value) enriched in males relate to the synthesis of male germ cells and remained rather constant across development (terms highlighted in blue in supplementary figs. S7*A*, S8*A*, and S9*A*, Supplementary Material online). This list includes “motile cilium assembly” (GO:0044458), “cilium movement” (GO:0003341), and “flagellated sperm motility” (GO:0030317). Enriched GO terms associated with female sex-biased genes lacked a clear common denominator. In larvae and pupae, most terms were associated with metabolic and biosynthetic processes (supplementary figs. S7*B* and S8*B*, Supplementary Material online). There was a major increase in the number of female-biased genes from pupae (115 genes) to adults (2,312 genes) (supplementary table S11, Supplementary Material online). The top 35 GO categories enriched in adult females were associated with ribosomal assembly, RNA processing, and cytoplasmic translation (terms highlighted in pink in supplementary fig. S9*B*, Supplementary Material online). Histone acetylation (GO:0016573) and phosphorylation (GO:0016572), modifications frequently associated with enhanced levels of transcription (Grunstein 1997; Strahl and Allis 2000), also showed up among the list of significant GO terms. The large number of genes underpinning these categories (e.g., there were 90 highly significant genes among the 109 annotated for “cytoplasmic translation” [GO:0002181]) provide strong support for increased levels of translation in adult females. The full list of overrepresented GO terms for each sex and developmental stage is provided in supplementary table S13, Supplementary Material online.

### Molecular Mechanisms of Dosage Regulation

In addition to the GO analysis, we attempted to identify orthologs to previously characterized genes involved in dosage compensation in the Lepidoptera model species, *B. mori*. However, homology searches in the *L. sinapis* genome and transcriptome assemblies using the genes *MASC* and *FEM*, known to be involved in sex determination and dosage compensation in *B. mori* (Kiuchi et al. 2014), did not result in any significant hits.

**Fig. 6.**
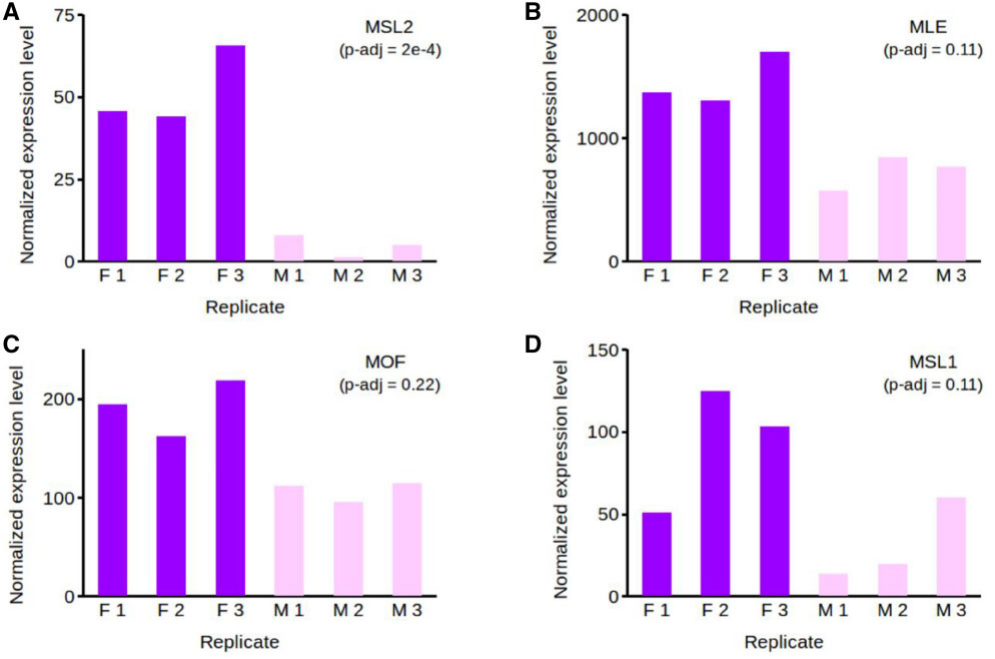
—Normalized expression values for genes *MSL2* (*A*), *MLE* (*B*), *MOF* (*C*), and *MSL1* (*D*) in adults. Counts have been adjusted for the presence of batch effects. Expression values are shown separately for each female (F1–F3, violet) and male (M1–M3, light pink) replicate. Data based on the de novo transcriptome assembly.

The most well-known chromosome-wide mechanism of dosage compensation in insects is the MSL complex observed in *Drosophila*. It binds to the X-chromosome in males, potentially initiating hyperactivation of X-linked genes (e.g., Kuroda et al. 2016). The MSL complex contains five proteins, *MSL1*, *MSL2*, *MSL3*, *MLE* (maleless), and *MOF* (males-absent on the first), as well as two noncoding RNAs, *roX1* and *roX1* (male-specific RNAs on the X) (Conrad and Akhtar 2012). *MSL2* is the focal gene in the MSL complex, crucial for its assembly (Conrad and Akhtar 2012). We investigated whether the five protein coding MSL genes were present in the *L. sinapis* transcriptome and whether they were upregulated in females. We found homologs for all five genes in the transcriptome and *MSL1*, *MSL2*, *MLE*, and *MOF* were all upregulated in adult females, whereas expression levels for the *MSL2* gene were very low in males (fig. 6 and supplementary tables S14 and S15, Supplementary Material online). We also detected the presence of two dosage compensation modulators, *TOPO2* (DNA topoisomerase 2) and *LOQS* (loquacious), upregulated in adult females (supplementary table S14, Supplementary Material online). *TOPO2* has been shown to associate with the MSL complex and to be required for proper dosage compensation in *Drosophila* (Cugusi et al. 2013). *LOQS* encodes an enzyme involved in the biogenesis of small interfering RNAs (siRNA) implicated in the binding and selective recognition of X chromatin by the MSL complex in *Drosophila* (Menon and Meller 2012). Further investigation of these genes revealed that expression differentials between females and males were minimal during the larval and pupal stage, becoming pronounced only in adult specimens (supplementary fig. S10, Supplementary Material online). Differences in expression for the *MSL3* gene were not statistically significant in any of the three ontogenetic stages.

## Discussion

### General

In Lepidoptera, patterns of dosage compensation have been studied in detail in a few moth and butterfly families (Harrison et al. 2012; Smith et al. 2014; Walters et al. 2015; Gopinath et al. 2017; Gu and Walters 2017; Gu et al. 2017; Huylmans et al. 2017; Catalan et al. 2018), but we still lack knowledge about how regulatory mechanisms are tuned separately in males and females to attain intra- and inter-sexual dosage equalization. In this study, RNA-seq data were used to characterize and quantify patterns of dosage compensation and sex-biased gene expression in the wood white (*L. sinapis*) butterfly, spearheading this type of analysis in the large lepidopteran family Pieridae. In addition, most previous studies investigating patterns of dosage compensation in Lepidoptera have focused on adult samples. Here, we assessed how dosage compensation and sex-biased expression varied across developmental stages to elucidate the regulatory dynamics of gene dosage between sexes and chromosome classes.

### Reflections on Models of Dosage Compensation in Lepidoptera

Only a decade ago, dosage compensation was thought to be absent in lepidopteran insects (Traut et al. 2007). Recent studies in Lepidoptera suggest that male Z-linked gene expression in many cases is reduced to the level observed in females (Walters et al. 2015; Gopinath et al. 2017; Gu et al. 2017; Huylmans et al. 2017; Catalan et al. 2018), a pattern generally referred to as incomplete dosage compensation (Gu and Walters 2017). A major question arising from these observations is why there is downregulation in males, resulting in Z-chromosome to autosome imbalance, instead of Z-chromosome upregulation in females? Most male-heterogametic (XY) lineages have either complete or incomplete dosage compensation (Gu and Walters 2017). The same is true for Lepidoptera, but not for other female-heterogametic lineages (ZW) like birds and snakes who seem to lack a general dosage compensation mechanism (Mank 2013; Gu and Walters 2017). Taken together, these observations suggest that intersexual expression balance is in general more important than expression parity between autosomes and sex-chromosomes. There is now ample evidence that distinct sex-chromosome systems can result in different forms of dosage regulating mechanisms, and it is likely that other lineage specific genetic properties influence the process. This is to some degree in conflict with the classical theory of dosage compensation, which puts emphasis on the importance of maintaining expression of sex-linked genes at the “ancestral” level (Ohno 1967). Lepidoptera are unique as compared with other female-heterogametic taxa in that they lack meiotic recombination (achiasmy) in females (e.g., Turner and Sheppard 1975). Lack of recombination is expected to accelerate the degradation of the sex-limited chromosome, W (Wright et al. 2016), and the rate of the process could theoretically restrict mechanisms for dosage compensation. It is still uncertain if the W-chromosome is ancestral to all lepidopteran lineages (W could potentially have been lost already in the common ancestor of Lepidoptera and Trichoptera) or if it was acquired secondarily (Traut et al. 2007; Fraïsse et al. 2017). In female-heterogametic lineages with meiotic recombination in both sexes, degradation of the W-chromosome occurred successively via inversions leading to different evolutionary strata with increasing degrees of differentiation between the two sex-chromosomes (Handley et al. 2004; Vicoso et al. 2013). The slower overall degradation in these systems, combined with recurrent recombination between the Z-chromosome and the W-chromosome in linear regions, could have enabled a gradual upregulation of dose sensitive genes in females in a way not feasible in the achiasmatic Lepidoptera. However, it is not known in which succession achiasmy, dosage compensation and the W-chromosome degradation evolved in Lepidoptera, a knowledge that would be essential in order to understand potential causal relationships.

### Masculinization of the Z-Chromosome

We quantified the degree of sex-bias in expression across developmental stages and the genomic distribution of sex-biased genes. Our analysis revealed a successive increase in sex-biased genes with each developmental stage, in particular for male-biased genes. This is in line with previous analyses in chicken (*Gallus gallus*) (Mank et al. 2010) and the silk moth (*B. mori*) (Zhao et al. 2011), other taxa with female heterogamety. There was a nonrandom genomic distribution of male-biased genes in all three developmental stages, with a significantly higher proportion located on the Z-chromosome than on the autosomes. Conversely, in adults, there was a higher proportion of genes with female-biased expression located on autosomes. Masculinization of the Z-chromosome appears to be common in lepidopteran species (Arunkumar et al. 2009; Sackton et al. 2014; Walters et al. 2015; Gu et al. 2017; Huylmans et al. 2017), a feature that may have favored evolution of a general downregulation of Z-linked genes in males, instead of upregulation in females. As a consequence of inherent dose differences for Z-linked genes between males and females, and the direct inheritance of the Z-chromosome from sires to male offspring, enrichment of male-beneficial genes on the Z-chromosome could be selectively advantageous (Reeve and Pfennig 2003; Kaiser and Bachtrog 2010). This sexually antagonistic enrichment of male-biased genes on the Z-chromosome would be a restrictive force against a general upregulation of Z-linked genes in females. In addition, given that intersexual balance appears to be important, and that hyperexpression in the heterogametic sex could have detrimental effects (Veitia and Potier 2015), a general upregulation of Z-linked genes could be consequently disadvantageous in this system. Indeed, upregulation of Z-linked genes in both sexes has never been observed unless it is combined with male Z-chromosome inactivation or a general downregulation of the X as seen in XY-systems (Gu and Walters 2017).

From the female perspective, it should obviously be beneficial to maintain low expression of all male-biased genes. The sexual conflict generated by enrichment of male-beneficial genes on the Z-chromosome could be counteracted by a mechanism resulting in a general downregulating of the Z-chromosome, but allowing for dosage compensation escape of genes with male-beneficial functions. Taken together, this hypothetical scenario would result in a normal (to high) expression of male-beneficial genes in males, low expression of female-detrimental genes in females, and an overall balance between sexes for genes without sex-specific fitness effects. Masculinization of the Z-chromosome has also been observed in systems lacking a general dosage compensation mechanism, such as birds (e.g., Mank 2013). In contrast, *M. sexta* and *P. machaon*—the only two lepidopteran species so far showing evidence for complete dosage compensation—did not show enrichment of male-biased genes on the Z-chromosome (Smith et al. 2014; Huylmans et al. 2017). This suggests that specific dosage compensation mechanisms could restrict enrichment of sex-biased genes on the Z-chromosome or vice versa. The generality of this pattern obviously has to be explored further. Initially this could be done by characterizing dosage compensation and genomic distributions of sex-biased genes in additional lepidopteran species representing a wider taxonomic sampling of extant families.

The ontology enrichment analysis revealed that functions related to male fertility were consistently male-biased over developmental stages, whereas functional classes of female-biased genes varied between stages. The functional conservation in males stays in some contrast to what has previously been observed in chicken (Mank et al. 2010). However, the analysis in chicken was based on autosomal genes only and it is therefore unclear if the inconsistency really reflects a biological difference between birds and butterflies.

### Multilayered Tuning of Dosage Compensation in Adult Wood Whites

Our analyses generally confirm previously observed patterns of dosage compensation in Lepidoptera but add a few important nuances. Once sex-biased genes were excluded, the sex difference in Z-linked expression was reduced. This agrees with results obtained previously for *P. interpunctella* (Huylmans et al. 2017) and highlights the substantial impact of sex-biased genes on the average gene expression level and the importance of taking this potential bias into account when assessing dosage compensation. However, the convergence in expression was not complete for genes with intermediate expression levels. A weak male-bias in Z-chromosome expression has also been observed in *Heliconius* butterflies (Walters et al. 2015; Catalan et al. 2018). When comparing Z-chromosome to autosome expression at the aggregate level, a statistically significant difference was observed in *L. sinapis*, even after removing sex-biased genes, again corroborating observations made earlier in other lepidopteran systems. A more detailed analysis of expression patterns, however, revealed that Z-linked expression in adult wood whites matches that of autosomes when genes are broken down into separate categories (supplementary fig. S11*A*, Supplementary Material online). In adult males, pairwise comparison between Z-linked expression and that of individual autosomes showed that nine autosomes had expression distributions similar to the one associated with the Z-chromosome. In addition, there was complete dosage compensation between Z-linked genes (including both unbiased and sex-biased genes) and unbiased autosomal genes. The expression distribution of sex-biased autosomal genes was also fully matched by that of sex-biased Z-linked genes. These results suggest that autosome to Z-chromosome tuning in adult males is multilayered, with expression of different categories of Z-linked genes being regulated to match that of distinct classes of autosomal genes, and that wood whites have followed an evolutionary trajectory that kept a balance between Z chromosomal expression in adult males (the homogametic sex) and autosomal genes whose expression is sex neutral. Genes whose expression diverged between sexes, on the other hand, also evolved in concert in autosomes and in the Z-chromosome, to preserve the proper stoichiometry of gene products.

The mechanism for dosage compensation between autosomes and the Z-chromosome in adult female wood whites involved a different range of gene categories (supplementary fig. S11*A*, Supplementary Material online). Expression values for unbiased autosomal genes were in general higher than those observed in Z-linked genes. However, the gap in expression was substantially narrower in adults than in larvae and pupae, and the expression levels of sex-biased Z-linked genes (both upregulated and downregulated) were not significantly different from unbiased autosomal genes. Autosomal sex-biased genes were in turn matched in expression by Z-linked genes upregulated in females. A possible interpretation of these results is that, as sex-chromosomes differentiated, 1) Z-chromosome to autosome dosage in the heterogametic sex was tuned by lowering autosomal expression to keep it within range of Z-chromosome expression and 2) equalization in females has been constrained to a narrower group of gene categories (vis-a-vis males), in part because of sexually antagonistic enrichment of male-biased genes on the Z-chromosome.

### Effective Upregulation of the Male Z-Chromosome Occurs Only during the Adult Stage

We found that although the Z-chromosome to autosome expression ratio increased with each developmental stage, substantial convergence between Z-linked and autosomal genes in males was only achieved during the adult stage. In larvae and pupae, Z-chromosome to autosome dosage equilibration was observed only for sex-biased genes. These results suggest that during larval development, a period during which sexual dimorphism is not yet inordinately pronounced, genome-level expression regulation could be adjusted to the heterogametic chromosomal layout through Z-chromosome hypoactivation in males. The relatively small number of sex-biased genes could be regulated on a case by case basis in each sex, as has previously been observed in birds (e.g., Mank and Ellegren 2009; Zimmer et al. 2016). It is also possible that a fraction of (highly expressed) genes escapes downregulation in the same way as during human X-inactivation (Carrel et al. 1999).

Our results show that widespread downregulation of the Z-chromosome was already initiated in male larva, which means that downregulation apparently is initiated earlier on in the ontogenetic trajectory. In the distant relative, the silkmoth *B. mori*, downregulation of the Z-chromosome actually occurs already at 120 h after oviposition (Gopinath et al. 2017). The process of dosage compensation has been shown to be tightly linked to sex determination, apparently through pleiotropic effects of the genes *FEM* and *MASC* (Kiuchi et al. 2014). Apart from this, little is known about the genetic regulation and onset of dosage compensation in Lepidoptera. Our attempts to find orthologs of *MASC* and *FEM* in *L. sinapis* were unsuccessful and previous orthology searches in *Heliconius* did not pinpoint any obvious candidate gene (Walters et al. 2015). The reason for not identifying *FEM* and *MASC* homologs could be a consequence of deep divergence between *B. mori* and butterflies (Kawahara and Breinholt 2014; Talla et al. 2017). Sex-determining genes can gain or lose function during the course of evolution (Tanaka et al. 2007) and may be relocated to different chromosomes or have paralogs with similar sequence motifs, making orthology identification over deep divergence times challenging.

### Dosage Compensation Mechanism in Adult Females

We observed convergence in expression between unbiased autosomal and Z-linked genes in adult females (supplementary fig. S11*B*, Supplementary Material online). This would imply activation of a separate dosage compensation mechanism in the heterogametic sex during its later development. The presence of such a distinct dosage compensation mechanisms in males and females could be controlled by differential splicing of the master sex-determination switch gene (e.g., Kelley et al. 1997). The MSL complex is one of the best studied dosage compensation mechanisms in insects (e.g., Kuroda et al. 2016). In *Drosophila*, this complex likely regulates X-chromosome hyperactivation in males (the heterogametic sex) by mediating transcription activity (e.g., Kuroda et al. 2016). Although earlier attempts to find homologs to the five MSL core genes in Lepidoptera provided mixed results (Xia et al. 2004; Smith et al. 2014), homologs to these genes have been identified in recent years in several moths and butterflies (supplementary table S15, Supplementary Material online). In *L. sinapis*, we found that homologs of all five genes and *MSL1*, *MSL2*, *MLE*, and *MOF* were upregulated in adult females. *MSL2* expression in males was particularly low, echoing similar results observed in *Drosophila*, where *MSL2* is absent in females (Conrad and Akhtar 2012). Because assembly of the MSL complex is coordinated by the *MSL2* protein (Conrad and Akhtar 2012), this implies the MSL complex is likely absent in male *L. sinapis*. Our results show that MSL upregulation in females occurs only during the adult stage. This suggests that the onset of dosage compensation in females occurs only when specimens reach sexual maturity and both Z-chromosome and autosomal expression are strongly sex biased.

Although the presence of the MSL complex in adult female wood whites would suggest that partial Z:autosome dosage tuning is achieved by an increase in Z-chromosome expression, our results indicate that this is not the case. The reduced difference in expression between chromosomal classes in adult females was instead due mostly to a decrease in overall expression in non-sex-biased genes located on autosomes. This is at odds with the prevailing understanding of the role played by the MSL complex in male *Drosophila*, where it is believed to promote upregulation of X-linked genes. An alternative hypothesis proposes that X-monosomy in *Drosophila* triggers an autosomal inverse effect—an increase in male autosomal expression vis-a-vis females due to the availability of a large pool of unused transcription factors—and it is then hypothesized that the MSL complex keeps autosomal expression in check by sequestrating histone modifiers from the autosomes (Sun et al. 2013; Birchler 2016; Samata and Akhtar 2018). The results observed in adult females could hence be the result of a similar mechanism being active in wood whites, with the MSL complex reducing the difference between autosomal and Z-linked expression (supplementary fig. S11*B*, Supplementary Material online). In pupae, a period during which *MSL2* expression was close to zero in females and therefore assembly of the MSL complex is improbable, autosomal expression of unbiased genes expressed in both sexes was higher in females than in males. This is in accordance with expectations under the inverse effect model. During the larval stage, the *MSL2* gene was only weakly expressed in females, but male Z-chromosome to autosome expression was also reduced, resulting in effective Z-monosomy in both sexes. Our results show that autosomal expression for unbiased genes was indeed similar for male and female larvae and comparable to the values observed in female pupae. Our data suggest that autosomal and Z-linked expressions of unbiased genes expressed in adults were lower in females than in males (supplementary fig. S11*B*, Supplementary Material online). However, this does not necessarily equate with decreased protein production in females, as it could be compensated by enhanced rates of protein synthesis during translation. In fact, the GO analysis provided evidence for a major surge in ribosomal assembly and RNA processing in adult females, revealing a burst in cytoplasmic translation activity.

A pertinent explanation for differences in dosage compensation regulation across lineages relates to the type and availability of expression modulators that can be coopted for chromosomal dosage tuning. Several of the MSL core genes have been found in a large number of species where they are implicated in numerous regulatory activities (Smith et al. 2005; Rea et al. 2007). This suggests a high degree of functional conservation as well as of pleiotropy. However, some MSL key genes are highly divergent across species. In the mosquito *Anopheles gambiae*, homologs to all five MSL genes have been identified and there is complete X to autosomal compensation in both sexes, despite considerable sequence divergence compared with *MSL1* and *MSL2* in *Drosophila* (Rose et al. 2016). Significant *MSL1* and *MSL2* divergence is also observed for several lepidopteran species, including in *L. sinapis* (supplementary table S15, Supplementary Material online). It cannot be ruled out that substitutions in the MSL genes could impair the assembly and/or functioning of the MSL complex. Dosage regulation in *Anophelesgambiae* and in Lepidoptera might therefore have different genetic underpinnings. However, divergence could also be coupled with changes in the function of the MSL genes and ultimately of the complex itself. This would fit the results observed in *L. sinapis*, namely a reduction in Z to autosomal expression in adult females that falls short of complete dosage compensation—but which occurs only when all the five core MSL genes are highly expressed, whereas in males MSL assembly is impaired by silencing of the *MSL2* gene across all developmental stages. Naturally, MSL assembly is unlikely in species that lack some of its key genes. Unlike most other Lepidopterans, both *M. sexta* and *P. machaon*, show complete Z:autosome dosage compensation and their Z-chromosome is not enriched for male-biased genes (Smith et al. 2014; Huylmans et al. 2017). It is therefore plausible that the molecular basis of the dosage compensation mechanism in *M. sexta* and *P. machaon* differs from that in *L. sinapis*. In fact, although *MLE* and *MOF* were detected in *M. sexta*, so far none of the other MSL key genes have been observed in these species (Smith et al. 2014).

### Potential Limitations and Caveats

Changes in *median* expression across sex and stages were not always matched by equivalent changes in *mean* expression, which tend to vary more widely. Given the skewed distribution of gene expression data (the majority of genes were expressed at low levels, whereas a few had a very high expression level), the mean estimate is more affected by filtering processes as compared with the median. Hence, median expression levels are more robust for comparisons across replicates/treatments/stages in general. Nevertheless, the trends in mean Z-linked and autosomal expression were in general consistent with the observations made using medians, thus supporting the results presented above.

It should be noted that the analysis was made based on homology between *L. sinapis* scaffolds and the *Heliconius* reference genome. These two species are distantly related with a divergence time >90–100 Myr (Talla et al. 2017; Espeland et al. 2018) and have slightly different karyotypes due to several fusions in the *Heliconius* lineage (Davey et al. 2016). Hence, some *L. sinapis* genes may have been assigned to the wrong chromosome. However, the relatively strong homogeneousness of autosomal expression and the consistent differences in expression levels between autosomal and Z-linked genes in all comparisons suggest that the assignment of chromosome location is relatively robust. If chromosome rearrangements between autosomes and the Z-chromosome had been extensive in either lineage after the split of the two species, it would have been very unlikely to observe such strong effects of sex linkage. This will of course only be possible to verify when a *L. sinapis* genome assembly with chromosome linkage information becomes available.

One potential limitation of this study is the utilization of RNA-seq data sampled from whole specimens. There is an obvious potential for different expression patterns in various tissues and because gonads often make up a relatively large proportion of the body mass in insects, the use of whole body samples could bias the interpretation of dosage compensation (Gu et al. 2017). As an example, in *B. mori* and *P. interpunctella*, both male and female Z-chromosome to autosome ratios were basically similar in all tissues, except in gonads where the ratio was considerably higher in males (Huylmans et al. 2017). However, this discrepancy disappeared after controlling for sex-biased genes, resulting in overall balance between male and female expression (Huylmans et al. 2017). Furthermore, as this correction made the Z:autosome ratios roughly similar across tissue (and constantly below 1), the interpretation that Z-linked expression is downregulated compared with autosomes would not have changed if whole body samples had been used. In addition, the observation that the expression difference between autosomal and Z-linked genes in *L. sinapis* was consistent across developmental stages, despite metamorphosis and more or less unique gene sets being active in each respective stage (Leal et al. 2018), suggests that variation in tissue specific expression should not have a substantial effect on the main results of this study.

## Conclusions

The results presented in this article provide compelling evidence that analysis of chromosomal expression at the aggregate level may mask more subtle patterns of dosage compensation. In *L. sinapis*, a breakdown of genes into expression categories revealed a multilayered dosage compensation structure where specific autosomal and Z-linked gene categories were found to have attained full equalization. We further provide evidence that gene dosage mechanisms may lead to distinct dosage compensation patterns in different species, even when underpinned by the same MSL complex molecular machinery. Our findings suggest that the exact strategy employed to balance expression across sexes and chromosomal classes in Lepidoptera is the result of a delicate balance between several genomic features, including masculinization/feminization of the Z-chromosome and the availability of expression modulators that can be coopted for dosage tuning as sex-chromosomes differentiate.

## Supplementary Material

Supplementary data are available at *Genome Biology and Evolution* online.

## Supplementary Material

evz176_Supplementary_Data

## Notes

**Data deposition** **:** This project has been deposited at the European Bioinformatics Institute (EMBL-EBI) data base ArrayExpress (https://www.ebi.ac.uk/arrayexpress/) under the accession E-MTAB-6454 (Leal et al. 2018). All in-house developed scripts are available at github.com/larshook and github.com/LLN273.
