# Supplementary material for: Multilayered Tuning of Dosage Compensation and Z-Chromosome Masculinization in the Wood White (Leptidea sinapis) Butterfly
Source: Genome Biol Evol. 2019 Aug 10;11(9):2633–52. doi: 10.1093/gbe/evz176 (PMC6761951; doi:10.1093/gbe/evz176)
Supplement: evz176_Supplementary_Data [file evz176_supplementary_data.pdf]

# Supplementary material

## Correlation across biological replicates

To design proper analyses of dosage compensation and sex-biased expression, we initially assessed if expression levels across biological replicates within treatment groups were correlated. The rationale for this was that replicates could be merged to calculate a treatment group median and mean (and variance) for comparisons across groups. The FPKM-normalized gene expression levels for samples within each group were all highly significantly correlated (Spearman's $\rho > 0.67$; p-values $< 2.2*10^{-16}$). The strength of the correlation was slightly lower in pupae ($\rho$ = 0.67-0.81) as compared to larvae ($\rho$ = 0.89-0.91) and adults ($\rho$ = 0.89-0.93) (Supplementary Figure S1). Based on this strong homogeneity of expression levels across biological replicates, the per group mean for each gene was calculated and used in subsequent analyses.

**Figure S1.**

Correlation of gene expression levels of samples within each group for larvae (A), pupae (B) and adults (C). Female samples are in top rows and male samples in bottom rows in each respective panel. All correlations were highly significant ($p < 2.2 \times 10^{-16}$). Spearman's rank correlation test used to compute the correlation coefficient (ρ) for each pairwise comparison.

A

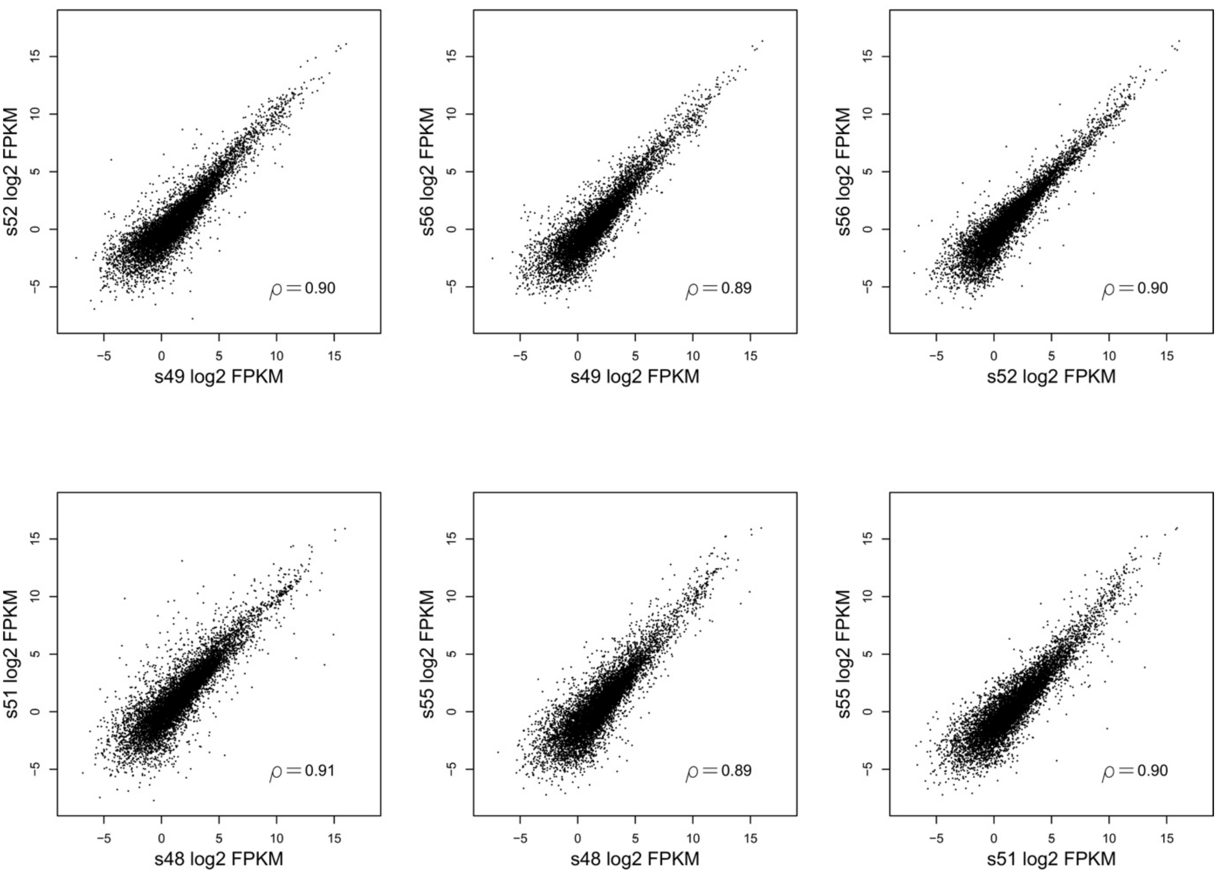

**B**

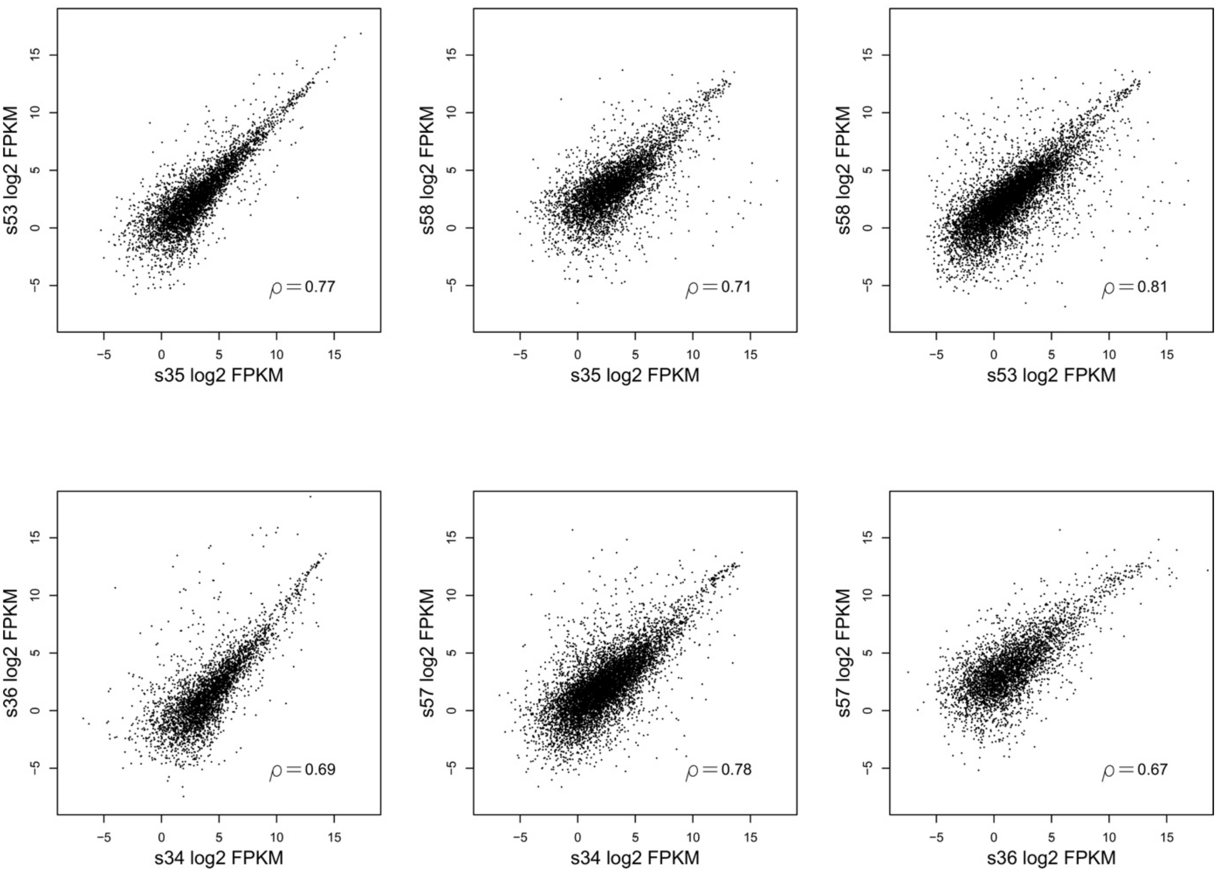
s53 log2 FPKM
s35 log2 FPKM
ρ = 0.77
s58 log2 FPKM
s35 log2 FPKM
ρ = 0.71
s58 log2 FPKM
s53 log2 FPKM
ρ = 0.81
s36 log2 FPKM
s34 log2 FPKM
ρ = 0.69
s57 log2 FPKM
s34 log2 FPKM
ρ = 0.78
s57 log2 FPKM
s36 log2 FPKM
ρ = 0.67

**C**

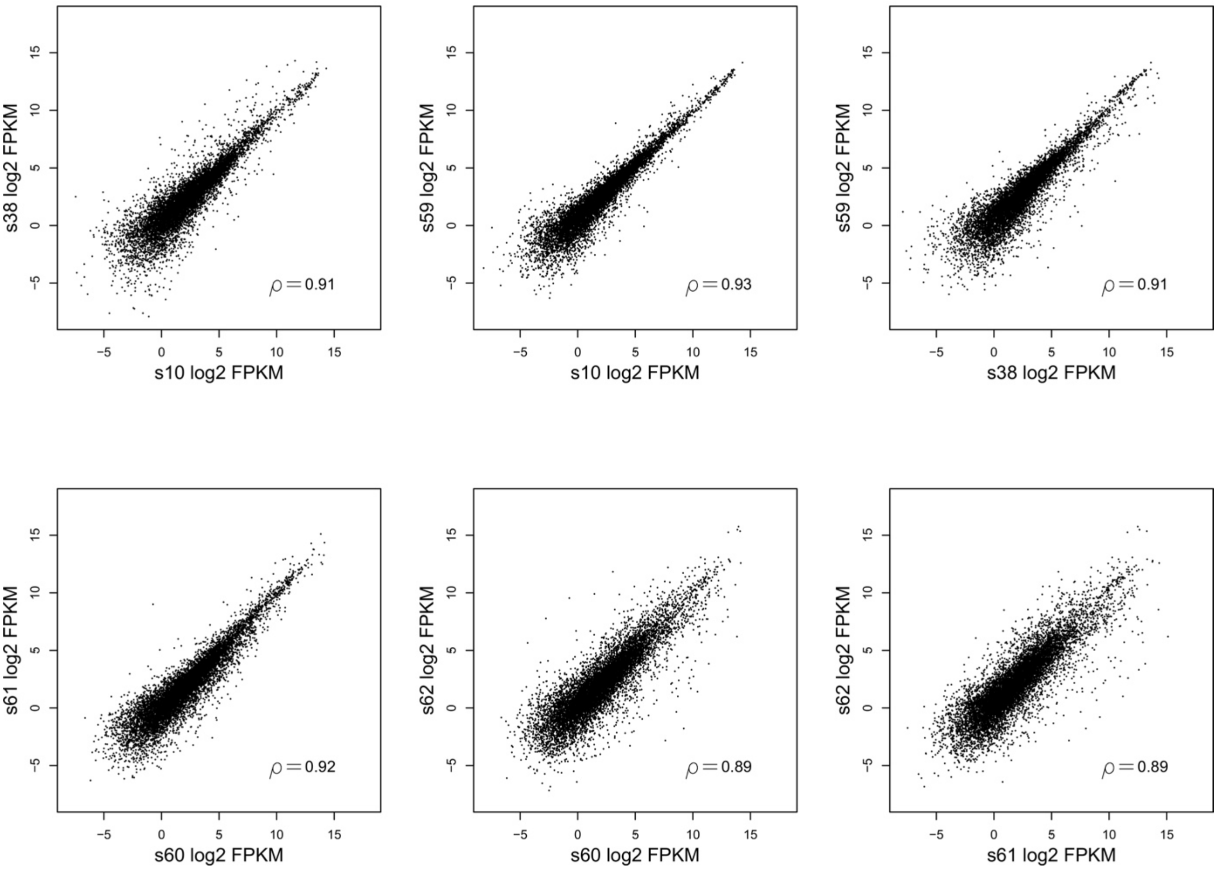
s38 log2 FPKM
s10 log2 FPKM
ρ = 0.91
s59 log2 FPKM
s10 log2 FPKM
ρ = 0.93
s59 log2 FPKM
s38 log2 FPKM
ρ = 0.91
s61 log2 FPKM
s60 log2 FPKM
ρ = 0.92
s62 log2 FPKM
s60 log2 FPKM
ρ = 0.89
s62 log2 FPKM
s61 log2 FPKM
ρ = 0.89

**Figure S2.**

Scatterplot of male versus female autosomal and Z-linked expression levels with local regression (lowess) lines. Only genes with FPKM > 0 in both sexes were included. Correlation coefficients: larva (autosomes: $\rho = 0.93$; Z: $\rho = 0.85$); pupa (autosomes: $\rho = 0.85$; Z: $\rho = 0.73$); adult (autosomes: $\rho = 0.80$; Z: $\rho = 0.73$), in all cases significant ($p < 2.2*10^{-16}$).

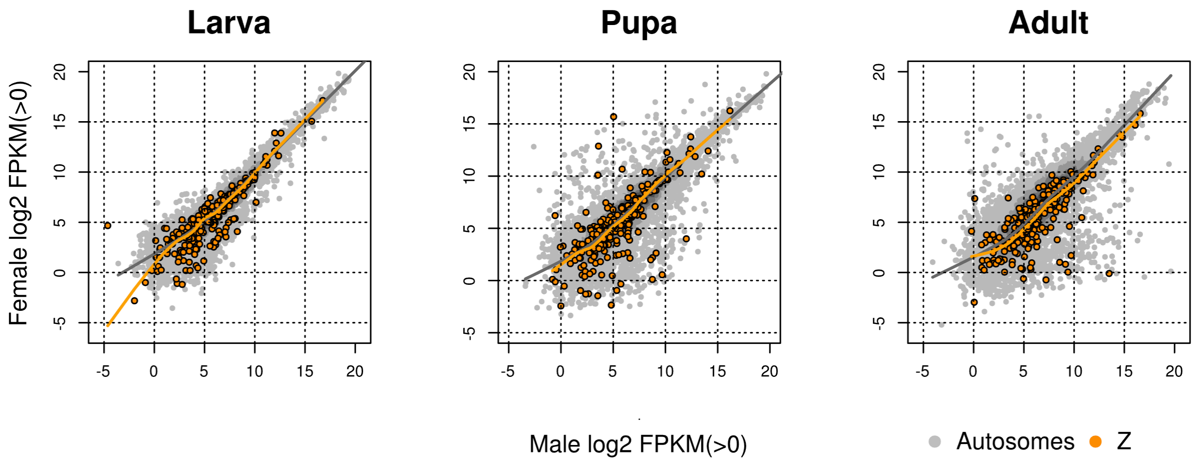

**Figure S3.**

Bar plot showing the frequency of significant differences for each pairwise chromosome comparison, evaluated with Dunn's test of multiple comparison. Analyses were carried out separately for each sex and developmental stage. Zero-expression genes were removed separately for males and females. Exact p-values for each test are presented in Supplementary material, Table S4.

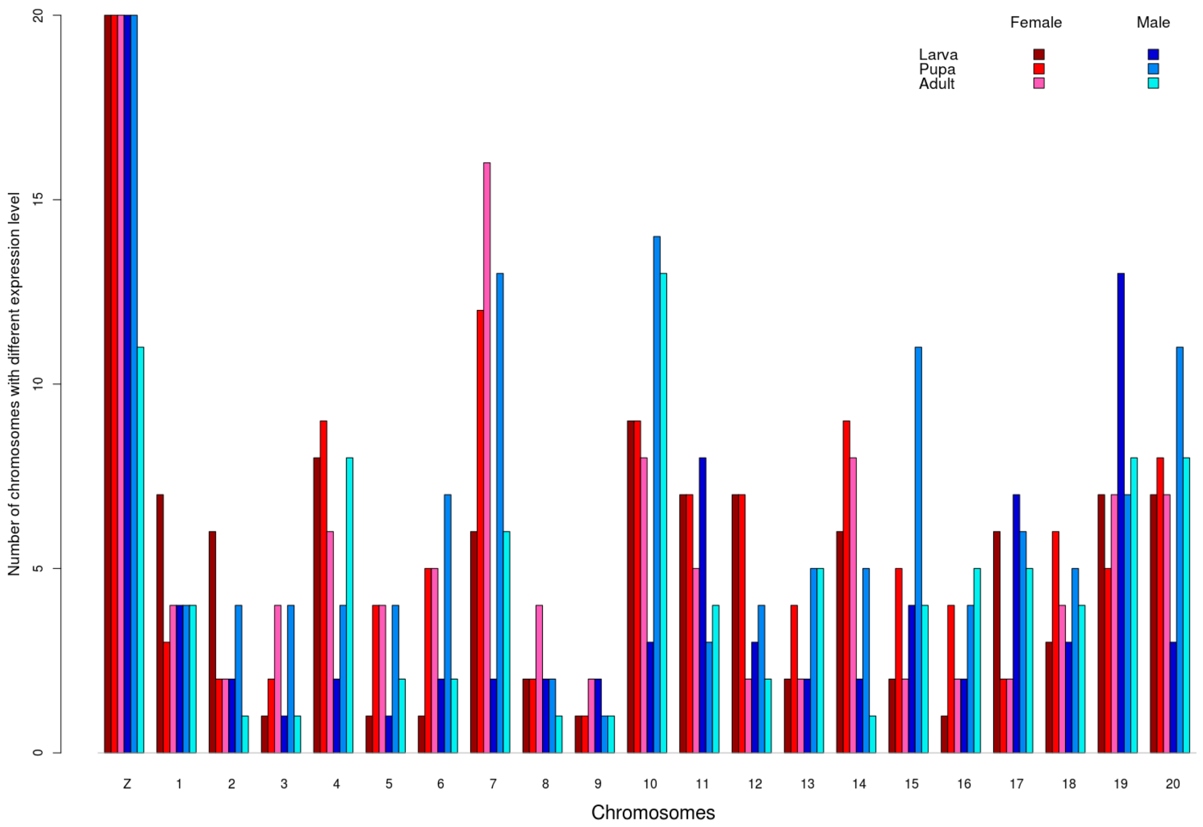

**Figure S4.**

Density distribution of male to female FPKM-ratios for autosomal (grey) and Z-linked (orange) genes. Dashed lines mark the median of ratios (Table 2) for each respective chromosome class. The asterisk marks a significant difference between ratio distributions in the adult stage (Mann-Whitney *U*-test; p-value = $1.0*10^{-8}$). Distributions were not significantly different for larvae (p-value = 0.82) and pupae (p-value = 0.44). Only genes with FPKM > 0 in both sexes were included.

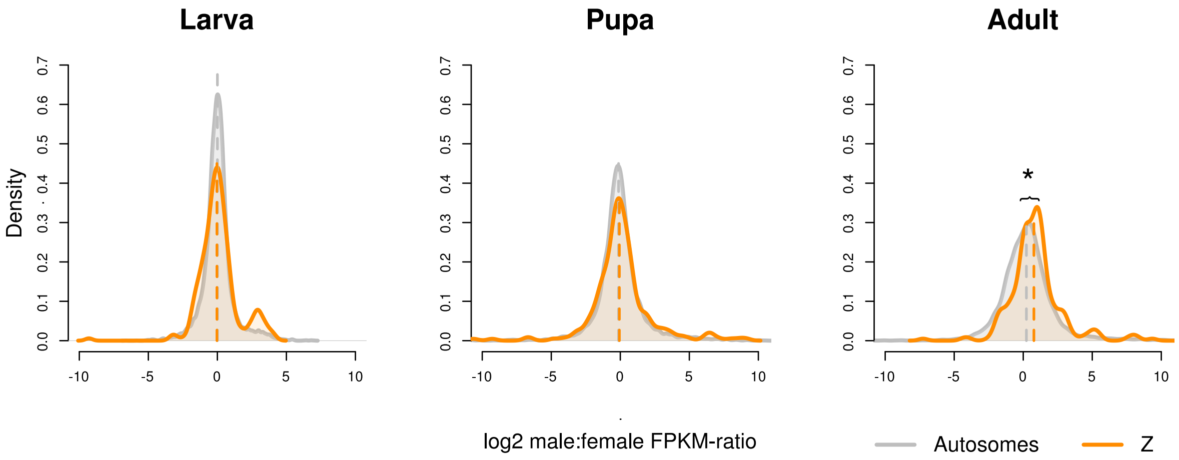

**Figure S5.**

Differentially expressed genes across sexes in larva (A), pupa (B) and adult (C). The x-axis shows the log2 fold change and negative log10 p-values are plotted along the y-axis. Male-biased genes (MBG) are blue, female-biased genes (FBG) are red and unbiased genes are grey. The darker grey color is a result of higher density of points in that area of the graph (the x-axis is centered at zero fold-change). The horizontal dashed line represents the FDR-adjusted p-value significance level (0.05) and the vertical lines show the |log2 fold change| threshold used (> 1) to define male- or female-biased genes. The inset shows a zoom-in of the data from larvae.

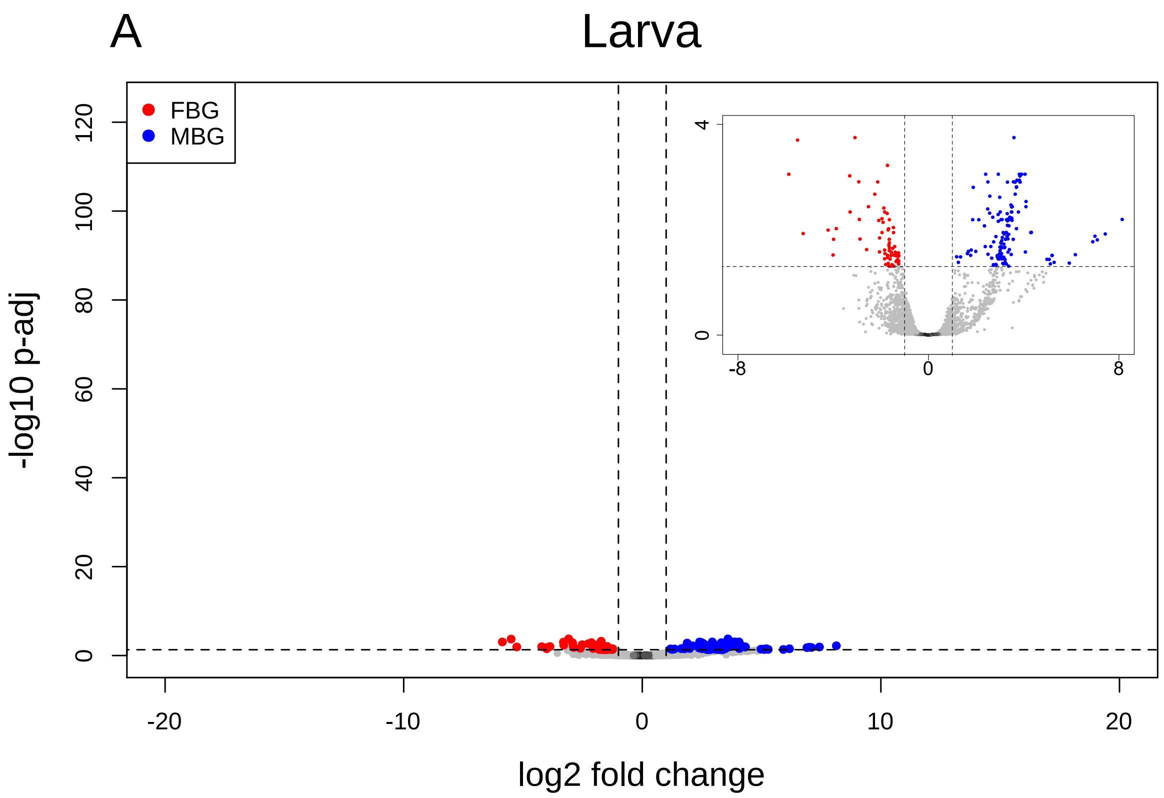

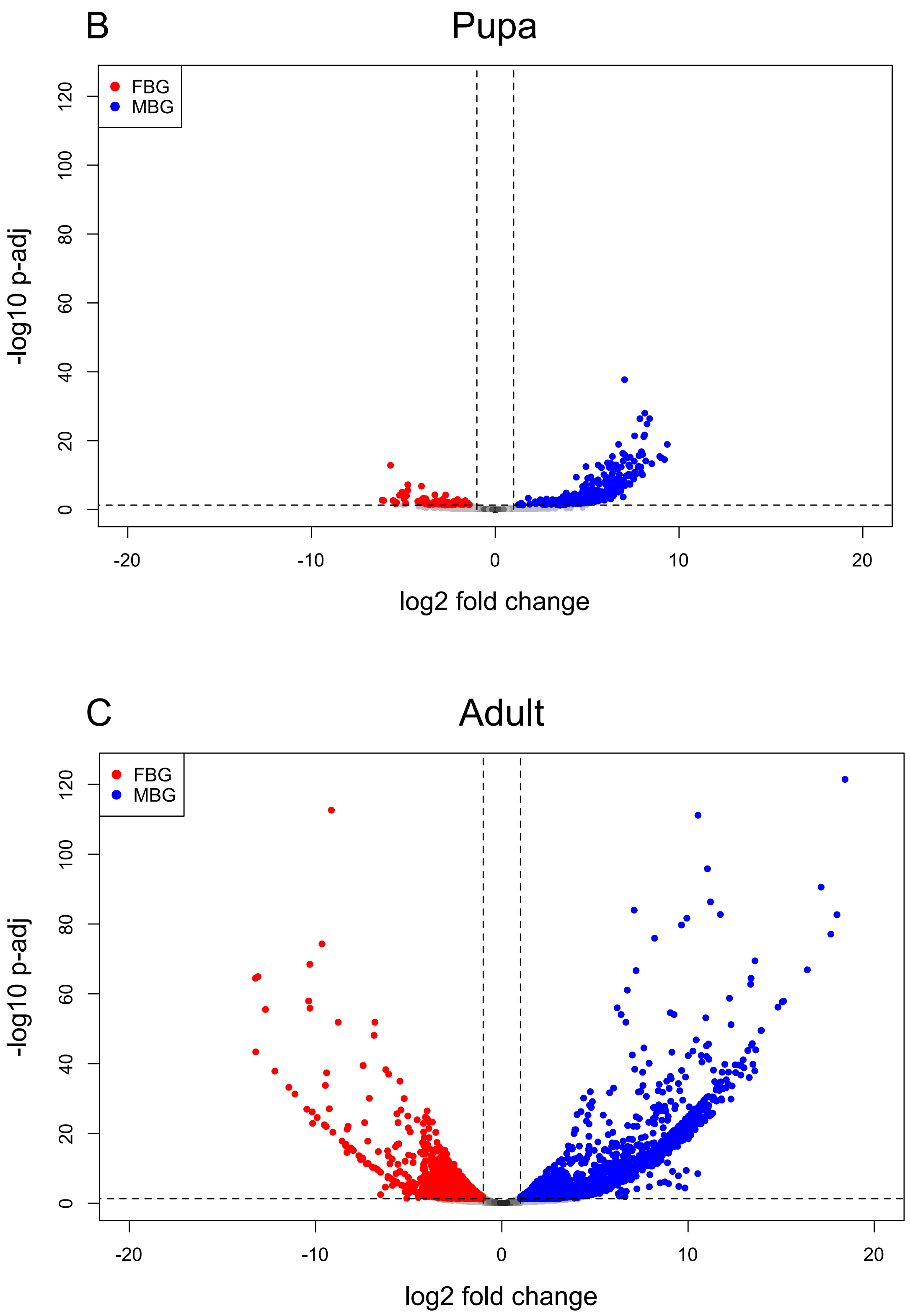
B
Pupa
FBG
MBG
120
100
80
60
40
20
0
-log10 p-adj
-20
-10
0
10
20
log2 fold change
C
Adult
FBG
MBG
120
100
80
60
40
20
0
-log10 p-adj
-20
-10
0
10
20
log2 fold change

**Figure S6.**

Quartile based analysis of expression levels of Z-linked genes, before (A) and after (B) excluding genes with significant sex-biased expression. Genes were separated into quartiles based on maximum male or female expression. Only genes with FPKM > 0 in both sexes were included. Asterisks mark significant differences in expression levels (Supplementary Tables S9 and S10). Boxes represent the interquartile range and whiskers extend to 1.5x the interquartile range. Notches represent the 95% confidence interval of median (black bar) expression. Outliers have been removed from the plot. Significance levels are indicated by asterisks; * < 0.05; ** < 0.01; *** < 0.001.

**A**

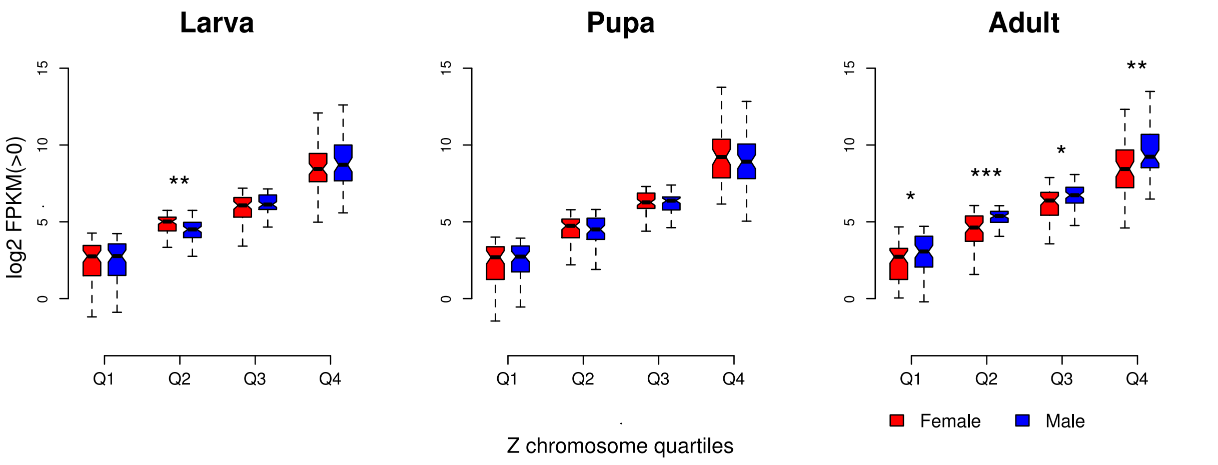


**B**

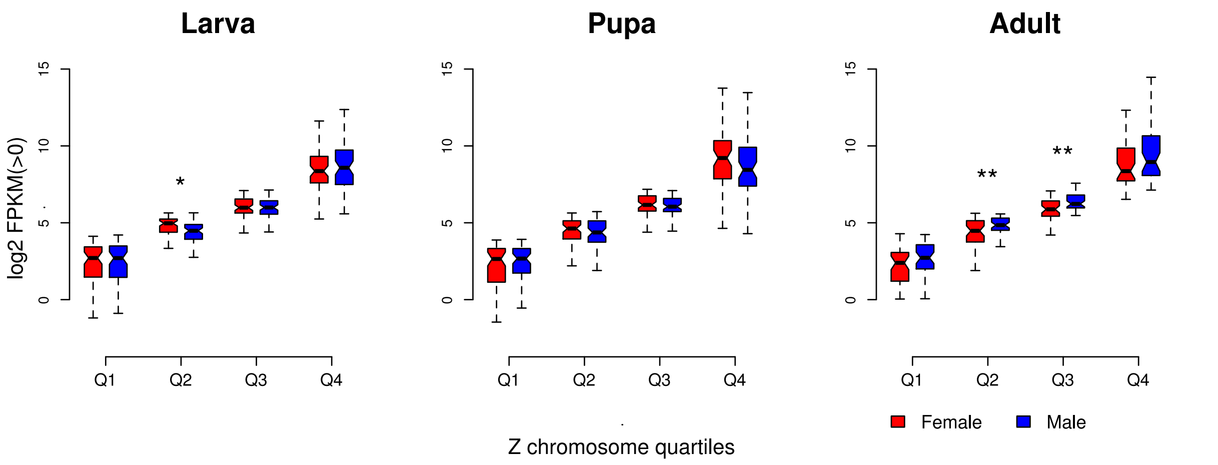

**Figure S7.**

Top-35 most significant GO categories based on the set of male-biased (A) and female-biased (B) significantly expressed genes detected in larvae. Symbol size is proportional to the number of significantly expressed genes assigned to a GO category (significant genes). The smallest symbol size corresponds to one gene. Symbol color reflects the ratio between the number of significant genes associated to a particular GO term and the total number of annotated genes associated to that same GO category (enrichment score). Terms highlighted in blue are associated to sperm synthesis in males. Data based on *de novo* transcriptome assembly.

A

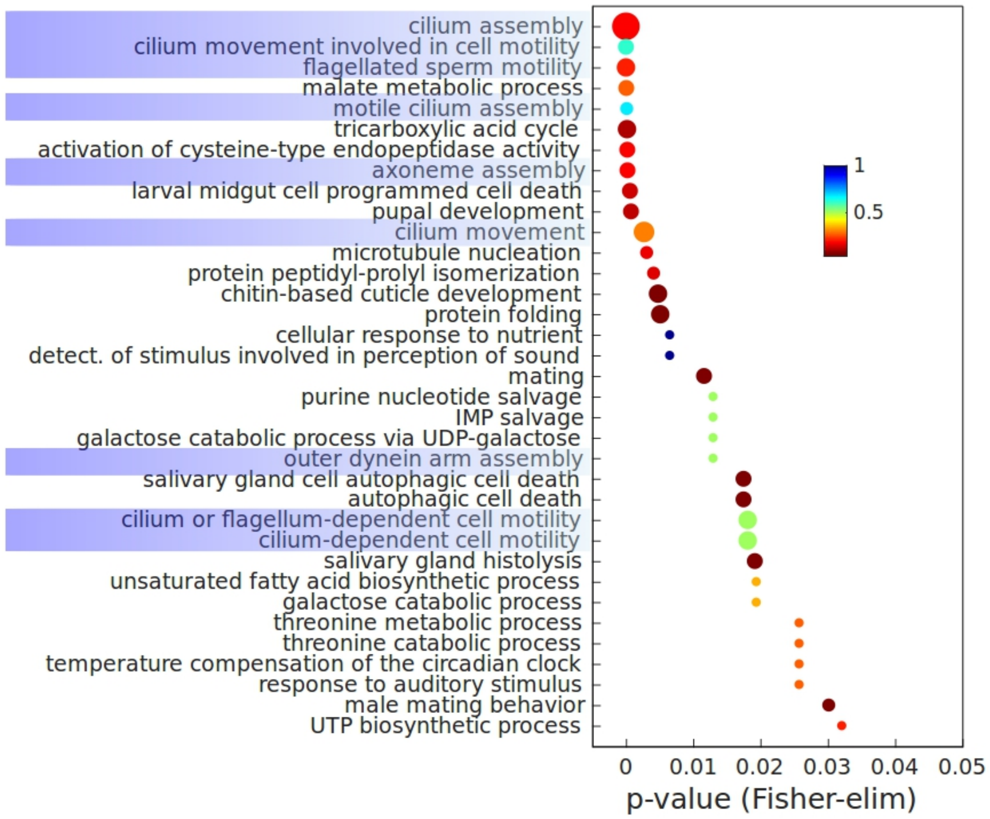

B

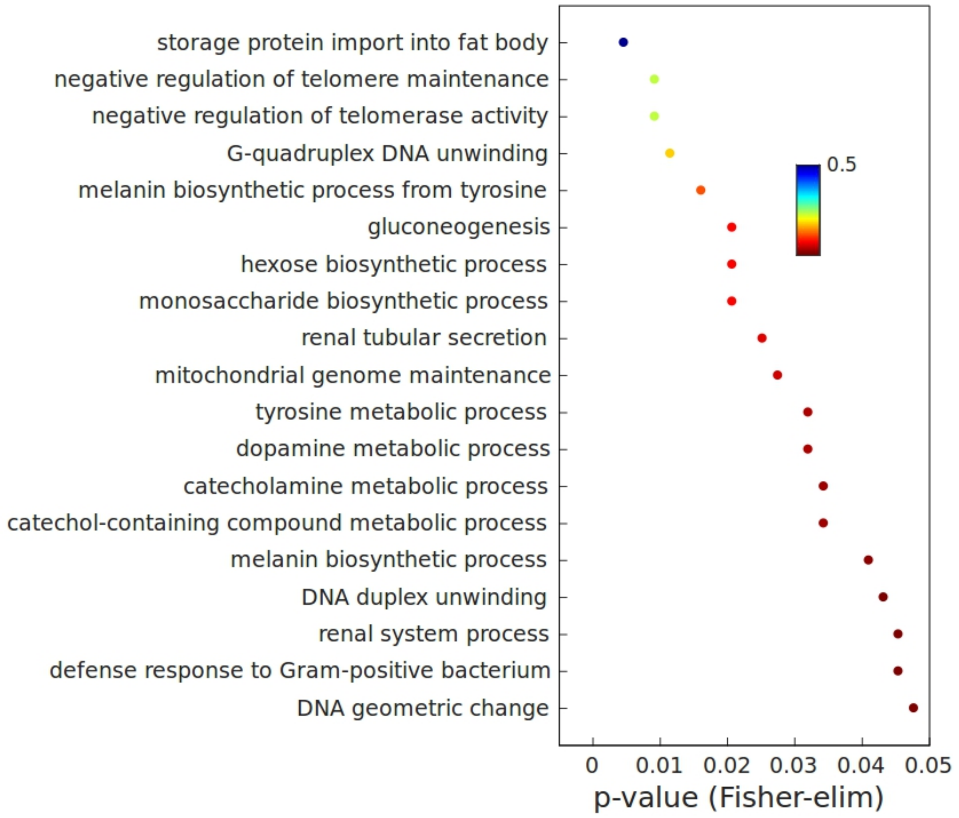

storage protein import into fat body
negative regulation of telomere maintenance
negative regulation of telomerase activity
G-quadruplex DNA unwinding
melanin biosynthetic process from tyrosine
gluconeogenesis
hexose biosynthetic process
monosaccharide biosynthetic process
renal tubular secretion
mitochondrial genome maintenance
tyrosine metabolic process
dopamine metabolic process
catecholamine metabolic process
catechol-containing compound metabolic process
melanin biosynthetic process
DNA duplex unwinding
renal system process
defense response to Gram-positive bacterium
DNA geometric change
0.5
0
0.01
0.02
0.03
0.04
0.05
p-value (Fisher-elim)

**Figure S8.**

Top-35 most significant GO categories based on the set of male-biased (A) and female-biased (B) significantly expressed genes detected in pupae. Symbol size is proportional to the number of significantly expressed genes assigned to a GO category (significant genes). The smallest symbol size corresponds to one gene. Symbol color reflects the ratio between the number of significant genes associated to a particular GO term and the total number of annotated genes associated to that same GO category (enrichment score). Terms highlighted in blue are associated to sperm synthesis in males. Data based on *de novo* transcriptome assembly.

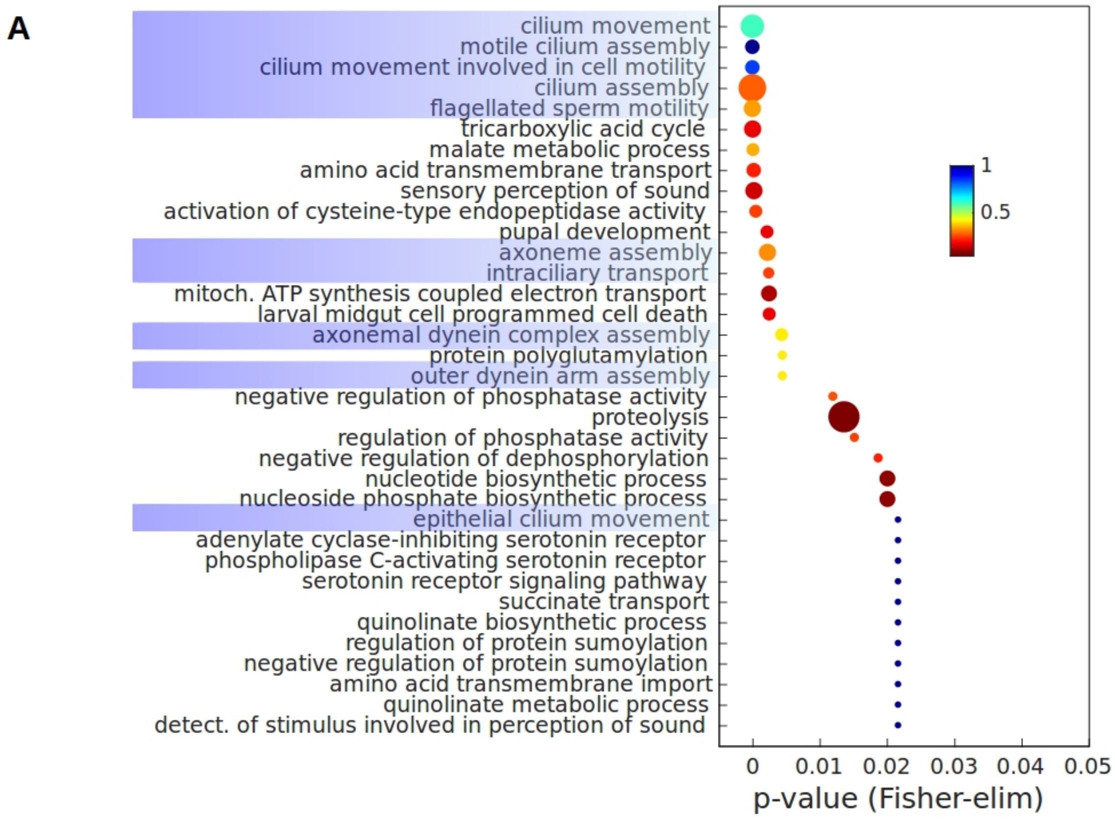

**B**

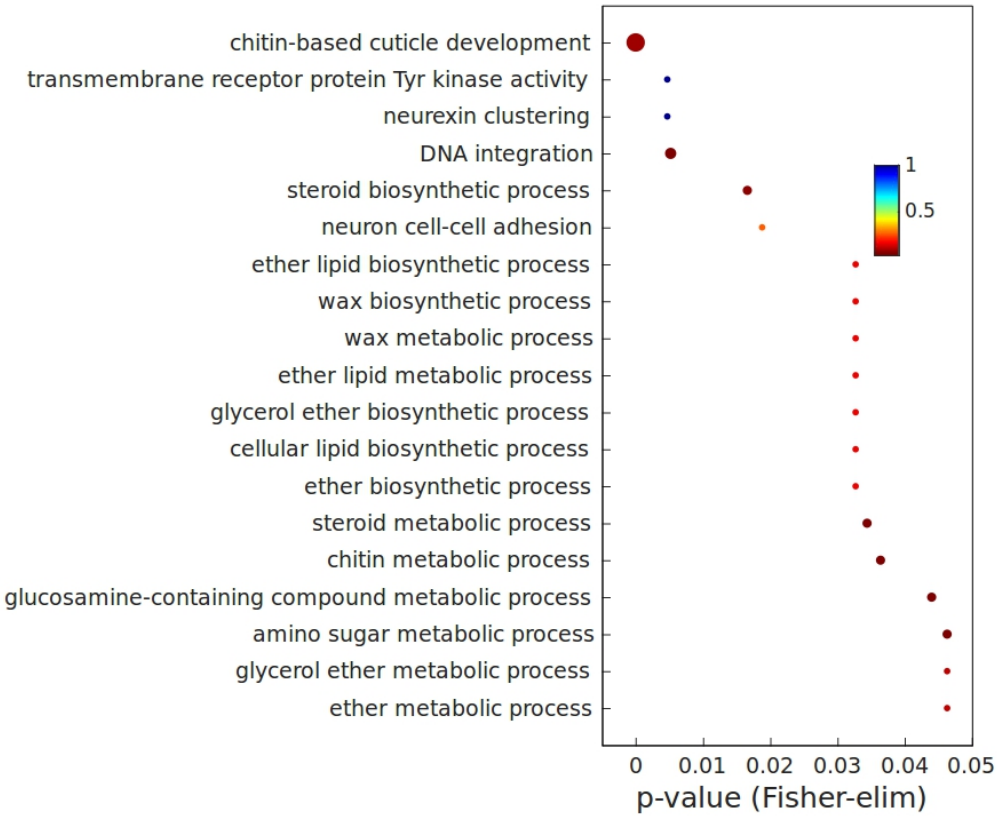
chitin-based cuticle development
transmembrane receptor protein Tyr kinase activity
neurexin clustering
DNA integration
steroid biosynthetic process
neuron cell-cell adhesion
ether lipid biosynthetic process
wax biosynthetic process
wax metabolic process
ether lipid metabolic process
glycerol ether biosynthetic process
cellular lipid biosynthetic process
ether biosynthetic process
steroid metabolic process
chitin metabolic process
glucosamine-containing compound metabolic process
amino sugar metabolic process
glycerol ether metabolic process
ether metabolic process
1
0.5
0
0.01
0.02
0.03
0.04
0.05
p-value (Fisher-elim)

**Figure S9.**

Top-35 most significant GO categories based on the set of male-biased (A) and female-biased (B) significantly expressed genes detected in adults. Symbol size is proportional to the number of significantly expressed genes assigned to a GO category (significant genes). The smallest symbol size corresponds to two genes. Symbol color reflects the ratio between the number of significant genes associated to a particular GO term and the total number of annotated genes associated to that same GO category (enrichment score). Terms highlighted in blue are associated to sperm synthesis in males; terms highlighted in pink are associated to transcript replication and translation in females. Data based on *de novo* transcriptome assembly.

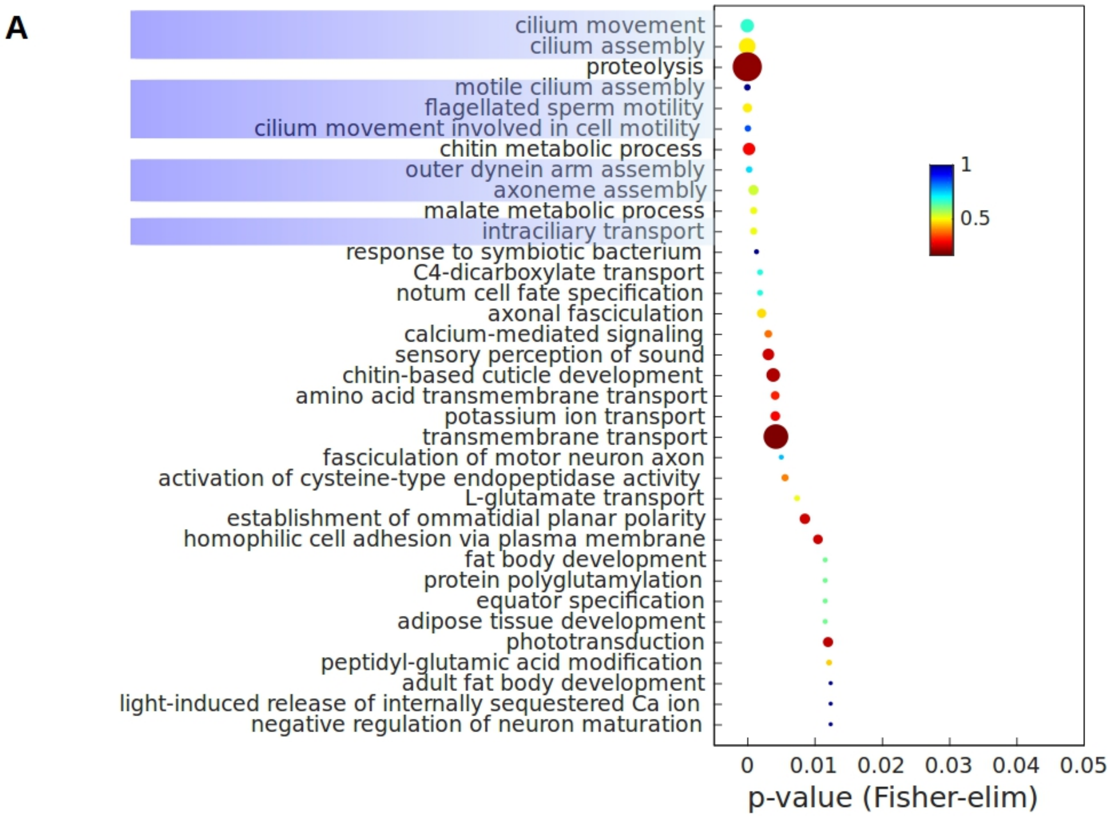

B

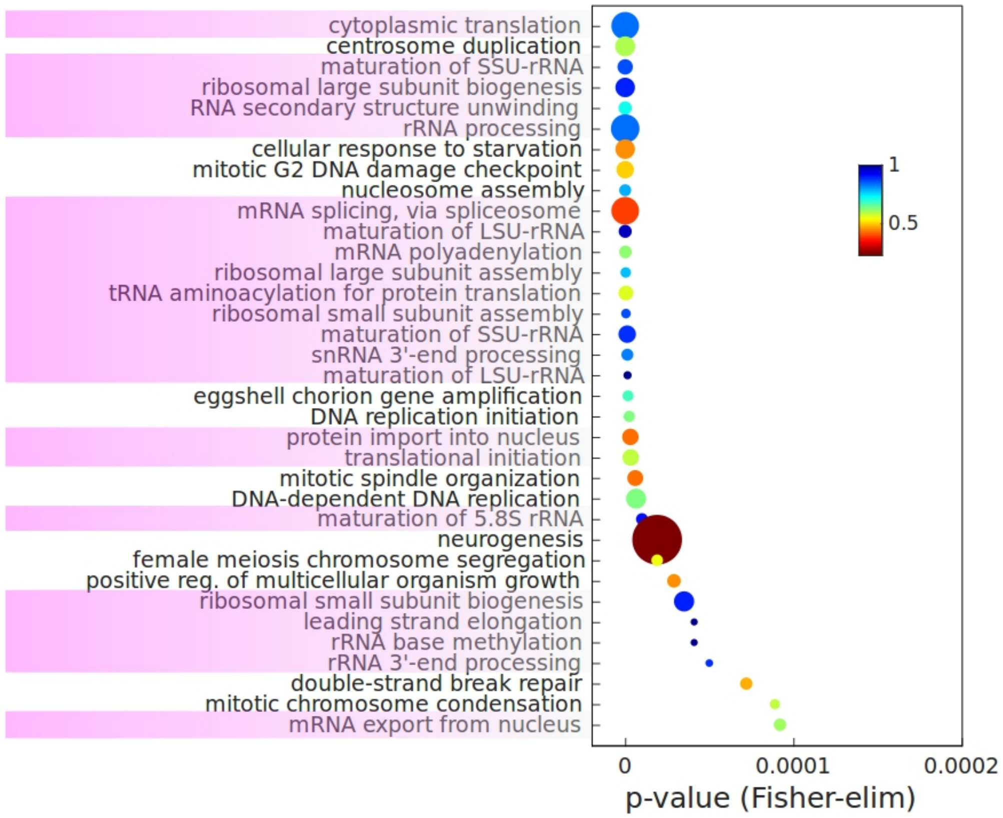
cytoplasmic translation
centrosome duplication
maturation of SSU-rRNA
ribosomal large subunit biogenesis
RNA secondary structure unwinding
rRNA processing
cellular response to starvation
mitotic G2 DNA damage checkpoint
nucleosome assembly
mRNA splicing, via spliceosome
maturation of LSU-rRNA
mRNA polyadenylation
ribosomal large subunit assembly
tRNA aminoacylation for protein translation
ribosomal small subunit assembly
maturation of SSU-rRNA
snRNA 3'-end processing
maturation of LSU-rRNA
eggshell chorion gene amplification
DNA replication initiation
protein import into nucleus
translational initiation
mitotic spindle organization
DNA-dependent DNA replication
maturation of 5.8S rRNA
neurogenesis
female meiosis chromosome segregation
positive reg. of multicellular organism growth
ribosomal small subunit biogenesis
leading strand elongation
rRNA base methylation
rRNA 3'-end processing
double-strand break repair
mitotic chromosome condensation
mRNA export from nucleus
1
0.5
0
0.0001
0.0002
p-value (Fisher-elim)

**Figure S10.**

Average expression levels across developmental stages observed in females (left) and males (right) for genes *MSL1*, *MSL2*, *MSL3*, *MOF* (A) and *MLE*, *TOPO2*, and *LOQS* (B). Counts were normalized by library size and corrected for batch effects. Data based on *de novo* transcriptome assembly.

**A**

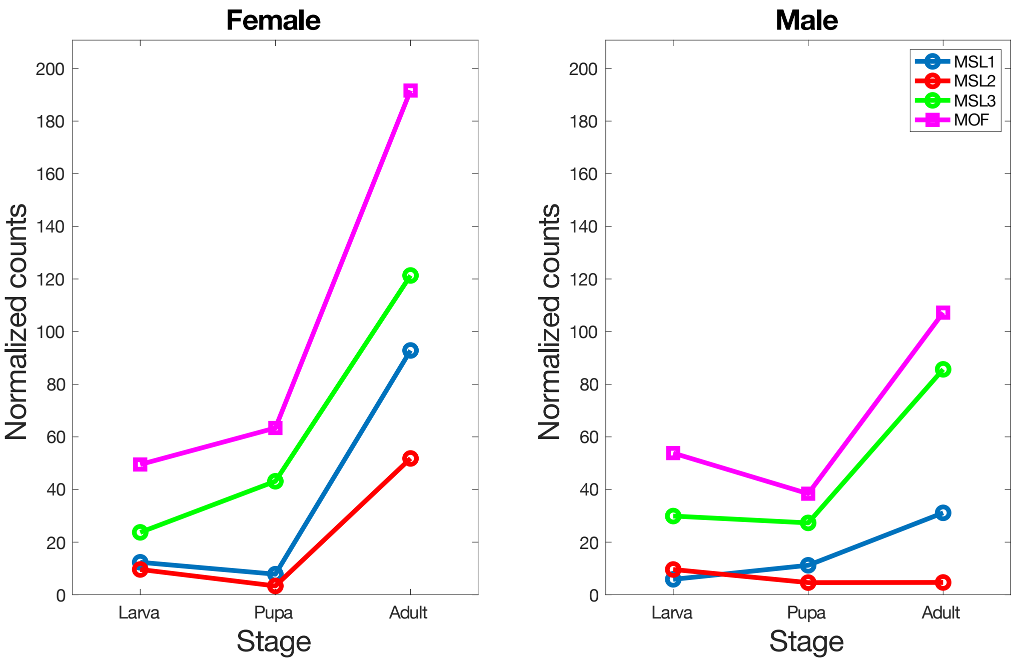

B

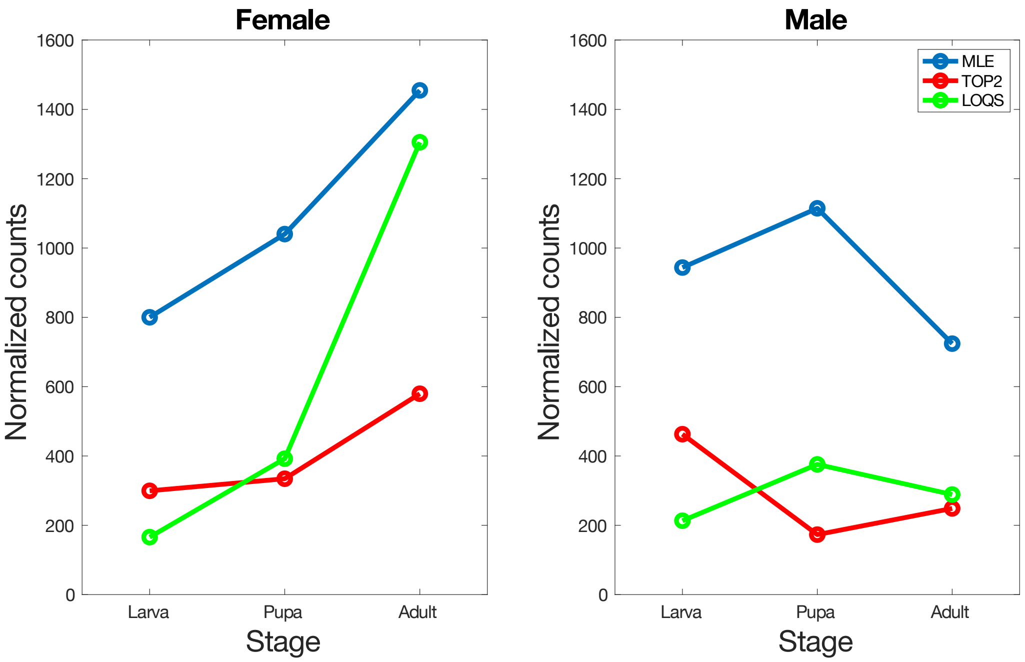

Female
Male
1600
1400
1200
1000
800
600
400
200
0
Normalized counts
Larva
Pupa
Adult
Stage
MLE
TOP2
LOQS

**Supplementary Figure S11.**

A schematic representation of the multilayered tuning of gene dosage for different gene classes in *L. sinapis*. (A) Autosomal and Z-linked gene classes with matching expression levels, shown separately for adult males and females. (B) Development of autosomal and Z-linked expression across developmental stages. In female larvae and pupae, no general dosage compensation mechanism was in place and Z-monosomy lead to high autosomal expression due to the autosomal inverse effect. Z-chromosome hypoexpression in male larvae produced a similar effect. Multilayered dosage compensation became fully operational only during the adult stage, leading to a reduction in the difference between autosomal and Z-linked expression. Enhanced levels of cytoplasmic translation (t) in adult females brought rates of protein synthesis closer to parity with adult males.

**A**

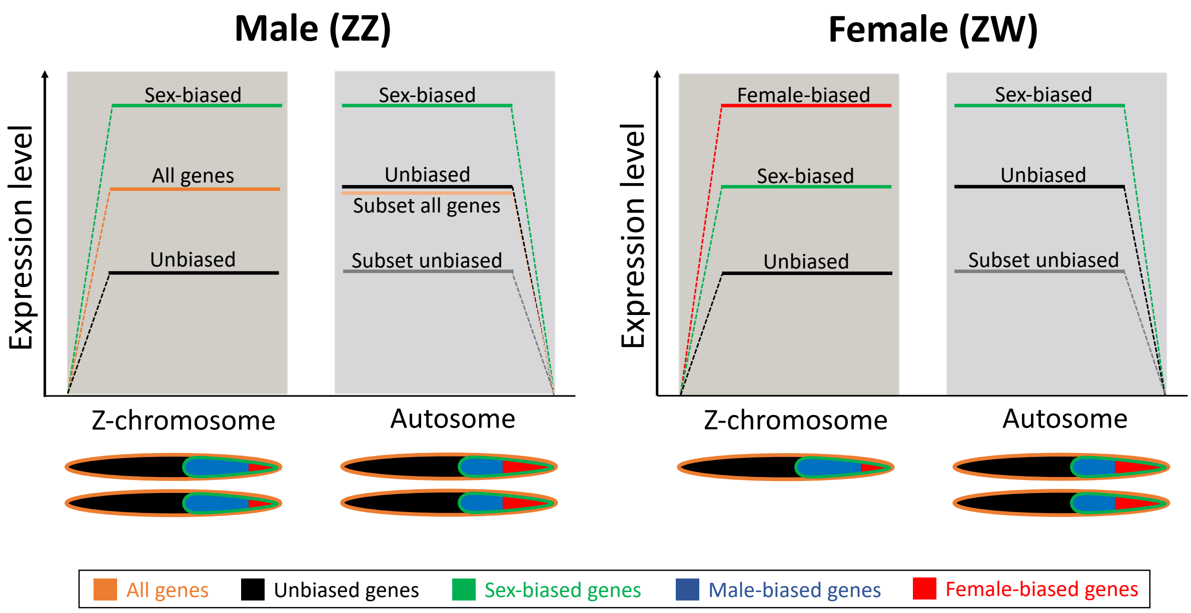

**B**

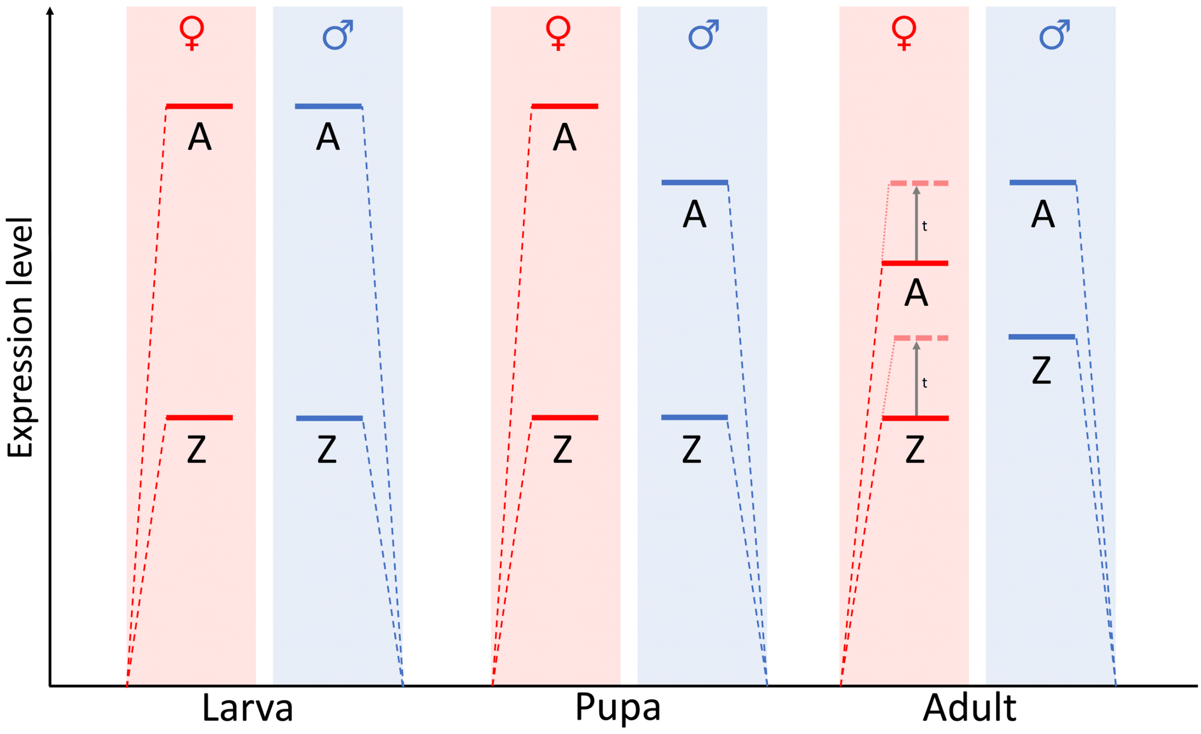

**Table S1.**

Summary of the read mapping results for individual samples. Total number of reads mapped (# reads) and percentages of uniquely mapped reads (% unique), reads mapping to multiple locations (% multi) and unmapped (% non) are given.

| Group | Sample | # reads | % unique | % multi | % non |
|---|---|---|---|---|---|
| **Larva (♂)** | S48 | 15,748,980 | 88.05 | 4.58 | 7.31 |
| | S51 | 18,127,981 | 83.35 | 5.18 | 11.47 |
| | S55 | 17,393,825 | 80.45 | 6.90 | 12.65 |
| **Larva (♀)** | S49 | 17,667,409 | 85.70 | 4.92 | 9.38 |
| | S52 | 18,384,280 | 83.61 | 5.05 | 11.34 |
| | S56 | 17,192,274 | 82.08 | 5.04 | 12.89 |
| **Pupa (♂)** | S34 | 12,956,245 | 84.98 | 3.50 | 11.52 |
| | S36 | 14,481,936 | 81.45 | 9.99 | 8.56 |
| | S57 | 18,883,750 | 86.99 | 5.34 | 7.67 |
| **Pupa (♀)** | S35 | 5,135,581 | 75.48 | 4.93 | 19.59 |
| | S53 | 16,984,253 | 84.39 | 7.57 | 8.03 |
| | S58 | 18,388,700 | 89.66 | 2.64 | 7.70 |
| **Adult (♂)** | S60 | 18,839,916 | 86.43 | 4.84 | 8.73 |
| | S61 | 18,212,301 | 87.09 | 5.36 | 7.56 |
| | S62 | 19,123,979 | 83.53 | 3.61 | 12.86 |
| **Adult (♀)** | S10 | 21,472,027 | 85.84 | 3.34 | 10.82 |
| | S38 | 61,334,814 | 80.68 | 5.01 | 14.32 |
| | S59 | 15,845,867 | 86.29 | 3.56 | 10.15 |

**Table S2.**

Number of genes expressed in males and females in each developmental stage, assigned as autosomal (all autosomes), Z-linked and linked to a specific autosome. The number of genes expressed in both sexes (FPKM > 0 in both males and females) is also shown.

| | **# genes** | | | **Autosomal** | | | **Z-linked** | | | **Specific autosome** | | |
|---|---|---|---|---|---|---|---|---|---|---|---|---|
| Total | 15,598 | | | 13,442 | | | 405 | | | 7,617 | | |
| | Females | Males | Both | Females | Males | Both | Females | Males | Both | Females | Males | Both |
| Larva | 10,593 | 10,758 | 10,080 | 10,313 | 10,455 | 9,816 | 280 | 303 | 264 | 5,856 | 5,931 | 5,569 |
| Pupa | 10,567 | 10,545 | 9,683 | 10,290 | 10,245 | 9,432 | 277 | 300 | 251 | 5,837 | 5,820 | 5,345 |
| Adult | 10,552 | 11,514 | 10,036 | 10,292 | 11,179 | 9,792 | 260 | 335 | 244 | 5,820 | 6,382 | 5,556 |

**Table S3.**

Significance levels of the Mann-Whitney *U*-tests for (A) differences in expression levels between female (F) and male (M), and between autosomal (A) and Z-chromosome linked (Z) genes, across the three developmental stages; (B) differences in expression levels across developmental stages, computed separately for each sex and chromosomal category. Only genes with FPKM > 0 in both sexes were included when computing M:F ratios.

**A**

| | A ≠ Z | | F ≠ M | |
|---|---|---|---|---|
| **Stage** | **Female** | **Male** | **Autosomal** | **Z-linked** |
| Larva | 3.6E-10 | 1.2E-12 | 0.092 | 0.625 |
| Pupa | 6.1E-08 | 1.1E-07 | 0.062 | 0.770 |
| Adult | 7.9E-09 | 0.002 | 4.2E-09 | 0.001 |

**B**

| | Female | | Male | |
|---|---|---|---|---|
| **Contrast** | **A** | **Z** | **A** | **Z** |
| Larva vs pupa | 4.6E-06 | 0.410 | 8.6E-04 | 0.577 |
| Pupa vs adult | 5.2E-03 | 0.829 | 7.8E-10 | 5.5E-03 |
| Larva vs adult | 0.110 | 0.578 | 3.7E-03 | 5.1E-04 |

**Table S4.**

Results from the Dunn's test (p-values), comparing gene expression levels between different chromosomes. Analyses were carried out separately for each sex and developmental stage. Zero-expression genes were removed separately for males and females. Significant p-values are shown in bold.

| | Larva | | Pupa | | Adult | |
|---|---|---|---|---|---|---|
| comparison | female | male | female | male | female | male |
| Z - 1 | **6.10E-05** | **3.58E-07** | **2.07E-06** | **1.01E-05** | **1.14E-07** | **1.67E-03** |
| Z - 2 | **2.29E-07** | **1.46E-06** | **1.54E-03** | **7.87E-03** | **1.93E-04** | 0.11 |
| Z - 3 | **2.54E-06** | **1.26E-05** | **3.38E-04** | **3.40E-03** | **7.13E-04** | 0.07 |
| Z - 4 | **2.19E-03** | **4.30E-05** | **9.79E-03** | **6.82E-03** | **1.69E-03** | 0.40 |
| Z - 5 | **9.86E-05** | **1.86E-05** | **1.44E-03** | **2.62E-03** | **1.03E-03** | 0.12 |
| Z - 6 | **3.86E-07** | **6.63E-09** | **3.25E-04** | **5.85E-03** | **1.27E-04** | **4.31E-02** |
| Z - 7 | **3.41E-08** | **9.45E-09** | **5.35E-09** | **5.87E-09** | **3.31E-11** | **6.05E-04** |
| Z - 8 | **2.91E-06** | **4.76E-07** | **2.37E-05** | **1.77E-04** | **1.53E-06** | 0.06 |
| Z - 9 | **1.17E-03** | **8.20E-05** | **1.32E-02** | **1.73E-02** | **2.40E-02** | **4.30E-02** |
| Z - 10 | **2.77E-10** | **3.95E-10** | **1.78E-08** | **7.51E-10** | **1.36E-08** | **1.70E-05** |
| Z - 11 | **1.20E-09** | **3.29E-11** | **1.05E-07** | **1.27E-05** | **3.77E-08** | **4.03E-03** |
| Z - 12 | **7.13E-05** | **1.57E-06** | **1.37E-03** | **1.06E-03** | **6.90E-06** | **2.41E-02** |
| Z - 13 | **6.81E-07** | **2.62E-08** | **3.63E-05** | **1.78E-05** | **6.86E-07** | **7.31E-04** |
| Z - 14 | **2.00E-08** | **4.06E-09** | **5.08E-08** | **1.98E-06** | **4.96E-08** | 0.06 |
| Z - 15 | **4.84E-04** | **6.56E-04** | **7.85E-06** | **9.62E-07** | **5.97E-04** | **6.11E-03** |
| Z - 16 | **2.16E-04** | **8.07E-07** | **7.43E-06** | **4.20E-05** | **1.16E-04** | **2.01E-03** |
| Z - 17 | **2.39E-09** | **1.43E-11** | **1.13E-05** | **3.69E-06** | **1.41E-06** | **1.17E-02** |
| Z - 18 | **2.61E-06** | **3.92E-07** | **2.23E-04** | **9.68E-04** | **2.95E-05** | 0.07 |
| Z - 19 | **2.83E-05** | **1.33E-04** | **5.55E-05** | **1.61E-03** | **1.32E-04** | 0.21 |
| Z - 20 | **3.06E-05** | **4.02E-07** | **5.69E-04** | **6.77E-03** | **5.14E-05** | 0.23 |
| 1 - 2 | **1.89E-02** | 0.21 | 0.22 | 0.15 | 0.25 | 0.14 |
| 1 - 3 | 0.08 | 0.48 | 0.29 | 0.17 | 0.09 | 0.14 |
| 1 - 4 | 0.33 | 0.40 | **4.78E-02** | 0.12 | 0.06 | **1.08E-02** |
| 1 - 5 | 0.29 | 0.49 | 0.18 | 0.23 | 0.10 | 0.10 |
| 1 - 6 | 0.08 | 0.15 | 0.16 | 0.06 | 0.08 | 0.14 |
| 1 - 7 | **2.62E-02** | 0.14 | 0.07 | **3.89E-02** | **3.89E-02** | 0.30 |
| 1 - 8 | 0.07 | 0.19 | 0.38 | 0.45 | 0.34 | 0.19 |
| 1 - 9 | 0.18 | 0.15 | 0.36 | 0.41 | 0.16 | 0.46 |
| 1 - 10 | **5.04E-03** | 0.10 | 0.15 | **2.82E-02** | 0.31 | 0.10 |
| 1 - 11 | **8.16E-03** | **3.36E-02** | 0.21 | 0.46 | 0.35 | 0.43 |
| 1 - 12 | 0.45 | 0.46 | 0.07 | 0.14 | 0.25 | 0.19 |
| 1 - 13 | 0.17 | 0.37 | 0.20 | 0.39 | 0.29 | 0.44 |
| 1 - 14 | **2.19E-02** | 0.12 | 0.15 | 0.27 | 0.31 | 0.11 |
| 1 - 15 | 0.38 | 0.23 | 0.21 | 0.07 | 0.20 | 0.41 |
| 1 - 16 | 0.32 | 0.18 | 0.25 | 0.28 | 0.31 | 0.27 |
| 1 - 17 | **1.90E-02** | **4.82E-02** | 0.31 | 0.44 | 0.25 | 0.23 |
| 1 - 18 | 0.24 | 0.47 | 0.11 | 0.10 | 0.10 | 0.06 |
| 1 - 19 | 0.49 | **4.57E-02** | 0.15 | 0.05 | **2.46E-02** | **8.34E-03** |
| 1 - 20 | 0.50 | 0.39 | **4.79E-02** | **1.74E-02** | **4.78E-02** | **7.74E-03** |

| | | | | | | |
|---|---|---|---|---|---|---|
| 2 - 3 | 0.25 | 0.25 | 0.42 | 0.45 | 0.30 | 0.46 |
| 2 - 4 | **1.33E-02** | 0.18 | 0.24 | 0.48 | 0.23 | 0.18 |
| 2 - 5 | 0.09 | 0.25 | 0.46 | 0.39 | 0.30 | 0.45 |
| 2 - 6 | 0.18 | 0.47 | 0.47 | 0.39 | 0.32 | 0.43 |
| 2 - 7 | 0.34 | 0.46 | **2.85E-02** | **7.78E-03** | **1.91E-02** | 0.07 |
| 2 - 8 | 0.28 | 0.48 | 0.18 | 0.16 | 0.17 | 0.42 |
| 2 - 9 | 0.31 | 0.34 | 0.44 | 0.33 | 0.31 | 0.22 |
| 2 - 10 | 0.48 | 0.43 | 0.06 | **5.51E-03** | 0.15 | **1.89E-02** |
| 2 - 11 | 0.45 | 0.25 | 0.09 | 0.14 | 0.17 | 0.18 |
| 2 - 12 | **2.66E-02** | 0.20 | 0.32 | 0.44 | 0.46 | 0.36 |
| 2 - 13 | 0.08 | 0.29 | 0.45 | 0.20 | 0.40 | 0.11 |
| 2 - 14 | 0.36 | 0.43 | 0.06 | 0.07 | 0.15 | 0.46 |
| 2 - 15 | 0.07 | 0.10 | 0.09 | **1.60E-02** | 0.44 | 0.14 |
| 2 - 16 | 0.09 | 0.46 | 0.11 | 0.09 | 0.45 | 0.08 |
| 2 - 17 | 0.30 | 0.32 | 0.35 | 0.12 | 0.43 | 0.30 |
| 2 - 18 | 0.06 | 0.20 | 0.42 | 0.48 | 0.37 | 0.46 |
| 2 - 19 | **1.52E-02** | **1.78E-02** | 0.49 | 0.43 | 0.21 | 0.25 |
| 2 - 20 | **1.58E-02** | 0.15 | 0.31 | 0.29 | 0.28 | 0.24 |
| 3 - 4 | 0.05 | 0.39 | 0.17 | 0.42 | 0.41 | 0.14 |
| 3 - 5 | 0.24 | 0.49 | 0.37 | 0.44 | 0.50 | 0.41 |
| 3 - 6 | 0.43 | 0.20 | 0.38 | 0.33 | 0.45 | 0.47 |
| 3 - 7 | 0.37 | 0.20 | **3.63E-02** | **8.07E-03** | **2.52E-03** | 0.07 |
| 3 - 8 | 0.47 | 0.23 | 0.23 | 0.18 | 0.06 | 0.45 |
| 3 - 9 | 0.50 | 0.18 | 0.50 | 0.36 | 0.45 | 0.23 |
| 3 - 10 | 0.23 | 0.16 | 0.08 | **5.53E-03** | **4.00E-02** | **1.44E-02** |
| 3 - 11 | 0.26 | 0.07 | 0.11 | 0.16 | 0.05 | 0.18 |
| 3 - 12 | 0.10 | 0.45 | 0.23 | 0.50 | 0.23 | 0.39 |
| 3 - 13 | 0.26 | 0.41 | 0.45 | 0.23 | 0.18 | 0.11 |
| 3 - 14 | 0.35 | 0.17 | 0.08 | 0.08 | **4.36E-02** | 0.49 |
| 3 - 15 | 0.19 | 0.25 | 0.12 | **1.76E-02** | 0.37 | 0.14 |
| 3 - 16 | 0.22 | 0.22 | 0.14 | 0.10 | 0.25 | 0.08 |
| 3 - 17 | 0.39 | 0.09 | 0.44 | 0.14 | 0.21 | 0.32 |
| 3 - 18 | 0.20 | 0.45 | 0.32 | 0.45 | 0.40 | 0.41 |
| 3 - 19 | 0.07 | 0.08 | 0.39 | 0.36 | 0.41 | 0.19 |
| 3 - 20 | 0.07 | 0.39 | 0.21 | 0.22 | 0.49 | 0.18 |

| | | | | | | |
|---|---|---|---|---|---|---|
| 4 - 5 | 0.19 | 0.41 | 0.26 | 0.37 | 0.42 | 0.20 |
| 4 - 6 | 0.05 | 0.13 | 0.23 | 0.41 | 0.36 | 0.10 |
| 4 - 7 | **1.88E-02** | 0.13 | **2.27E-03** | **4.64E-03** | **1.26E-03** | **4.32E-03** |
| 4 - 8 | **4.79E-02** | 0.16 | **4.61E-02** | 0.14 | **4.24E-02** | 0.12 |
| 4 - 9 | 0.13 | 0.13 | 0.25 | 0.32 | 0.49 | 0.07 |
| 4 - 10 | **4.64E-03** | 0.10 | **6.07E-03** | **3.05E-03** | **2.37E-02** | **3.73E-04** |
| 4 - 11 | **6.87E-03** | **3.70E-02** | **1.10E-02** | 0.11 | **3.08E-02** | **1.87E-02** |
| 4 - 12 | 0.30 | 0.44 | 0.37 | 0.41 | 0.17 | 0.07 |
| 4 - 13 | 0.11 | 0.29 | 0.16 | 0.17 | 0.12 | **6.40E-03** |
| 4 - 14 | **1.60E-02** | 0.11 | **6.86E-03** | 0.05 | **2.66E-02** | 0.12 |
| 4 - 15 | 0.26 | 0.33 | **1.95E-02** | **1.14E-02** | 0.30 | **1.86E-02** |
| 4 - 16 | 0.22 | 0.15 | **2.33E-02** | 0.07 | 0.19 | **7.66E-03** |
| 4 - 17 | **1.49E-02** | 0.05 | 0.10 | 0.10 | 0.14 | **4.40E-02** |
| 4 - 18 | 0.15 | 0.42 | 0.26 | 0.45 | 0.30 | 0.15 |
| 4 - 19 | 0.33 | 0.13 | 0.19 | 0.46 | 0.48 | 0.34 |
| 4 - 20 | 0.33 | 0.49 | 0.36 | 0.30 | 0.39 | 0.36 |
| 5 - 6 | 0.27 | 0.19 | 0.49 | 0.28 | 0.45 | 0.37 |
| 5 - 7 | 0.14 | 0.19 | **1.79E-02** | **1.48E-02** | **3.28E-03** | **4.98E-02** |
| 5 - 8 | 0.21 | 0.22 | 0.15 | 0.23 | 0.07 | 0.37 |
| 5 - 9 | 0.31 | 0.17 | 0.42 | 0.41 | 0.46 | 0.19 |
| 5 - 10 | 0.06 | 0.16 | **4.05E-02** | **1.09E-02** | **4.50E-02** | **1.04E-02** |
| 5 - 11 | 0.07 | 0.07 | 0.06 | 0.21 | 0.06 | 0.14 |
| 5 - 12 | 0.33 | 0.46 | 0.36 | 0.44 | 0.24 | 0.30 |
| 5 - 13 | 0.42 | 0.40 | 0.40 | 0.29 | 0.18 | 0.08 |
| 5 - 14 | 0.12 | 0.17 | **4.19E-02** | 0.11 | **4.84E-02** | 0.41 |
| 5 - 15 | 0.43 | 0.26 | 0.07 | **2.73E-02** | 0.37 | 0.11 |
| 5 - 16 | 0.48 | 0.21 | 0.09 | 0.13 | 0.26 | 0.06 |
| 5 - 17 | 0.13 | 0.09 | 0.30 | 0.19 | 0.21 | 0.24 |
| 5 - 18 | 0.49 | 0.47 | 0.47 | 0.39 | 0.40 | 0.48 |
| 5 - 19 | 0.28 | 0.09 | 0.46 | 0.30 | 0.42 | 0.28 |
| 5 - 20 | 0.28 | 0.40 | 0.34 | 0.18 | 0.49 | 0.27 |
| 6 - 7 | 0.29 | 0.49 | **9.93E-03** | **8.40E-04** | **1.41E-03** | 0.06 |
| 6 - 8 | 0.40 | 0.50 | 0.13 | 0.08 | 0.06 | 0.48 |
| 6 - 9 | 0.46 | 0.34 | 0.42 | 0.26 | 0.42 | 0.24 |
| 6 - 10 | 0.15 | 0.45 | **2.57E-02** | **4.17E-04** | **3.37E-02** | **1.08E-02** |
| 6 - 11 | 0.18 | 0.24 | **4.33E-02** | 0.05 | **4.45E-02** | 0.19 |
| 6 - 12 | 0.11 | 0.13 | 0.32 | 0.31 | 0.25 | 0.41 |
| 6 - 13 | 0.30 | 0.22 | 0.40 | 0.09 | 0.18 | 0.10 |
| 6 - 14 | 0.27 | 0.45 | **2.79E-02** | **1.99E-02** | **3.83E-02** | 0.46 |
| 6 - 15 | 0.22 | 0.06 | 0.06 | **3.89E-03** | 0.40 | 0.14 |
| 6 - 16 | 0.25 | 0.48 | 0.07 | **3.62E-02** | 0.27 | 0.07 |
| 6 - 17 | 0.30 | 0.32 | 0.29 | **4.20E-02** | 0.22 | 0.34 |
| 6 - 18 | 0.23 | 0.13 | 0.45 | 0.35 | 0.44 | 0.37 |
| 6 - 19 | 0.07 | **3.65E-03** | 0.47 | 0.44 | 0.35 | 0.14 |
| 6 - 20 | 0.07 | 0.09 | 0.31 | 0.37 | 0.45 | 0.13 |

| | | | | | | |
|---|---|---|---|---|---|---|
| 7 - 8 | 0.41 | 0.49 | 0.18 | 0.09 | 0.15 | 0.10 |
| 7 - 9 | 0.41 | 0.35 | 0.12 | 0.11 | **2.50E-02** | 0.42 |
| 7 - 10 | 0.32 | 0.46 | 0.31 | 0.48 | 0.10 | 0.25 |
| 7 - 11 | 0.37 | 0.25 | 0.25 | 0.05 | 0.09 | 0.25 |
| 7 - 12 | **3.89E-02** | 0.13 | **2.39E-03** | **3.55E-03** | **9.68E-03** | 0.09 |
| 7 - 13 | 0.13 | 0.22 | **1.08E-02** | **1.95E-02** | **1.00E-02** | 0.35 |
| 7 - 14 | 0.48 | 0.46 | 0.33 | 0.14 | 0.12 | 0.05 |
| 7 - 15 | 0.11 | 0.06 | 0.36 | 0.48 | **1.49E-02** | 0.42 |
| 7 - 16 | 0.13 | 0.49 | 0.30 | 0.19 | **2.84E-02** | 0.44 |
| 7 - 17 | 0.47 | 0.33 | **2.51E-02** | **4.88E-02** | **7.67E-03** | 0.11 |
| 7 - 18 | 0.10 | 0.13 | **4.31E-03** | **1.48E-03** | **1.37E-03** | **2.62E-02** |
| 7 - 19 | **1.99E-02** | **3.86E-03** | **5.99E-03** | **4.31E-04** | **1.15E-04** | **3.01E-03** |
| 7 - 20 | **2.09E-02** | 0.08 | **1.06E-03** | **7.03E-05** | **3.44E-04** | **2.80E-03** |
| 8 - 9 | 0.47 | 0.35 | 0.30 | 0.38 | 0.12 | 0.26 |
| 8 - 10 | 0.27 | 0.45 | 0.30 | 0.08 | 0.49 | **2.82E-02** |
| 8 - 11 | 0.30 | 0.27 | 0.36 | 0.48 | 0.46 | 0.24 |
| 8 - 12 | 0.09 | 0.17 | 0.06 | 0.16 | 0.17 | 0.44 |
| 8 - 13 | 0.23 | 0.26 | 0.16 | 0.36 | 0.19 | 0.15 |
| 8 - 14 | 0.39 | 0.45 | 0.29 | 0.36 | 0.50 | 0.44 |
| 8 - 15 | 0.18 | 0.08 | 0.32 | 0.12 | 0.14 | 0.18 |
| 8 - 16 | 0.21 | 0.48 | 0.37 | 0.35 | 0.21 | 0.10 |
| 8 - 17 | 0.43 | 0.34 | 0.24 | 0.49 | 0.17 | 0.38 |
| 8 - 18 | 0.18 | 0.17 | 0.10 | 0.13 | 0.07 | 0.37 |
| 8 - 19 | 0.06 | **1.22E-02** | 0.13 | 0.08 | **2.36E-02** | 0.17 |
| 8 - 20 | 0.07 | 0.13 | 0.05 | **3.73E-02** | **4.00E-02** | 0.16 |
| 9 -10 | 0.31 | 0.37 | 0.18 | 0.10 | 0.11 | 0.27 |
| 9 - 11 | 0.33 | 0.50 | 0.21 | 0.39 | 0.12 | 0.42 |
| 9 - 12 | 0.20 | 0.14 | 0.32 | 0.36 | 0.28 | 0.28 |
| 9 - 13 | 0.34 | 0.20 | 0.47 | 0.46 | 0.24 | 0.49 |
| 9 - 14 | 0.40 | 0.37 | 0.17 | 0.28 | 0.11 | 0.22 |
| 9 - 15 | 0.27 | 0.08 | 0.20 | 0.12 | 0.36 | 0.48 |
| 9 - 16 | 0.30 | 0.37 | 0.22 | 0.28 | 0.28 | 0.38 |
| 9 - 17 | 0.43 | 0.44 | 0.46 | 0.38 | 0.26 | 0.31 |
| 9 - 18 | 0.30 | 0.14 | 0.39 | 0.33 | 0.39 | 0.18 |
| 9 - 19 | 0.18 | **2.79E-02** | 0.43 | 0.28 | 0.50 | 0.09 |
| 9 - 20 | 0.18 | 0.12 | 0.31 | 0.20 | 0.45 | 0.09 |

| | | | | | | |
|---|---|---|---|---|---|---|
| 10 - 11 | 0.46 | 0.27 | 0.42 | **4.09E-02** | 0.46 | 0.08 |
| 10 - 12 | **9.28E-03** | 0.10 | **6.91E-03** | **2.03E-03** | 0.13 | **1.75E-02** |
| 10 - 13 | **4.32E-02** | 0.17 | **2.95E-02** | **1.27E-02** | 0.15 | 0.12 |
| 10 - 14 | 0.35 | 0.50 | 0.48 | 0.12 | 0.49 | **8.12E-03** |
| 10 - 15 | **4.91E-02** | **4.38E-02** | 0.49 | 0.49 | 0.12 | 0.22 |
| 10 - 16 | 0.06 | 0.48 | 0.45 | 0.18 | 0.19 | 0.35 |
| 10 - 17 | 0.28 | 0.36 | 0.06 | **3.60E-02** | 0.12 | **2.10E-02** |
| 10 - 18 | **2.86E-02** | 0.09 | **1.24E-02** | **7.12E-04** | **3.80E-02** | **2.54E-03** |
| 10 - 19 | **3.04E-03** | **1.26E-03** | **1.73E-02** | **1.68E-04** | **7.13E-03** | **9.86E-05** |
| 10 - 20 | **3.32E-03** | 0.05 | **3.36E-03** | **2.14E-05** | **1.57E-02** | **9.29E-05** |
| 11 - 12 | **1.40E-02** | **3.22E-02** | **1.35E-02** | 0.13 | 0.15 | 0.25 |
| 11 - 13 | 0.06 | 0.06 | 0.05 | 0.36 | 0.18 | 0.37 |
| 11 - 14 | 0.39 | 0.28 | 0.41 | 0.31 | 0.45 | 0.16 |
| 11 - 15 | 0.06 | **1.55E-02** | 0.43 | 0.08 | 0.13 | 0.36 |
| 11 - 16 | 0.07 | 0.30 | 0.49 | 0.32 | 0.21 | 0.23 |
| 11 - 17 | 0.32 | 0.39 | 0.10 | 0.48 | 0.15 | 0.30 |
| 11 - 18 | **4.06E-02** | **2.76E-02** | **2.39E-02** | 0.09 | 0.05 | 0.10 |
| 11 - 19 | **5.32E-03** | **1.92E-04** | **3.30E-02** | 0.05 | **1.09E-02** | **1.79E-02** |
| 11 - 20 | **5.74E-03** | **1.42E-02** | **7.73E-03** | **1.67E-02** | **2.27E-02** | **1.67E-02** |
| 12 - 13 | 0.21 | 0.33 | 0.23 | 0.20 | 0.43 | 0.14 |
| 12 - 14 | **3.32E-02** | 0.11 | **8.15E-03** | 0.06 | 0.13 | 0.37 |
| 12 - 15 | 0.41 | 0.26 | **2.64E-02** | **1.14E-02** | 0.39 | 0.18 |
| 12 - 16 | 0.36 | 0.16 | **3.16E-02** | 0.08 | 0.48 | 0.10 |
| 12 - 17 | **3.11E-02** | **4.59E-02** | 0.14 | 0.11 | 0.47 | 0.43 |
| 12 - 18 | 0.28 | 0.49 | 0.36 | 0.44 | 0.29 | 0.28 |
| 12 - 19 | 0.44 | 0.07 | 0.28 | 0.34 | 0.13 | 0.09 |
| 12 - 20 | 0.45 | 0.43 | 0.50 | 0.19 | 0.19 | 0.08 |
| 13 - 14 | 0.12 | 0.19 | **3.26E-02** | 0.19 | 0.15 | 0.08 |
| 13 - 15 | 0.35 | 0.16 | 0.07 | **4.23E-02** | 0.33 | 0.46 |
| 13 - 16 | 0.40 | 0.25 | 0.09 | 0.21 | 0.46 | 0.31 |
| 13 - 17 | 0.12 | 0.09 | 0.36 | 0.33 | 0.45 | 0.18 |
| 13 - 18 | 0.41 | 0.34 | 0.34 | 0.15 | 0.21 | **4.08E-02** |
| 13 - 19 | 0.15 | **1.69E-02** | 0.42 | 0.08 | 0.07 | **3.94E-03** |
| 13 - 20 | 0.16 | 0.25 | 0.20 | **2.85E-02** | 0.12 | **3.66E-03** |
| 14 - 15 | 0.10 | 0.05 | 0.50 | 0.17 | 0.12 | 0.12 |
| 14 - 16 | 0.12 | 0.48 | 0.44 | 0.47 | 0.19 | 0.06 |
| 14 - 17 | 0.44 | 0.37 | 0.07 | 0.31 | 0.13 | 0.30 |
| 14 - 18 | 0.08 | 0.11 | **1.45E-02** | **3.50E-02** | **4.35E-02** | 0.41 |
| 14 - 19 | **1.63E-02** | **2.59E-03** | **2.00E-02** | **1.64E-02** | **9.67E-03** | 0.17 |
| 14 - 20 | **1.72E-02** | 0.07 | **4.38E-03** | **4.51E-03** | **1.97E-02** | 0.16 |

| | | | | | | |
|---|---|---|---|---|---|---|
| 15 - 16 | 0.45 | 0.08 | 0.45 | 0.21 | 0.38 | 0.37 |
| 15 - 17 | 0.11 | **2.16E-02** | 0.12 | 0.08 | 0.36 | 0.21 |
| 15 - 18 | 0.41 | 0.25 | **4.22E-02** | **6.74E-03** | 0.44 | 0.08 |
| 15 - 19 | 0.37 | 0.30 | 0.05 | **3.14E-03** | 0.28 | **2.13E-02** |
| 15 - 20 | 0.37 | 0.30 | **2.03E-02** | **9.28E-04** | 0.36 | **2.00E-02** |
| 16 - 17 | 0.13 | 0.37 | 0.14 | 0.32 | 0.50 | 0.12 |
| 16 - 18 | 0.47 | 0.16 | 0.05 | 0.06 | 0.31 | **3.75E-02** |
| 16 - 19 | 0.31 | **1.28E-02** | 0.07 | **3.49E-02** | 0.17 | **7.76E-03** |
| 16 - 20 | 0.31 | 0.12 | **2.42E-02** | **1.39E-02** | 0.23 | **7.25E-03** |
| 17 - 18 | 0.09 | **3.96E-02** | 0.23 | 0.07 | 0.25 | 0.21 |
| 17 - 19 | **1.30E-02** | **2.24E-04** | 0.29 | **3.65E-02** | 0.09 | **4.89E-02** |
| 17 - 20 | **1.39E-02** | **2.02E-02** | 0.12 | **1.03E-02** | 0.16 | **4.55E-02** |
| 18 - 19 | 0.22 | 0.05 | 0.41 | 0.39 | 0.27 | 0.22 |
| 18 - 20 | 0.22 | 0.42 | 0.34 | 0.21 | 0.38 | 0.20 |
| 19 - 20 | 0.50 | 0.07 | 0.25 | 0.30 | 0.38 | 0.48 |

**Table S5.**

Number of significantly sex-biased genes inferred from the differential expression analysis using DEseq2. P-values are given for each developmental stage ($\chi^2$-test as implemented in R).

| Stage | # genes after filtering (*de novo* assembly) | Female biased | Male biased | p-value |
|---|---|---|---|---|
| Larva | 11,141 | 68 | 139 | $8.0*10^{-7}$ |
| Pupa | 11,422 | 90 | 389 | $< 2.2*10^{-16}$ |
| Adult | 12,272 | 1,836 | 1,942 | $8.5*10^{-2}$ |

**Table S6.**

Summary of the non-random genomic distribution of sex-biased genes (SBG) for both females and males across chromosome classes (A and Z, respectively) as assessed by applying Fisher's exact tests (two-sided) for counts of observed (obs) and expected (exp) genes. The total number of genes in each category are given within parentheses. Note that some sex-biased genes could not be assigned to a chromosome (A or Z) and were therefore not included in the test. Significance levels = * < 0.05; ** < 0.01; *** < 0.001.

| | Total SBG | A obs. | A exp. | Z obs. | Z exp. | p-value |
|---|---|---|---|---|---|---|
| Female | | | | | | |
| Larva | 68 | 0.95 (59) | 0.97 (9,809) | 0.05 (3) | 0.03 (262) | 0.22 |
| Pupa | 90 | 0.95 (75) | 0.97 (10,061) | 0.05 (4) | 0.03 (279) | 0.17 |
| Adult | 1,836 | 0.99 (1,665) | 0.97 (10,773) | 0.01 (22) | 0.03 (319) | $6.5*10^{-5***}$ |
| Male | | | | | | |
| Larva | 139 | 0.94 (120) | 0.97 (9,809) | 0.06 (8) | 0.03 (262) | $0.020^{*}$ |
| Pupa | 389 | 0.94 (325) | 0.97 (10,061) | 0.06 (21) | 0.03 (279) | $7.9*10^{-4***}$ |
| Adult | 1,942 | 0.95 (1,657) | 0.97 (10,773) | 0.05 (80) | 0.03 (319) | $2.5*10^{-4***}$ |

**Table S7.**

Significance levels of the Mann-Whitney *U*-tests for: (A) differences in expression levels, after excluding sex-biased genes, between female (F) and male (M), and between autosomal (A) and Z-chromosome linked (Z) genes, across the three developmental stages; (B) differences in expression levels across developmental stages, after excluding sex-biased genes, computed separately for each sex and chromosomal category; (C) differences in expression levels after and before removing sex-biased genes, computed separately for each sex, developmental stage, and chromosomal category. Only genes with FPKM > 0 in both sexes were included when computing M:F ratios.

**A**

| | A ≠ Z | | F ≠ M | |
|---|---|---|---|---|
| **Stage** | **Female** | **Male** | **Autosomal** | **Z-linked** |
| Larva | 4.9E-10 | 1.7E-13 | 0.447 | 0.948 |
| Pupa | 1.2E-07 | 9.4E-10 | 6.9E-05 | 0.692 |
| Adult | 4.1E-04 | 5.9E-05 | 5.1E-08 | 0.080 |

**B**

| | Female | | Male | |
|---|---|---|---|---|
| **Contrast** | **A** | **Z** | **A** | **Z** |
| Larva vs pupa | 1.1E-04 | 0.573 | 4.4E-06 | 0.861 |
| Pupa vs adult | 2.7E-08 | 0.984 | 2.2E-03 | 0.490 |
| Larva vs adult | 1.8E-21 | 0.574 | 2.1E-13 | 0.532 |

**C**

| | Before ≠ After removing SBG Female | | Before ≠ After removing SBG Male | |
|---|---|---|---|---|
| **Stage** | **A** | **Z** | **A** | **Z** |
| Larva | 0.986 | 0.983 | 0.385 | 0.680 |
| Pupa | 0.507 | 0.857 | 3.9E-02 | 0.273 |
| Adult | 7.3E-14 | 0.982 | 5.4E-27 | 2.5E-03 |

**Table S8.**

Median and mean expression levels for autosomal (A) and Z-linked (Z) genes after excluding sex-biased genes, observed in females and males, for different developmental stages. Only genes with FPKM > 0 in both sexes were included.

| Stage | Median FPKM | | | | Mean FPKM | | | |
|---|---|---|---|---|---|---|---|---|
| | Female | | Male | | Female | | Male | |
| | A | Z | A | Z | A | Z | A | Z |
| Larva | 82 | 38 | 86 | 35 | 4,510 | 912 | 3,970 | 857 |
| Pupa | 82 | 38 | 71 | 39 | 2,955 | 892 | 5,991 | 668 |
| Adult | 61 | 37 | 73 | 47 | 1,244 | 798 | 1,758 | 1,478 |

**Table S9.**

Quartile based analysis of Z-linked expression, before excluding sex-biased genes. For each quartile, the Mann-Whitney *U*-test p-value and number of genes analyzed (N) are shown. Only genes with FPKM > 0 in both sexes were included. Significant p-values are shown in bold.

| | **Quartile 1** | | **Quartile 2** | | **Quartile 3** | | **Quartile 4** | |
|---|---|---|---|---|---|---|---|---|
| **Stage** | **N** | **p-value** | **N** | **p-value** | **N** | **p-value** | **N** | **p-value** |
| Larva | 66 | 0.51 | 66 | **4.2E-03** | 66 | 0.15 | 66 | 0.33 |
| Pupa | 62 | 0.59 | 63 | 0.82 | 63 | 0.55 | 63 | 0.48 |
| Adult | 61 | **4.5E-02** | 61 | **1.0E-05** | 61 | **1.8E-02** | 61 | **1.9E-03** |

**Table S10.**

Quartile based analysis of Z-linked expression after excluding sex-biased genes. For each quartile, the MW*U*-test p-value and number of genes (N) are shown. Only genes with FPKM > 0 in both sexes were included. Significant p-values are shown in bold.

| | Quartile 1 | | Quartile 2 | | Quartile 3 | | Quartile 4 | |
|---|---|---|---|---|---|---|---|---|
| **Stage** | **N** | **p-value** | **N** | **p-value** | **N** | **p-value** | **N** | **p-value** |
| Larva | 63 | 0.68 | 63 | **1.7E-02** | 63 | 0.61 | 64 | 0.67 |
| Pupa | 59 | 0.48 | 60 | 0.47 | 60 | 0.52 | 60 | 0.14 |
| Adult | 45 | 0.06 | 45 | **7.7E-03** | 45 | **1.6E-03** | 46 | 0.13 |

**Table S11.**

Number of significantly sex-biased genes inferred from the differential expression analysis using DESEQ2. Data based on *de novo* transcriptome assembly.

| Stage | # genes after filtering (*de novo* assembly) | Female biased | Male biased |
|---|---|---|---|
| Larva | 15,218 | 39 | 164 |
| Pupa | 16,104 | 115 | 498 |
| Adult | 23,264 | 2,312 | 2,653 |

**Table S12.**

Number of common sex-biased genes present in two or more developmental stages. Data based on *de novo* transcriptome assembly.

| Stages | Female biased | Male biased | Changed bias | Total |
|---|---|---|---|---|
| Larva - Pupa | 6 | 131 | 1 | 138 |
| Pupa - Adult | 25 | 463 | 12 | 500 |
| All stages | 4 | 130 | 1 | 135 |

**Table S13A.**

List of significant GO categories, based on set of male-biased significantly expressed genes detected in larvae. The number of differentially expressed genes (significant) and the total number of annotated genes associated to a GO category are shown, as well as the number of expected genes. p-values were computed using the Fisher-elim algorithm.

| | GO.ID | Term | Annotated | Significant | Expected | elim_Fisher |
|---|---|---|---|---|---|---|
| 1 | GO:0060271 | cilium assembly | 56 | 9 | 0.36 | 1.60E-06 |
| 2 | GO:0060294 | cilium movement involved in cell motility | 5 | 3 | 0.03 | 2.50E-06 |
| 3 | GO:0030317 | flagellated sperm motility | 20 | 4 | 0.13 | 7.00E-06 |
| 4 | GO:0006108 | malate metabolic process | 12 | 3 | 0.08 | 5.40E-05 |
| 5 | GO:0044458 | motile cilium assembly | 3 | 2 | 0.02 | 0.00012 |
| 6 | GO:0006099 | tricarboxylic acid cycle | 43 | 4 | 0.28 | 0.00016 |
| 7 | GO:0006919 | activation of cysteine-type endopeptidase activity | 18 | 3 | 0.12 | 0.0002 |
| 8 | GO:0035082 | axoneme assembly | 19 | 3 | 0.12 | 0.00023 |
| 9 | GO:0035096 | larval midgut cell programmed cell death | 26 | 3 | 0.17 | 0.0006 |
| 10 | GO:0035209 | pupal development | 28 | 3 | 0.18 | 0.00075 |
| 11 | GO:0003341 | cilium movement | 18 | 5 | 0.12 | 0.00269 |
| 12 | GO:0007020 | microtubule nucleation | 13 | 2 | 0.08 | 0.00308 |
| 13 | GO:0000413 | protein peptidyl-prolyl isomerization | 15 | 2 | 0.1 | 0.00411 |
| 14 | GO:0040003 | chitin-based cuticle development | 106 | 4 | 0.69 | 0.00477 |
| 15 | GO:0006457 | protein folding | 108 | 4 | 0.7 | 0.0051 |
| 16 | GO:0031670 | cellular response to nutrient | 1 | 1 | 0.01 | 0.0065 |
| 17 | GO:0050910 | detect. of stimulus involved in perception of sound | 1 | 1 | 0.01 | 0.0065 |
| 18 | GO:0007618 | mating | 73 | 3 | 0.47 | 0.01161 |
| 19 | GO:0032261 | purine nucleotide salvage | 2 | 1 | 0.01 | 0.01295 |
| 20 | GO:0032264 | IMP salvage | 2 | 1 | 0.01 | 0.01295 |
| 21 | GO:0033499 | galactose catabolic process via UDP-galactose | 2 | 1 | 0.01 | 0.01295 |
| 22 | GO:0036158 | outer dynein arm assembly | 2 | 1 | 0.01 | 0.01295 |
| 23 | GO:0035071 | salivary gland cell autophagic cell death | 85 | 3 | 0.55 | 0.01748 |
| 24 | GO:0048102 | autophagic cell death | 85 | 3 | 0.55 | 0.01748 |
| 25 | GO:0001539 | cilium or flagellum-dependent cell motility | 8 | 4 | 0.05 | 0.0181 |
| 26 | GO:0060285 | cilium-dependent cell motility | 8 | 4 | 0.05 | 0.0181 |
| 27 | GO:0035070 | salivary gland histolysis | 88 | 3 | 0.57 | 0.01916 |
| 28 | GO:0006636 | unsaturated fatty acid biosynthetic process | 3 | 1 | 0.02 | 0.01937 |
| 29 | GO:0019388 | galactose catabolic process | 3 | 1 | 0.02 | 0.01937 |
| 30 | GO:0006566 | threonine metabolic process | 4 | 1 | 0.03 | 0.02574 |
| 31 | GO:0006567 | threonine catabolic process | 4 | 1 | 0.03 | 0.02574 |
| 32 | GO:0010378 | temperature compensation of the circadian clock | 4 | 1 | 0.03 | 0.02574 |

| | | | | | | |
|---|---|---|---|---|---|---|
| 33 | GO:0010996 | response to auditory stimulus | 4 | 1 | 0.03 | 0.02574 |
| 34 | GO:0060179 | male mating behavior | 42 | 2 | 0.27 | 0.03015 |
| 35 | GO:0006228 | UTP biosynthetic process | 5 | 1 | 0.03 | 0.03208 |
| 36 | GO:0007158 | neuron cell-cell adhesion | 5 | 1 | 0.03 | 0.03208 |
| 37 | GO:0019320 | hexose catabolic process | 5 | 1 | 0.03 | 0.03208 |
| 38 | GO:0033559 | unsaturated fatty acid metabolic process | 5 | 1 | 0.03 | 0.03208 |
| 39 | GO:0043101 | purine-containing compound salvage | 5 | 1 | 0.03 | 0.03208 |
| 40 | GO:0046051 | UTP metabolic process | 5 | 1 | 0.03 | 0.03208 |
| 41 | GO:0043173 | nucleotide salvage | 6 | 1 | 0.04 | 0.03837 |
| 42 | GO:0046365 | monosaccharide catabolic process | 6 | 1 | 0.04 | 0.03837 |
| 43 | GO:0048009 | insulin-like growth factor receptor sign... | 6 | 1 | 0.04 | 0.03837 |
| 44 | GO:0006183 | GTP biosynthetic process | 7 | 1 | 0.05 | 0.04462 |
| 45 | GO:0006188 | IMP biosynthetic process | 7 | 1 | 0.05 | 0.04462 |
| 46 | GO:0035080 | heat shock-mediated polytene chromosome ... | 7 | 1 | 0.05 | 0.04462 |
| 47 | GO:0070286 | axonemal dynein complex assembly | 7 | 1 | 0.05 | 0.04462 |
| 48 | GO:0044782 | cilium organization | 65 | 10 | 0.42 | 0.04621 |
| 49 | GO:0007605 | sensory perception of sound | 57 | 3 | 0.37 | 0.04894 |

**Table S13B.**

List of significant GO categories, based on set of female-biased significantly expressed genes detected in larvae. The number of differentially expressed genes (significant) and the total number of annotated genes associated to a GO category are shown, as well as the number of expected genes. P-values were computed using the Fisher-elim algorithm.

| | GO.ID | Term | Annotated | Significant | Expected | elim_Fisher |
|---|---|---|---|---|---|---|
| 1 | GO:0015032 | storage protein import into fat body | 2 | 1 | 0 | 0.0046 |
| 2 | GO:0032211 | negative regulation of telomere maintenance | 4 | 1 | 0.01 | 0.0092 |
| 3 | GO:0051974 | negative regulation of telomerase activity | 4 | 1 | 0.01 | 0.0092 |
| 4 | GO:0044806 | G-quadruplex DNA unwinding | 5 | 1 | 0.01 | 0.0115 |
| 5 | GO:0006583 | melanin biosynthetic process from tyrosine | 7 | 1 | 0.02 | 0.0161 |
| 6 | GO:0006094 | gluconeogenesis | 9 | 1 | 0.02 | 0.0207 |
| 7 | GO:0019319 | hexose biosynthetic process | 9 | 1 | 0.02 | 0.0207 |
| 8 | GO:0046364 | monosaccharide biosynthetic process | 9 | 1 | 0.02 | 0.0207 |
| 9 | GO:0097254 | renal tubular secretion | 11 | 1 | 0.03 | 0.0252 |
| 10 | GO:0000002 | mitochondrial genome maintenance | 12 | 1 | 0.03 | 0.0275 |
| 11 | GO:0006570 | tyrosine metabolic process | 14 | 1 | 0.03 | 0.032 |
| 12 | GO:0042417 | dopamine metabolic process | 14 | 1 | 0.03 | 0.032 |
| 13 | GO:0006584 | catecholamine metabolic process | 15 | 1 | 0.03 | 0.0343 |
| 14 | GO:0009712 | catechol-containing compound metabolic process | 15 | 1 | 0.03 | 0.0343 |
| 15 | GO:0042438 | melanin biosynthetic process | 18 | 1 | 0.04 | 0.041 |
| 16 | GO:0032508 | DNA duplex unwinding | 19 | 1 | 0.04 | 0.0432 |
| 17 | GO:0003014 | renal system process | 20 | 1 | 0.05 | 0.0454 |
| 18 | GO:0050830 | defense response to Gram-positive bacterium | 20 | 1 | 0.05 | 0.0454 |
| 19 | GO:0032392 | DNA geometric change | 21 | 1 | 0.05 | 0.0477 |

**Table S13C.**

List of significant GO categories, based on set of male-biased significantly expressed genes detected in pupae. The number of differentially expressed genes (significant) and the total number of annotated genes associated to a GO category are shown, as well as the number of expected genes. P-values were computed using the Fisher-elim algorithm.

| | GO.ID | Term | Annotated | Significant | Expected | elim_Fisher |
|---|---|---|---|---|---|---|
| 1 | GO:0003341 | cilium movement | 22 | 13 | 0.48 | 3.40E-10 |
| 2 | GO:0044458 | motile cilium assembly | 5 | 5 | 0.11 | 4.50E-09 |
| 3 | GO:0060294 | cilium movement involved in cell motility | 6 | 5 | 0.13 | 2.60E-08 |
| 4 | GO:0060271 | cilium assembly | 68 | 18 | 1.47 | 1.50E-07 |
| 5 | GO:0030317 | flagellated sperm motility | 22 | 7 | 0.48 | 2.50E-07 |
| 6 | GO:0006099 | tricarboxylic acid cycle | 45 | 7 | 0.97 | 4.40E-05 |
| 7 | GO:0006108 | malate metabolic process | 12 | 4 | 0.26 | 9.10E-05 |
| 8 | GO:0003333 | amino acid transmembrane transport | 26 | 5 | 0.56 | 0.0002 |
| 9 | GO:0007605 | sensory perception of sound | 58 | 7 | 1.26 | 0.00023 |
| 10 | GO:0006919 | activation of cysteine-type endopeptidase activity | 18 | 4 | 0.39 | 0.00051 |
| 11 | GO:0035209 | pupal development | 26 | 4 | 0.56 | 0.00218 |
| 12 | GO:0035082 | axoneme assembly | 23 | 7 | 0.5 | 0.00225 |
| 13 | GO:0042073 | intraciliary transport | 13 | 3 | 0.28 | 0.00243 |
| 14 | GO:0042775 | mitoch. ATP synthesis coupled electron transport | 64 | 6 | 1.39 | 0.00248 |
| 15 | GO:0035096 | larval midgut cell programmed cell death | 27 | 4 | 0.58 | 0.00251 |
| 16 | GO:0070286 | axonemal dynein complex assembly | 10 | 4 | 0.22 | 0.00435 |
| 17 | GO:0018095 | protein polyglutamylation | 5 | 2 | 0.11 | 0.00446 |
| 18 | GO:0036158 | outer dynein arm assembly | 5 | 2 | 0.11 | 0.00446 |
| 19 | GO:0010923 | negative regulation of phosphatase activity | 8 | 2 | 0.17 | 0.01197 |
| 20 | GO:0006508 | proteolysis | 541 | 23 | 11.71 | 0.01362 |
| 21 | GO:0010921 | regulation of phosphatase activity | 9 | 2 | 0.19 | 0.01517 |
| 22 | GO:0035305 | negative regulation of dephosphorylation | 10 | 2 | 0.22 | 0.0187 |
| 23 | GO:0009165 | nucleotide biosynthetic process | 99 | 6 | 2.14 | 0.02008 |
| 24 | GO:1901293 | nucleoside phosphate biosynthetic process | 99 | 6 | 2.14 | 0.02008 |
| 25 | GO:0003351 | epithelial cilium movement | 1 | 1 | 0.02 | 0.02165 |
| 26 | GO:0007198 | adenylate cyclase-inhibiting serotonin receptor | 1 | 1 | 0.02 | 0.02165 |
| 27 | GO:0007208 | phospholipase C-activating serotonin receptor | 1 | 1 | 0.02 | 0.02165 |
| 28 | GO:0007210 | serotonin receptor signaling pathway | 1 | 1 | 0.02 | 0.02165 |
| 29 | GO:0015744 | succinate transport | 1 | 1 | 0.02 | 0.02165 |
| 30 | GO:0019805 | quinolinate biosynthetic process | 1 | 1 | 0.02 | 0.02165 |
| 31 | GO:0033233 | regulation of protein sumoylation | 1 | 1 | 0.02 | 0.02165 |
| 32 | GO:0033234 | negative regulation of protein sumoylation | 1 | 1 | 0.02 | 0.02165 |
| 33 | GO:0044745 | amino acid transmembrane import | 1 | 1 | 0.02 | 0.02165 |

| | | | | | | |
|---|---|---|---|---|---|---|
| 34 | GO:0046874 | quinolinate metabolic process | 1 | 1 | 0.02 | 0.02165 |
| 35 | GO:0050910 | detect. of stimulus involved in perception of sound | 1 | 1 | 0.02 | 0.02165 |
| 36 | GO:0060287 | epithelial cilium movement involved in d... | 1 | 1 | 0.02 | 0.02165 |
| 37 | GO:0098664 | G-protein coupled serotonin receptor sig... | 1 | 1 | 0.02 | 0.02165 |
| 38 | GO:0007007 | inner mitochondrial membrane organizatio... | 11 | 2 | 0.24 | 0.02254 |
| 39 | GO:0006753 | nucleoside phosphate metabolic process | 234 | 14 | 5.07 | 0.02617 |
| 40 | GO:0009117 | nucleotide metabolic process | 234 | 14 | 5.07 | 0.02617 |
| 41 | GO:0035303 | regulation of dephosphorylation | 12 | 2 | 0.26 | 0.02666 |
| 42 | GO:0009145 | purine nucleoside triphosphate biosynthe... | 31 | 3 | 0.67 | 0.02872 |
| 43 | GO:0009206 | purine ribonucleoside triphosphate biosy... | 31 | 3 | 0.67 | 0.02872 |
| 44 | GO:0009150 | purine ribonucleotide metabolic process | 176 | 12 | 3.81 | 0.02991 |
| 45 | GO:0007020 | microtubule nucleation | 13 | 2 | 0.28 | 0.03107 |
| 46 | GO:0006163 | purine nucleotide metabolic process | 178 | 12 | 3.85 | 0.03224 |
| 47 | GO:0006120 | mitochondrial electron transport | 33 | 3 | 0.71 | 0.03379 |
| 48 | GO:0009201 | ribonucleoside triphosphate biosynthetic... | 34 | 3 | 0.74 | 0.03649 |
| 49 | GO:0009259 | ribonucleotide metabolic process | 184 | 12 | 3.98 | 0.03993 |
| 50 | GO:0009142 | nucleoside triphosphate biosynthetic pro... | 36 | 3 | 0.78 | 0.04221 |
| 51 | GO:0006842 | tricarboxylic acid transport | 2 | 1 | 0.04 | 0.04284 |
| 52 | GO:0006848 | pyruvate transport | 2 | 1 | 0.04 | 0.04284 |
| 53 | GO:0007054 | spindle assembly involved in male meiosi... | 2 | 1 | 0.04 | 0.04284 |
| 54 | GO:0015746 | citrate transport | 2 | 1 | 0.04 | 0.04284 |
| 55 | GO:0016539 | intein-mediated protein splicing | 2 | 1 | 0.04 | 0.04284 |
| 56 | GO:0019230 | proprioception | 2 | 1 | 0.04 | 0.04284 |
| 57 | GO:0030908 | protein splicing | 2 | 1 | 0.04 | 0.04284 |
| 58 | GO:0031991 | regulation of actomyosin contractile rin... | 2 | 1 | 0.04 | 0.04284 |
| 59 | GO:0032261 | purine nucleotide salvage | 2 | 1 | 0.04 | 0.04284 |
| 60 | GO:0032264 | IMP salvage | 2 | 1 | 0.04 | 0.04284 |
| 61 | GO:0032978 | protein insertion into membrane from inn... | 2 | 1 | 0.04 | 0.04284 |
| 62 | GO:0032979 | protein insertion into mitochondrial mem... | 2 | 1 | 0.04 | 0.04284 |
| 63 | GO:0033499 | galactose catabolic process via UDP-gala... | 2 | 1 | 0.04 | 0.04284 |
| 64 | GO:0051204 | protein insertion into mitochondrial mem... | 2 | 1 | 0.04 | 0.04284 |
| 65 | GO:0000413 | protein peptidyl-prolyl isomerization | 16 | 2 | 0.35 | 0.04583 |
| 66 | GO:0009127 | purine nucleoside monophosphate biosynth... | 38 | 3 | 0.82 | 0.04835 |
| 67 | GO:0009168 | purine ribonucleoside monophosphate bios... | 38 | 3 | 0.82 | 0.04835 |

**Table S13D.**

List of significant GO categories, based on set of female-biased significantly expressed genes detected in pupae. The number of differentially expressed genes (significant) and the total number of annotated genes associated to a GO category are shown, as well as the number of expected genes. P-values were computed using the Fisher-elim algorithm.

| | GO.ID | Term | Annotated | Significant | Expected | elim_Fisher |
|---|---|---|---|---|---|---|
| 1 | GO:0040003 | chitin-based cuticle development | 121 | 8 | 0.57 | 6.10E-08 |
| 2 | GO:0007171 | transmembrane receptor protein Tyr kinase activity | 1 | 1 | 0 | 0.0047 |
| 3 | GO:0097115 | neurexin clustering | 1 | 1 | 0 | 0.0047 |
| 4 | GO:0015074 | DNA integration | 75 | 3 | 0.35 | 0.0052 |
| 5 | GO:0006694 | steroid biosynthetic process | 42 | 2 | 0.2 | 0.0166 |
| 6 | GO:0007158 | neuron cell-cell adhesion | 4 | 1 | 0.02 | 0.0188 |
| 7 | GO:0008611 | ether lipid biosynthetic process | 7 | 1 | 0.03 | 0.0327 |
| 8 | GO:0010025 | wax biosynthetic process | 7 | 1 | 0.03 | 0.0327 |
| 9 | GO:0010166 | wax metabolic process | 7 | 1 | 0.03 | 0.0327 |
| 10 | GO:0046485 | ether lipid metabolic process | 7 | 1 | 0.03 | 0.0327 |
| 11 | GO:0046504 | glycerol ether biosynthetic process | 7 | 1 | 0.03 | 0.0327 |
| 12 | GO:0097384 | cellular lipid biosynthetic process | 7 | 1 | 0.03 | 0.0327 |
| 13 | GO:1901503 | ether biosynthetic process | 7 | 1 | 0.03 | 0.0327 |
| 14 | GO:0008202 | steroid metabolic process | 62 | 2 | 0.29 | 0.0344 |
| 15 | GO:0006030 | chitin metabolic process | 64 | 2 | 0.3 | 0.0364 |
| 16 | GO:1901071 | glucosamine-containing compound metabolic process | 71 | 2 | 0.34 | 0.044 |
| 17 | GO:0006040 | amino sugar metabolic process | 73 | 2 | 0.35 | 0.0463 |
| 18 | GO:0006662 | glycerol ether metabolic process | 10 | 1 | 0.05 | 0.0463 |
| 19 | GO:0018904 | ether metabolic process | 10 | 1 | 0.05 | 0.0463 |

**Table S13E.**

List of significant GO categories, based on set of male-biased significantly expressed genes detected in adults. The number of differentially expressed genes (significant) and the total number of annotated genes associated to a GO category are shown, as well as the number of expected genes. P-values were computed using the Fisher-elim algorithm.

| | GO.ID | Term | Annotated | Significant | Expected | elim_Fisher |
|---|---|---|---|---|---|---|
| 1 | GO:0003341 | cilium movement | 32 | 21 | 3.57 | 8.00E-10 |
| 2 | GO:0060271 | cilium assembly | 69 | 32 | 7.69 | 2.80E-07 |
| 3 | GO:0006508 | proteolysis | 551 | 100 | 61.4 | 1.80E-06 |
| 4 | GO:0044458 | motile cilium assembly | 5 | 5 | 0.56 | 1.70E-05 |
| 5 | GO:0030317 | flagellated sperm motility | 21 | 10 | 2.34 | 3.10E-05 |
| 6 | GO:0060294 | cilium movement involved in cell motility | 6 | 5 | 0.67 | 9.20E-05 |
| 7 | GO:0006030 | chitin metabolic process | 67 | 18 | 7.47 | 0.00027 |
| 8 | GO:0036158 | outer dynein arm assembly | 7 | 5 | 0.78 | 0.00029 |
| 9 | GO:0035082 | axoneme assembly | 23 | 12 | 2.56 | 0.00093 |
| 10 | GO:0006108 | malate metabolic process | 12 | 6 | 1.34 | 0.00096 |
| 11 | GO:0042073 | intraciliary transport | 12 | 6 | 1.34 | 0.00096 |
| 12 | GO:0009609 | response to symbiotic bacterium | 3 | 3 | 0.33 | 0.00138 |
| 13 | GO:0015740 | C4-dicarboxylate transport | 6 | 4 | 0.67 | 0.00191 |
| 14 | GO:0035310 | notum cell fate specification | 6 | 4 | 0.67 | 0.00191 |
| 15 | GO:0007413 | axonal fasciculation | 22 | 10 | 2.45 | 0.00214 |
| 16 | GO:0019722 | calcium-mediated signaling | 19 | 7 | 2.12 | 0.00313 |
| 17 | GO:0007605 | sensory perception of sound | 69 | 16 | 7.69 | 0.00314 |
| 18 | GO:0040003 | chitin-based cuticle development | 109 | 22 | 12.15 | 0.00386 |
| 19 | GO:0003333 | amino acid transmembrane transport | 30 | 9 | 3.34 | 0.00415 |
| 20 | GO:0006813 | potassium ion transport | 41 | 11 | 4.57 | 0.00417 |
| 21 | GO:0055085 | transmembrane transport | 443 | 72 | 49.37 | 0.00426 |
| 22 | GO:0097156 | fasciculation of motor neuron axon | 4 | 3 | 0.45 | 0.00506 |
| 23 | GO:0006919 | activation of cysteine-type endopeptidase activity | 16 | 6 | 1.78 | 0.00562 |
| 24 | GO:0015813 | L-glutamate transport | 8 | 4 | 0.89 | 0.00741 |
| 25 | GO:0042067 | establishment of ommatidial planar polarity | 57 | 13 | 6.35 | 0.00857 |
| 26 | GO:0007156 | homophilic cell adhesion via plasma membrane | 46 | 11 | 5.13 | 0.01051 |
| 27 | GO:0007503 | fat body development | 5 | 3 | 0.56 | 0.01159 |
| 28 | GO:0018095 | protein polyglutamylation | 5 | 3 | 0.56 | 0.01159 |
| 29 | GO:0045317 | equator specification | 5 | 3 | 0.56 | 0.01159 |
| 30 | GO:0060612 | adipose tissue development | 5 | 3 | 0.56 | 0.01159 |
| 31 | GO:0007602 | phototransduction | 53 | 12 | 5.91 | 0.01201 |
| 32 | GO:0018200 | peptidyl-glutamic acid modification | 9 | 4 | 1 | 0.01217 |
| 33 | GO:0007505 | adult fat body development | 2 | 2 | 0.22 | 0.0124 |
| 34 | GO:0008377 | light-induced release of internally sequestered Ca ion | 2 | 2 | 0.22 | 0.0124 |
| 35 | GO:0014043 | negative regulation of neuron maturation | 2 | 2 | 0.22 | 0.0124 |
| 36 | GO:0015810 | aspartate transport | 2 | 2 | 0.22 | 0.0124 |
| 37 | GO:0016539 | intein-mediated protein splicing | 2 | 2 | 0.22 | 0.0124 |
| 38 | GO:0030908 | protein splicing | 2 | 2 | 0.22 | 0.0124 |
| 39 | GO:0071454 | cellular response to anoxia | 2 | 2 | 0.22 | 0.0124 |
| 40 | GO:1904800 | negative regulation of neuron remodeling | 2 | 2 | 0.22 | 0.0124 |
| 41 | GO:0046189 | phenol-containing compound biosynthetic ... | 24 | 7 | 2.67 | 0.01311 |
| 42 | GO:0007548 | sex differentiation | 80 | 16 | 8.92 | 0.01384 |
| 43 | GO:0006011 | UDP-glucose metabolic process | 19 | 6 | 2.12 | 0.01423 |

| | | | | | | |
|---|---|---|---|---|---|---|
| 44 | GO:0042684 | cardioblast cell fate commitment | 14 | 5 | 1.56 | 0.01437 |
| 45 | GO:0035209 | pupal development | 30 | 8 | 3.34 | 0.01437 |
| 46 | GO:0009064 | glutamine family amino acid metabolic pr... | 36 | 9 | 4.01 | 0.01487 |
| 47 | GO:0007603 | phototransduction  visible light | 25 | 7 | 2.79 | 0.0165 |
| 48 | GO:0042381 | hemolymph coagulation | 10 | 4 | 1.11 | 0.01852 |
| 49 | GO:0043090 | amino acid import | 10 | 4 | 1.11 | 0.01852 |
| 50 | GO:0061331 | epithelial cell proliferation involved i... | 10 | 4 | 1.11 | 0.01852 |
| 51 | GO:2001013 | epithelial cell proliferation involved i... | 10 | 4 | 1.11 | 0.01852 |
| 52 | GO:0006941 | striated muscle contraction | 6 | 3 | 0.67 | 0.02128 |
| 53 | GO:0007561 | imaginal disc eversion | 6 | 3 | 0.67 | 0.02128 |
| 54 | GO:0009713 | catechol-containing compound biosyntheti... | 6 | 3 | 0.67 | 0.02128 |
| 55 | GO:0018149 | peptide cross-linking | 6 | 3 | 0.67 | 0.02128 |
| 56 | GO:0042136 | neurotransmitter biosynthetic process | 6 | 3 | 0.67 | 0.02128 |
| 57 | GO:0042423 | catecholamine biosynthetic process | 6 | 3 | 0.67 | 0.02128 |
| 58 | GO:0050912 | detection of chemical stimulus involved ... | 6 | 3 | 0.67 | 0.02128 |
| 59 | GO:0061448 | connective tissue development | 6 | 3 | 0.67 | 0.02128 |
| 60 | GO:0016056 | rhodopsin mediated signaling pathway | 21 | 6 | 2.34 | 0.02347 |
| 61 | GO:0071804 | cellular potassium ion transport | 21 | 6 | 2.34 | 0.02347 |
| 62 | GO:0071805 | potassium ion transmembrane transport | 21 | 6 | 2.34 | 0.02347 |
| 63 | GO:0071482 | cellular response to light stimulus | 33 | 8 | 3.68 | 0.0253 |
| 64 | GO:0035223 | leg disc pattern formation | 16 | 5 | 1.78 | 0.02598 |
| 65 | GO:0043052 | thermotaxis | 16 | 5 | 1.78 | 0.02598 |
| 66 | GO:0050878 | regulation of body fluid levels | 16 | 5 | 1.78 | 0.02598 |
| 67 | GO:0007599 | hemostasis | 11 | 4 | 1.23 | 0.02659 |
| 68 | GO:0050817 | coagulation | 11 | 4 | 1.23 | 0.02659 |
| 69 | GO:0098742 | cell-cell adhesion via plasma-membrane a... | 66 | 13 | 7.35 | 0.02816 |
| 70 | GO:1901657 | glycosyl compound metabolic process | 66 | 13 | 7.35 | 0.02816 |
| 71 | GO:0009584 | detection of visible light | 34 | 8 | 3.79 | 0.02997 |
| 72 | GO:0006814 | sodium ion transport | 28 | 7 | 3.12 | 0.0303 |
| 73 | GO:0009225 | nucleotide-sugar metabolic process | 28 | 7 | 3.12 | 0.0303 |
| 74 | GO:0009583 | detection of light stimulus | 60 | 12 | 6.69 | 0.03066 |
| 75 | GO:0048608 | reproductive structure development | 74 | 14 | 8.25 | 0.032 |
| 76 | GO:0061458 | reproductive system development | 74 | 14 | 8.25 | 0.032 |
| 77 | GO:0048864 | stem cell development | 17 | 5 | 1.89 | 0.03353 |
| 78 | GO:0051480 | regulation of cytosolic calcium ion conc... | 17 | 5 | 1.89 | 0.03353 |
| 79 | GO:0010951 | negative regulation of endopeptidase act... | 7 | 3 | 0.78 | 0.03419 |
| 80 | GO:0016486 | peptide hormone processing | 7 | 3 | 0.78 | 0.03419 |
| 81 | GO:0097090 | presynaptic membrane organization | 7 | 3 | 0.78 | 0.03419 |
| 82 | GO:0003009 | skeletal muscle contraction | 3 | 2 | 0.33 | 0.03445 |
| 83 | GO:0006828 | manganese ion transport | 3 | 2 | 0.33 | 0.03445 |
| 84 | GO:0034769 | basement membrane disassembly | 3 | 2 | 0.33 | 0.03445 |
| 85 | GO:0051283 | negative regulation of sequestering of c... | 3 | 2 | 0.33 | 0.03445 |
| 86 | GO:0060568 | regulation of peptide hormone processing | 3 | 2 | 0.33 | 0.03445 |
| 87 | GO:0060570 | negative regulation of peptide hormone p... | 3 | 2 | 0.33 | 0.03445 |
| 88 | GO:1903430 | negative regulation of cell maturation | 3 | 2 | 0.33 | 0.03445 |
| 89 | GO:0046692 | sperm competition | 12 | 4 | 1.34 | 0.03644 |
| 90 | GO:0098662 | inorganic cation transmembrane transport | 104 | 18 | 11.59 | 0.03748 |
| 91 | GO:0035215 | genital disc development | 42 | 9 | 4.68 | 0.03858 |
| 92 | GO:0007427 | epithelial cell migration | 36 | 8 | 4.01 | 0.041 |
| 93 | GO:0010002 | cardioblast differentiation | 18 | 5 | 2.01 | 0.0423 |
| 94 | GO:0048803 | imaginal disc-derived male genitalia mor... | 18 | 5 | 2.01 | 0.0423 |
| 95 | GO:0051346 | negative regulation of hydrolase activit... | 18 | 5 | 2.01 | 0.0423 |
| 96 | GO:0060911 | cardiac cell fate commitment | 18 | 5 | 2.01 | 0.0423 |
| 97 | GO:0008344 | adult locomotory behavior | 70 | 13 | 7.8 | 0.04331 |
| 98 | GO:0007485 | imaginal disc-derived male genitalia dev... | 24 | 6 | 2.67 | 0.04369 |
| 99 | GO:0007618 | mating | 92 | 16 | 10.25 | 0.04615 |
| 100 | GO:0042391 | regulation of membrane potential | 50 | 10 | 5.57 | 0.04617 |
| 101 | GO:0007320 | insemination | 13 | 4 | 1.45 | 0.04813 |

| | | | | | | |
|---|---|---|---|---|---|---|
| 102 | GO:0009312 | oligosaccharide biosynthetic process | 13 | 4 | 1.45 | 0.04813 |
| 103 | GO:0050962 | detection of light stimulus involved in ... | 13 | 4 | 1.45 | 0.04813 |

**Table S13F.**

List of top-200 most significant GO categories, based on set of female-biased significantly expressed genes detected in adults. The number of differentially expressed genes (significant) and the total number of annotated genes associated to a GO category are shown, as well as the number of expected genes. P-values were computed using the Fisher-elim algorithm.

| | GO.ID | Term | Annotated | Significant | Expected | elim_Fisher |
|---|---|---|---|---|---|---|
| 1 | GO:0002181 | cytoplasmic translation | 109 | 90 | 20.23 | 1.00E-30 |
| 2 | GO:0051298 | centrosome duplication | 81 | 47 | 15.04 | 2.20E-15 |
| 3 | GO:0000462 | maturation of SSU-rRNA | 33 | 28 | 6.13 | 4.50E-13 |
| 4 | GO:0042273 | ribosomal large subunit biogenesis | 51 | 45 | 9.47 | 5.20E-10 |
| 5 | GO:0010501 | RNA secondary structure unwinding | 30 | 21 | 5.57 | 9.80E-10 |
| 6 | GO:0006364 | rRNA processing | 116 | 96 | 21.53 | 2.50E-09 |
| 7 | GO:0009267 | cellular response to starvation | 103 | 45 | 19.12 | 3.00E-09 |
| 8 | GO:0007095 | mitotic G2 DNA damage checkpoint | 74 | 36 | 13.74 | 3.40E-09 |
| 9 | GO:0006334 | nucleosome assembly | 22 | 17 | 4.08 | 3.40E-09 |
| 10 | GO:0000398 | mRNA splicing  via spliceosome | 237 | 90 | 44 | 6.10E-09 |
| 11 | GO:0000470 | maturation of LSU-rRNA | 21 | 20 | 3.9 | 1.60E-08 |
| 12 | GO:0006378 | mRNA polyadenylation | 31 | 19 | 5.75 | 1.60E-07 |
| 13 | GO:0000027 | ribosomal large subunit assembly | 17 | 13 | 3.16 | 3.30E-07 |
| 14 | GO:0006418 | tRNA aminoacylation for protein translation | 47 | 26 | 8.72 | 4.40E-07 |
| 15 | GO:0000028 | ribosomal small subunit assembly | 13 | 11 | 2.41 | 4.70E-07 |
| 16 | GO:0030490 | maturation of SSU-rRNA | 41 | 36 | 7.61 | 1.20E-06 |
| 17 | GO:0034472 | snRNA 3'-end processing | 21 | 17 | 3.9 | 1.30E-06 |
| 18 | GO:0000463 | maturation of LSU-rRNA | 8 | 8 | 1.49 | 1.40E-06 |
| 19 | GO:0007307 | eggshell chorion gene amplification | 21 | 14 | 3.9 | 1.70E-06 |
| 20 | GO:0006270 | DNA replication initiation | 24 | 15 | 4.46 | 2.40E-06 |
| 21 | GO:0006606 | protein import into nucleus | 78 | 32 | 14.48 | 3.10E-06 |
| 22 | GO:0006413 | translational initiation | 56 | 32 | 10.4 | 3.30E-06 |
| 23 | GO:0007052 | mitotic spindle organization | 73 | 30 | 13.55 | 6.00E-06 |
| 24 | GO:0006261 | DNA-dependent DNA replication | 76 | 47 | 14.11 | 6.50E-06 |
| 25 | GO:0000460 | maturation of 5.8S rRNA | 19 | 17 | 3.53 | 1.00E-05 |
| 26 | GO:0022008 | neurogenesis | 1280 | 291 | 237.61 | 1.90E-05 |
| 27 | GO:0016321 | female meiosis chromosome segregation | 30 | 16 | 5.57 | 1.90E-05 |
| 28 | GO:0040018 | positive reg. of multicellular organism growth | 50 | 22 | 9.28 | 2.90E-05 |
| 29 | GO:0042274 | ribosomal small subunit biogenesis | 54 | 48 | 10.02 | 3.50E-05 |
| 30 | GO:0006272 | leading strand elongation | 6 | 6 | 1.11 | 4.10E-05 |
| 31 | GO:0070475 | rRNA base methylation | 6 | 6 | 1.11 | 4.10E-05 |
| 32 | GO:0031125 | rRNA 3'-end processing | 8 | 7 | 1.49 | 5.00E-05 |
| 33 | GO:0000724 | double-strand break repair | 39 | 18 | 7.24 | 7.20E-05 |
| 34 | GO:0007076 | mitotic chromosome condensation | 21 | 12 | 3.9 | 8.90E-05 |
| 35 | GO:0006406 | mRNA export from nucleus | 30 | 18 | 5.57 | 9.20E-05 |
| 36 | GO:0032543 | mitochondrial translation | 103 | 35 | 19.12 | 0.00013 |
| 37 | GO:0001731 | formation of translation preinitiation c... | 11 | 8 | 2.04 | 0.00013 |
| 38 | GO:0006379 | mRNA cleavage | 19 | 11 | 3.53 | 0.00015 |
| 39 | GO:0006260 | DNA replication | 125 | 67 | 23.2 | 0.00016 |
| 40 | GO:0000154 | rRNA modification | 20 | 18 | 3.71 | 0.00018 |
| 41 | GO:0051445 | regulation of meiotic cell cycle | 9 | 7 | 1.67 | 0.00019 |
| 42 | GO:0006458 | 'de novo' protein folding | 14 | 9 | 2.6 | 0.00021 |
| 43 | GO:0000055 | ribosomal large subunit export from nucl... | 5 | 5 | 0.93 | 0.00022 |
| 44 | GO:0000447 | endonucleolytic cleavage in ITS1 to sepa... | 5 | 5 | 0.93 | 0.00022 |

| | | | | | | |
|---|---|---|---|---|---|---|
| 45 | GO:0031118 | rRNA pseudouridine synthesis | 5 | 5 | 0.93 | 0.00022 |
| 46 | GO:0034475 | U4 snRNA 3'-end processing | 5 | 5 | 0.93 | 0.00022 |
| 47 | GO:0071051 | polyadenylation-dependent snoRNA 3'-end ... | 5 | 5 | 0.93 | 0.00022 |
| 48 | GO:0051028 | mRNA transport | 59 | 32 | 10.95 | 0.00023 |
| 49 | GO:0000469 | cleavage involved in rRNA processing | 15 | 14 | 2.78 | 0.00023 |
| 50 | GO:0071028 | nuclear mRNA surveillance | 7 | 6 | 1.3 | 0.00024 |
| 51 | GO:0045132 | meiotic chromosome segregation | 57 | 31 | 10.58 | 0.00025 |
| 52 | GO:0030717 | karyosome formation | 29 | 14 | 5.38 | 0.00026 |
| 53 | GO:0061077 | chaperone-mediated protein folding | 29 | 14 | 5.38 | 0.00026 |
| 54 | GO:0006289 | nucleotide-excision repair | 26 | 13 | 4.83 | 0.00027 |
| 55 | GO:0006298 | mismatch repair | 12 | 8 | 2.23 | 0.00034 |
| 56 | GO:0016925 | protein sumoylation | 12 | 8 | 2.23 | 0.00034 |
| 57 | GO:0046011 | regulation of oskar mRNA translation | 12 | 8 | 2.23 | 0.00034 |
| 58 | GO:0042254 | ribosome biogenesis | 176 | 137 | 32.67 | 0.00036 |
| 59 | GO:0051301 | cell division | 304 | 80 | 56.43 | 0.00041 |
| 60 | GO:0008298 | intracellular mRNA localization | 78 | 27 | 14.48 | 0.00053 |
| 61 | GO:0006284 | base-excision repair | 10 | 7 | 1.86 | 0.00053 |
| 62 | GO:0006999 | nuclear pore organization | 10 | 7 | 1.86 | 0.00053 |
| 63 | GO:0006398 | mRNA 3'-end processing by stem-loop bind... | 8 | 6 | 1.49 | 0.0008 |
| 64 | GO:0045005 | DNA-dependent DNA replication maintenanc... | 8 | 6 | 1.49 | 0.0008 |
| 65 | GO:0034587 | piRNA metabolic process | 16 | 9 | 2.97 | 0.00083 |
| 66 | GO:0000387 | spliceosomal snRNP assembly | 19 | 10 | 3.53 | 0.00085 |
| 67 | GO:0000819 | sister chromatid segregation | 109 | 45 | 20.23 | 0.00086 |
| 68 | GO:0010467 | gene expression | 2041 | 651 | 378.88 | 0.00106 |
| 69 | GO:0031990 | mRNA export from nucleus in response to ... | 6 | 5 | 1.11 | 0.00111 |
| 70 | GO:0034427 | nuclear-transcribed mRNA catabolic proce... | 6 | 5 | 1.11 | 0.00111 |
| 71 | GO:0031124 | mRNA 3'-end processing | 45 | 28 | 8.35 | 0.00114 |
| 72 | GO:0007348 | regulation of syncytial blastoderm mitot... | 4 | 4 | 0.74 | 0.00118 |
| 73 | GO:0031119 | tRNA pseudouridine synthesis | 4 | 4 | 0.74 | 0.00118 |
| 74 | GO:0031120 | snRNA pseudouridine synthesis | 4 | 4 | 0.74 | 0.00118 |
| 75 | GO:0036228 | protein targeting to nuclear inner membr... | 4 | 4 | 0.74 | 0.00118 |
| 76 | GO:0045948 | positive regulation of translational ini... | 4 | 4 | 0.74 | 0.00118 |
| 77 | GO:0002097 | tRNA wobble base modification | 9 | 6 | 1.67 | 0.00203 |
| 78 | GO:0010032 | meiotic chromosome condensation | 9 | 6 | 1.67 | 0.00203 |
| 79 | GO:0006360 | transcription from RNA polymerase I prom... | 18 | 9 | 3.34 | 0.00246 |
| 80 | GO:0006383 | transcription from RNA polymerase III pr... | 28 | 12 | 5.2 | 0.00255 |
| 81 | GO:0006338 | chromatin remodeling | 98 | 30 | 18.19 | 0.00256 |
| 82 | GO:0007094 | mitotic spindle assembly checkpoint | 15 | 8 | 2.78 | 0.00257 |
| 83 | GO:0007088 | regulation of mitotic nuclear division | 62 | 25 | 11.51 | 0.00311 |
| 84 | GO:0044772 | mitotic cell cycle phase transition | 165 | 68 | 30.63 | 0.00319 |
| 85 | GO:0071428 | rRNA-containing ribonucleoprotein comple... | 12 | 10 | 2.23 | 0.00325 |
| 86 | GO:0006273 | lagging strand elongation | 7 | 5 | 1.3 | 0.00329 |
| 87 | GO:0007144 | female meiosis I | 7 | 5 | 1.3 | 0.00329 |
| 88 | GO:0016075 | rRNA catabolic process | 7 | 5 | 1.3 | 0.00329 |
| 89 | GO:0036297 | interstrand cross-link repair | 7 | 5 | 1.3 | 0.00329 |
| 90 | GO:0032508 | DNA duplex unwinding | 22 | 10 | 4.08 | 0.00345 |
| 91 | GO:0000077 | DNA damage checkpoint | 93 | 45 | 17.26 | 0.00346 |
| 92 | GO:0008033 | tRNA processing | 73 | 36 | 13.55 | 0.00356 |
| 93 | GO:0000381 | regulation of alternative mRNA splicing | 71 | 23 | 13.18 | 0.00356 |
| 94 | GO:0007346 | regulation of mitotic cell cycle | 225 | 88 | 41.77 | 0.00371 |
| 95 | GO:0006405 | RNA export from nucleus | 57 | 35 | 10.58 | 0.0039 |
| 96 | GO:0006414 | translational elongation | 19 | 9 | 3.53 | 0.00391 |
| 97 | GO:0090502 | RNA phosphodiester bond hydrolysis | 18 | 14 | 3.34 | 0.00418 |
| 98 | GO:0000076 | DNA replication checkpoint | 13 | 7 | 2.41 | 0.0045 |
| 99 | GO:0016572 | histone phosphorylation | 13 | 7 | 2.41 | 0.0045 |
| 100 | GO:0030488 | tRNA methylation | 13 | 7 | 2.41 | 0.0045 |
| 101 | GO:0031123 | RNA 3'-end processing | 71 | 48 | 13.18 | 0.00458 |
| 102 | GO:0006473 | protein acetylation | 85 | 26 | 15.78 | 0.00485 |

| | | | | | | |
|---|---|---|---|---|---|---|
| 103 | GO:0051304 | chromosome separation | 46 | 23 | 8.54 | 0.00486 |
| 104 | GO:0000466 | maturation of 5.8S rRNA from tricistroni... | 10 | 9 | 1.86 | 0.00498 |
| 105 | GO:0001113 | transcriptional open complex formation a... | 5 | 4 | 0.93 | 0.00504 |
| 106 | GO:0035247 | peptidyl-arginine omega-N-methylation | 5 | 4 | 0.93 | 0.00504 |
| 107 | GO:0070525 | tRNA threonylcarbamoyladenosine metaboli... | 5 | 4 | 0.93 | 0.00504 |
| 108 | GO:0006396 | RNA processing | 552 | 271 | 102.47 | 0.00517 |
| 109 | GO:0030261 | chromosome condensation | 53 | 26 | 9.84 | 0.00573 |
| 110 | GO:0006325 | chromatin organization | 353 | 105 | 65.53 | 0.00581 |
| 111 | GO:0006401 | RNA catabolic process | 84 | 29 | 15.59 | 0.00606 |
| 112 | GO:0044260 | cellular macromolecule metabolic process | 3209 | 883 | 595.7 | 0.00619 |
| 113 | GO:0000479 | endonucleolytic cleavage of tricistronic... | 8 | 8 | 1.49 | 0.00633 |
| 114 | GO:0043144 | snoRNA processing | 8 | 8 | 1.49 | 0.00633 |
| 115 | GO:0033260 | nuclear DNA replication | 11 | 8 | 2.04 | 0.00633 |
| 116 | GO:0001682 | tRNA 5'-leader removal | 3 | 3 | 0.56 | 0.00638 |
| 117 | GO:0002192 | IRES-dependent translational initiation | 3 | 3 | 0.56 | 0.00638 |
| 118 | GO:0006438 | valyl-tRNA aminoacylation | 3 | 3 | 0.56 | 0.00638 |
| 119 | GO:0006449 | regulation of translational termination | 3 | 3 | 0.56 | 0.00638 |
| 120 | GO:0006719 | juvenile hormone catabolic process | 3 | 3 | 0.56 | 0.00638 |
| 121 | GO:0035044 | sperm aster formation | 3 | 3 | 0.56 | 0.00638 |
| 122 | GO:0051257 | meiotic spindle midzone assembly | 3 | 3 | 0.56 | 0.00638 |
| 123 | GO:1990481 | mRNA pseudouridine synthesis | 3 | 3 | 0.56 | 0.00638 |
| 124 | GO:2000765 | regulation of cytoplasmic translation | 3 | 3 | 0.56 | 0.00638 |
| 125 | GO:0006302 | double-strand break repair | 63 | 28 | 11.69 | 0.00689 |
| 126 | GO:0000712 | resolution of meiotic recombination inte... | 8 | 5 | 1.49 | 0.00744 |
| 127 | GO:0000731 | DNA synthesis involved in DNA repair | 8 | 5 | 1.49 | 0.00744 |
| 128 | GO:1902969 | mitotic DNA replication | 8 | 5 | 1.49 | 0.00744 |
| 129 | GO:0016570 | histone modification | 185 | 52 | 34.34 | 0.00748 |
| 130 | GO:0006281 | DNA repair | 215 | 80 | 39.91 | 0.00749 |
| 131 | GO:0006354 | DNA-templated transcription elongation | 39 | 14 | 7.24 | 0.00787 |
| 132 | GO:0022618 | ribonucleoprotein complex assembly | 116 | 60 | 21.53 | 0.00794 |
| 133 | GO:0007100 | mitotic centrosome separation | 11 | 6 | 2.04 | 0.00796 |
| 134 | GO:0042023 | DNA endoreduplication | 21 | 9 | 3.9 | 0.00874 |
| 135 | GO:0051306 | mitotic sister chromatid separation | 33 | 16 | 6.13 | 0.01007 |
| 136 | GO:0031047 | gene silencing by RNA | 73 | 22 | 13.55 | 0.01099 |
| 137 | GO:0006412 | translation | 448 | 217 | 83.16 | 0.01139 |
| 138 | GO:0006475 | internal protein amino acid acetylation | 82 | 24 | 15.22 | 0.01192 |
| 139 | GO:0018393 | internal peptidyl-lysine acetylation | 82 | 24 | 15.22 | 0.01192 |
| 140 | GO:0018394 | peptidyl-lysine acetylation | 82 | 24 | 15.22 | 0.01192 |
| 141 | GO:0031145 | anaphase-promoting complex-dependent cat... | 15 | 7 | 2.78 | 0.01197 |
| 142 | GO:0000956 | nuclear-transcribed mRNA catabolic proce... | 57 | 22 | 10.58 | 0.01238 |
| 143 | GO:0043486 | histone exchange | 22 | 9 | 4.08 | 0.0124 |
| 144 | GO:0031167 | rRNA methylation | 12 | 10 | 2.23 | 0.01272 |
| 145 | GO:0000054 | ribosomal subunit export from nucleus | 11 | 9 | 2.04 | 0.01275 |
| 146 | GO:0033750 | ribosome localization | 11 | 9 | 2.04 | 0.01275 |
| 147 | GO:0033753 | establishment of ribosome localization | 11 | 9 | 2.04 | 0.01275 |
| 148 | GO:0001109 | promoter clearance during DNA-templated ... | 6 | 4 | 1.11 | 0.01289 |
| 149 | GO:0001111 | promoter clearance from RNA polymerase I... | 6 | 4 | 1.11 | 0.01289 |
| 150 | GO:0009262 | deoxyribonucleotide metabolic process | 6 | 4 | 1.11 | 0.01289 |
| 151 | GO:0016233 | telomere capping | 6 | 4 | 1.11 | 0.01289 |
| 152 | GO:0034508 | centromere complex assembly | 6 | 4 | 1.11 | 0.01289 |
| 153 | GO:0007056 | spindle assembly involved in female meio... | 12 | 6 | 2.23 | 0.01345 |
| 154 | GO:0008069 | dorsal/ventral axis specification | 12 | 6 | 2.23 | 0.01345 |
| 155 | GO:0007062 | sister chromatid cohesion | 26 | 10 | 4.83 | 0.01389 |
| 156 | GO:0000726 | non-recombinational repair | 9 | 5 | 1.67 | 0.01421 |
| 157 | GO:0045003 | double-strand break repair via synthesis... | 9 | 5 | 1.67 | 0.01421 |
| 158 | GO:0051225 | spindle assembly | 65 | 22 | 12.07 | 0.01421 |
| 159 | GO:0051276 | chromosome organization | 552 | 183 | 102.47 | 0.01457 |
| 160 | GO:0007049 | cell cycle | 816 | 275 | 151.48 | 0.01488 |

| | | | | | | |
|---|---|---|---|---|---|---|
| 161 | GO:0090307 | mitotic spindle assembly | 30 | 11 | 5.57 | 0.0149 |
| 162 | GO:0007131 | reciprocal meiotic recombination | 24 | 12 | 4.46 | 0.01767 |
| 163 | GO:0006275 | regulation of DNA replication | 16 | 7 | 2.97 | 0.01792 |
| 164 | GO:0000086 | G2/M transition of mitotic cell cycle | 94 | 44 | 17.45 | 0.01898 |
| 165 | GO:0016573 | histone acetylation | 81 | 23 | 15.04 | 0.01965 |
| 166 | GO:0006611 | protein export from nucleus | 54 | 33 | 10.02 | 0.01966 |
| 167 | GO:0000082 | G1/S transition of mitotic cell cycle | 35 | 12 | 6.5 | 0.01975 |
| 168 | GO:0044843 | cell cycle G1/S phase transition | 35 | 12 | 6.5 | 0.01975 |
| 169 | GO:0051321 | meiotic cell cycle | 188 | 73 | 34.9 | 0.02082 |
| 170 | GO:0071166 | ribonucleoprotein complex localization | 45 | 30 | 8.35 | 0.02107 |
| 171 | GO:0007184 | SMAD protein import into nucleus | 13 | 6 | 2.41 | 0.02112 |
| 172 | GO:0007315 | pole plasm assembly | 60 | 18 | 11.14 | 0.02123 |
| 173 | GO:0043628 | ncRNA 3'-end processing | 28 | 22 | 5.2 | 0.0213 |
| 174 | GO:1901990 | regulation of mitotic cell cycle phase t... | 146 | 61 | 27.1 | 0.02151 |
| 175 | GO:0018195 | peptidyl-arginine modification | 9 | 7 | 1.67 | 0.02184 |
| 176 | GO:0000256 | allantoin catabolic process | 4 | 3 | 0.74 | 0.02199 |
| 177 | GO:0000451 | rRNA 2'-O-methylation | 4 | 3 | 0.74 | 0.02199 |
| 178 | GO:0000706 | meiotic DNA double-strand break processi... | 4 | 3 | 0.74 | 0.02199 |
| 179 | GO:0000729 | DNA double-strand break processing | 4 | 3 | 0.74 | 0.02199 |
| 180 | GO:0006419 | alanyl-tRNA aminoacylation | 4 | 3 | 0.74 | 0.02199 |
| 181 | GO:0006429 | leucyl-tRNA aminoacylation | 4 | 3 | 0.74 | 0.02199 |
| 182 | GO:0006432 | phenylalanyl-tRNA aminoacylation | 4 | 3 | 0.74 | 0.02199 |
| 183 | GO:0009263 | deoxyribonucleotide biosynthetic process | 4 | 3 | 0.74 | 0.02199 |
| 184 | GO:0010457 | centriole-centriole cohesion | 4 | 3 | 0.74 | 0.02199 |
| 185 | GO:0019919 | peptidyl-arginine methylation | 4 | 3 | 0.74 | 0.02199 |
| 186 | GO:0033567 | DNA replication | 4 | 3 | 0.74 | 0.02199 |
| 187 | GO:0033683 | nucleotide-excision repair | 4 | 3 | 0.74 | 0.02199 |
| 188 | GO:0034969 | histone arginine methylation | 4 | 3 | 0.74 | 0.02199 |
| 189 | GO:0040020 | regulation of meiotic nuclear division | 4 | 3 | 0.74 | 0.02199 |
| 190 | GO:0043570 | maintenance of DNA repeat elements | 4 | 3 | 0.74 | 0.02199 |
| 191 | GO:0043605 | cellular amide catabolic process | 4 | 3 | 0.74 | 0.02199 |
| 192 | GO:0051341 | regulation of oxidoreductase activity | 4 | 3 | 0.74 | 0.02199 |
| 193 | GO:0070920 | regulation of production of small RNA in... | 4 | 3 | 0.74 | 0.02199 |
| 194 | GO:0071033 | nuclear retention of pre-mRNA at the sit... | 4 | 3 | 0.74 | 0.02199 |
| 195 | GO:0097010 | eukaryotic translation initiation factor... | 4 | 3 | 0.74 | 0.02199 |
| 196 | GO:0018205 | peptidyl-lysine modification | 144 | 42 | 26.73 | 0.0228 |
| 197 | GO:0007316 | pole plasm RNA localization | 52 | 16 | 9.65 | 0.02284 |
| 198 | GO:0019094 | pole plasm mRNA localization | 52 | 16 | 9.65 | 0.02284 |
| 199 | GO:0006399 | tRNA metabolic process | 124 | 65 | 23.02 | 0.02304 |
| 200 | GO:0006367 | transcription initiation from RNA polyme... | 83 | 26 | 15.41 | 0.02332 |

**Table S14.**

List of MSL complex core genes and modulators. The p-value (before FDR adjustment) and p-adj (after FDR adjustment) were computed using DESEQ2 (*****: $p < 0.0001$; ****: $p < 0.001$; ***: $p < 0.01$; **: $p < 0.05$; *: $p < 0.15$). Data based on *de novo* transcriptome assembly.

| Gene | Protein | p-value (before FDR) | p-adj | Up-regulated (adult *L. sinapis*) |
|---|---|---|---|---|
| ***MSL complex*** | | | | |
| MSL2 | Male-specific lethal-2 | ***** | **** | female |
| MLE | Dosage compensation regulator (aka Maleless) | ** | * | female |
| MOF | Males-absent on the first (aka Histone acetyl transferase) | * | > 0.15 | female |
| MSL1 | Male-specific lethal-1 | ** | * | female |
| MSL3 | Male-specific lethal-3 | > 0.15 | > 0.15 | - |
| ***Dosage compensation modulators*** | | | | |
| TOPO2 | DNA topoisomerase 2 | *** | ** | female |
| LOQS | Loquacious | ***** | **** | female |

**Table S15.**

Results of *BLASTX* analysis of *L. sinapis* homologs of the five MSL complex core genes.

| Gene | Target | | e-value | Identity | Query cover | Query | Target |
|---|---|---|---|---|---|---|---|
| | Species | NCBI Sequence ID | | (%) | (%) | transcript length | protein length |
| MSL-2 | *Drosophila melanogaster* | ABU96719.1 | 2E-16 | 32 | 30 | 1869 bp | 773 aa |
| | *Danaus plexippus* | OWR47715.1 | 0E+00 | 71 | 69 | 1869 bp | 428 aa |
| | *Papilio xuthus* | KPI94931.1 | 2E-135 | 61 | 69 | 1869 bp | 439 aa |
| | *Bombyx mori* | NP_001093307.1 | 1E-168 | 70 | 69 | 1869 bp | 434 aa |
| MLE | *Drosophila melanogaster* | AAC41573.1 | 0E+00 | 58 | 84 | 4152 bp | 1293 aa |
| | *Danaus plexippus* | OWR48047.1 | 0E+00 | 78 | 52 | 4152 bp | 804 aa |
| | *Papilio xuthus* | KPI93703.1 | 0E+00 | 77 | 85 | 4152 bp | 1295 aa |
| | *Bombyx mori* | NP_001093309.1 | 0E+00 | 80 | 85 | 4152 bp | 1308 aa |
| MOF | *Drosophila melanogaster* | ABV82533.1 | 2E-160 | 55 | 72 | 1606 bp | 794 aa |
| | *Danaus plexippus* | OWR44944.1 | 2E-147 | 81 | 45 | 1606 bp | 243 aa |
| | *Papilio xuthus* | KPI93089.1 | 0E+00 | 81 | 82 | 1606 bp | 442 aa |
| | *Bombyx mori* | NP_001093305.1 | 0E+00 | 82 | 82 | 1606 bp | 442 aa |
| MSL-1 | *Drosophila melanogaster* | ABV82538.1 | 1E-03 | 30 | 12 | 2887 bp | 1049 aa |
| | *Danaus plexippus* | OWR53188.1 | 5E-99 | 50 | 64 | 2887 bp | 766 aa |
| | *Papilio xuthus* | KPI96053.1 | 7E-13 | 86 | 25 | 2887 bp | 1761 aa |
| | *Bombyx mori* | NP_001093306.1 | 3E-85 | 44 | 59 | 2887 bp | 814 aa |
| MSL-3 | *Drosophila melanogaster* | NP_523951.1 | 7E-36 | 33 | 43 | 2193 bp | 512 aa |
| | *Danaus plexippus* | OWR50737.1 | 0E+00 | 58 | 80 | 2193 bp | 560 aa |
| | *Papilio xuthus* | KPI94219.1 | 2E-125 | 64 | 64 | 2193 bp | 854 aa |
| | *Bombyx mori* | NP_001093308.1 | 2E-141 | 62 | 65 | 2193 bp | 554 aa |
